# GADMA: Genetic algorithm for inferring demographic history of multiple populations from allele frequency spectrum data

**DOI:** 10.1093/gigascience/giaa005

**Published:** 2020-02-29

**Authors:** Ekaterina Noskova, Vladimir Ulyantsev, Klaus-Peter Koepfli, Stephen J O’Brien, Pavel Dobrynin

**Affiliations:** 1 Computer Technologies Laboratory, ITMO University, 49 Kronverkskiy Pr., St. Petersburg 197101, Russian Federation; 2 Smithsonian Conservation Biology Institute, Center for Species Survival, National Zoological Park, 3001 Connecticut Ave., NW Washington, D.C. 20008, USA; 3 Guy Harvey Oceanographic Center, Nova Southeastern University Ft. Lauderdale, 8000 North Ocean Drive, Ft. Lauderdale, Florida 33004, USA

**Keywords:** demographic inference, population genetics, genetic algorithm, allele frequency spectrum, genomics

## Abstract

**Background:**

The demographic history of any population is imprinted in the genomes of the individuals that make up the population. One of the most popular and convenient representations of genetic information is the allele frequency spectrum (AFS), the distribution of allele frequencies in populations. The joint AFS is commonly used to reconstruct the demographic history of multiple populations, and several methods based on diffusion approximation (e.g., ∂a∂i) and ordinary differential equations (e.g., moments) have been developed and applied for demographic inference. These methods provide an opportunity to simulate AFS under a variety of researcher-specified demographic models and to estimate the best model and associated parameters using likelihood-based local optimizations. However, there are no known algorithms to perform global searches of demographic models with a given AFS.

**Results:**

Here, we introduce a new method that implements a global search using a genetic algorithm for the automatic and unsupervised inference of demographic history from joint AFS data. Our method is implemented in the software GADMA (Genetic Algorithm for Demographic Model Analysis, https://github.com/ctlab/GADMA).

**Conclusions:**

We demonstrate the performance of GADMA by applying it to sequence data from humans and non-model organisms and show that it is able to automatically infer a demographic model close to or even better than the one that was previously obtained manually. Moreover, GADMA is able to infer multiple demographic models at different local optima close to the global one, providing a larger set of possible scenarios to further explore demographic history.

## Introduction

To understand the evolution of species and their populations, it is important to understand what events occurred in their past and when. The genetic diversity and structure of species are shaped by the combined processes of changes in effective population size, population divergence, and/or migration (gene flow) operating over the course of thousands of generations. Records of population history are imprinted in the genomes of individuals within species, and this history can be inferred using a variety of algorithmic and statistical methods. With the rise of next-generation sequencing technologies and abundant genome data, it has become possible to explore complex and parameter-rich demographic models that include the estimation of mutation rate, changes in effective population size, nonrandom mating, admixture, and selection [1,2]. However, given the infinitely large number of permutations at which these processes operate over various time intervals, there is no method that can guarantee to find the demographic model that best fits the observed data.

One of the primary methods for inferring demographic models from genomic data is based on the allele frequency spectrum (AFS), also known as the site frequency spectrum [2,3]. In essence, the AFS describes the distribution of derived allele frequencies of bi-allelic loci (single-nucleotide variants [SNVs]) in a population or sample of populations based on the number of sequenced chromosomes [4]. An AFS can provide information about how the populations developed based on observed genetic variation sampled from current individuals of those populations. Many studies have been devoted to testing and understanding the behavior of AFS under different demographic scenarios [5–9].

**Figure 1:**
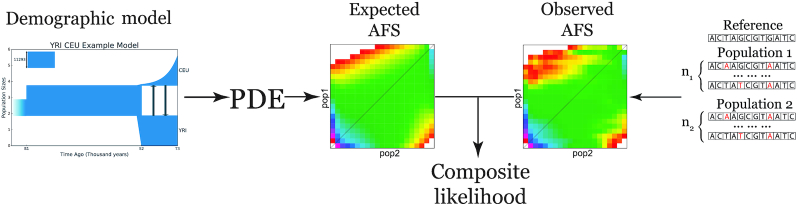
General scheme of ∂a∂i. To compare a demographic model and the real observed AFS, ∂a∂i extracts the expected AFS from the demographic model using a numerical solution of the partial differential equation (PDE) that corresponds to that demographic model, and calculates the composite likelihood between the expected and observed allele frequency spectrum.

Two of the most popular methods of historical demographic inference based on AFS are the faster continuous-time sequential Markovian coalescent approximation (fastsimcoal2, [10]) and the diffusion approximation (∂a∂i, [11]). fastsimcoal2 can successfully handle >3 populations, but it is computationally challenging because it simulates multiple AFS simultaneously to estimate the most stable one. ∂a∂i simulates AFS using a numerical solution of the partial diffusion equation (PDE), which corresponds to the presented demographic model and then provides the likelihood of the model (Fig. 1). Unfortunately, PDE leads to some computational difficulties associated with analyses of complicated demographic models and large sample sizes. As a result, ∂a∂i can only handle ≤3 populations. More recently, 2 new methods called "moments" [12] and "Moran Models of Inference" (momi2 [13]) have been introduced. moments, like ∂a∂i, is based on 2 models of population genetics: the Wright-Fisher generation model and the infinite sites mutational model, whereas momi2 is based on another generation model—the Moran model [14]. Because momi2 is a new method, we have not considered it in the present study. moments uses ordinary differential equations to simulate AFS, which is faster and more stable than diffusion approximation in ∂a∂i, based on simulations comparing the 2 methods [12]. moments presents a tradeoff between speed and accuracy in AFS-based demographic inference, can handle ≤5 populations, and provides the same interface as ∂a∂i.

Ideally, researchers seek to find the model of demographic history that best describes or “fits” their data. ∂a∂i and moments provide an opportunity to run multiple optimizations to help fit parameters of a given demographic model that maximizes the value of the composite likelihood. But optimizations based on gradient descent, e.g., the Broyden-Fletcher-Goldfarb-Shanno (BFGS) algorithm [15–18] or its modifications, use numerical differentiation and are ineffective in practice, because of the complex structure of the search space for demographic models. Optimizations may also be inefficient owing to another set of methods that offer existing solutions based on local search algorithms without gradients, such as the Nedler and Mead method [19] or Powell method [20]. As a result, all existing optimizations find local optima close to the initial values and require many runs to be performed using different initial model parameters, most of which are unknown or lack empirical data. Despite these drawbacks, ∂a∂i and moments are efficient instruments because they can simulate an AFS from any demographic model. In other words, the problem of finding a demographic model from the AFS is the inverse problem, which can be solved by solving the direct problem, i.e., simulation of the AFS from a given demographic model, with approximate numerical methods, such as diffusion or moments approximations. The lack of accurate and rapid differentiation and the complex structure of the search space led us to consider the use of global optimization methods, such as the genetic algorithm (GA).

The GA [21] is one of the most efficient heuristic algorithms for global searching of complex and rich parameter space. Its primary application is optimization of a fitness function, which either is not differentiable or cannot be differentiated in a sufficiently effective way, e.g., when the function is not representable in “closed-form expression.” GA is based on the principle of evolution and simulates natural selection using operations of “mutation” and “crossover,” which ideally results in the most adapted individual, the one that has the best value of the fitness function. The versatility of the GA has led to its wide application, including reconstruction of phylogenetic trees [22], ancestral genome composition inference [23], and evolutionary biology in general [24].

In this article we present a new method based on the GA to automatically infer the best-fitting demographic model from AFS data for 2 or 3 populations. Our method assumes the ability to simulate the AFS from the demographic model, e.g., using either ∂a∂i or moments. The GA framework overcomes limitations of local search optimizations and is more flexible in handling the complexity of demographic models by allowing an increase in the number of parameters and estimation of parameters such as functions of population size changes that are sudden, linear, or exponential. We have implemented our method in the software GADMA (Genetic Algorithm for Demographic Model Analysis), which is written in Python and available from Github (https://github.com/ctlab/GADMA).

## Materials and Methods

This section provides definitions of the AFS and the composite likelihood scheme that is used in existing optimizations (∂a∂i and moments) and implemented in our method. After this background, the problem of demographic model search from observed AFS data is formulated in terms of computer science. We then introduce a developed representation of a demographic model for a general approach to our method, including the GA with its operations of “mutation” and “crossover.”

### Basic definitions and concepts

**Figure 2:**
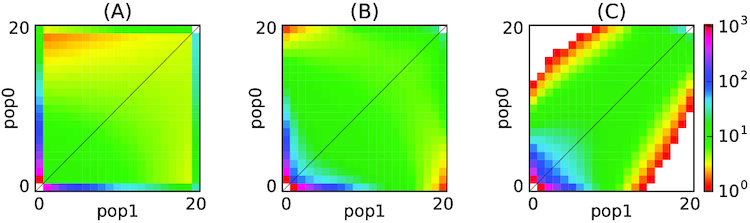
Examples of different allele frequency spectra. (A) Isolation of a population after divergence; (B) Low symmetrical rates of migrations between populations following divergence; (C) High asymmetrical rates of migrations between populations following divergence.

Assume there are *P* populations and for each population *i* there exists information about *n_i_* chromosomes. The AFS is the *P*-dimensional array *A*, where each entry $A[d_1,\dots , d_P] \in \mathbb {N}$, *d_i_* ∈ [0, *n_i_*], ∀*i* ∈ [1, *P*] records the number of SNVs (relative to the common reference genome), which are exactly seen at *d*_1_ chromosomes from population 1, *d*_2_ chromosomes from population 2, … and *d_P_* chromosomes from population *P*. For example, if we have 2 populations, then the AFS is a 2D matrix, where *A*[*i, j*] represents the number of polymorphisms that occurred exactly in *i* individuals in the first population and in *j* individuals in the second population (Fig. 1). Several examples of AFS are presented in Fig. 2.

Assume that we can simulate the AFS *M* from the proposed demographic model. Assuming no linkage between derived alleles, each element of the AFS *S*[*d*_1_, …, *d_P_*] is an independent Poisson value with a mean equal to *M*[*d*_1_, …, *d_P_*]. We then calculate the likelihood—the probability of obtaining the observed spectrum *S*, if the expectation spectrum is *M*, as the product of (*n*_1_ + 1)(*n*_2_ + 1)…(*n_P_* + 1) Poisson likelihood functions:

$$\begin{eqnarray*}
\mathcal {L}(M|S) = \prod _{\substack{d_i=0,.. , n_i,\\
i=1..P}} \frac{e^{-M[d_1,\dots ,d_P]} M[d_1,\dots ,d_P]^{S[d_1,\dots ,d_P]}}{S[d_1,\dots ,d_P]\!}.
\end{eqnarray*}$$

In the case of linked alleles, $\mathcal {L}(M|S)$ is the composite likelihood. Demographic models inferred by ∂a∂i and moments can be compared by computing the $\log (\mathcal {L}(M | S))$. Because $\mathcal {L}(M|S) \in [0, 1]$, then $\log (\mathcal {L}(M | S)) \in [-\inf , 0 ]$ and the greater is the log-likelihood, the better the model fits the observed AFS. In this article, $\log (\mathcal {L}(M | S))$ was chosen as the fitness function of the GA, as discussed below.

### Formulation of the problem

Consider a function *f*(Θ, *A, C*) that takes the parameters $\Theta = \lbrace \theta ^k\rbrace _{k=1}^{N_\Theta },\quad \theta ^k \in \mathbb {R}$, the AFS $A \in \mathbb {R}^{P\times P}$, the set of constants $C = \lbrace c^k\rbrace _{k=1}^{N_C}$, and returns the measure of the correspondence between the parameters Θ and the AFS, *A*.

The function *f*(Θ, *A, C*) builds a demographic model with respect to the parameters Θ that unambiguously determine it, calculates the expected AFS *M* with respect to the constants *C*, and then determines the degree of similarity between *M* and the observed *A* by the composite likelihood. The constants can be various parameters of algorithms for calculating the expected AFS, such as grid sizes for the numerical solution of a differential equation in ∂a∂i, or population model parameters, including the average number of new mutated sites per individual in a generation θ_0_ for the infinite-sites mutational model, or the time *T_g_* for 1 generation in the Wright-Fisher model. The function *f* can have different implementation details. Here ∂a∂i and moments were selected for this purpose.

The purpose of this work is to develop an algorithm to search for the demographic model that best corresponds to the observed AFS. Formally, the problem can be formulated as follows:

**Input**
  - $A \in \mathbb {R}^{P\times P}$—the *P*-dimensional array, *P* ∈ {2, 3}.
  - $C=\lbrace c_k\rbrace _{k=1}^{N_C}$—the set of constants.
**Output**
  - The set $\Theta \in \mathbb {R}^{N_{\Theta }}$ of values that maximize the value of *f*:
    $$\begin{eqnarray*}
\Theta :\quad f(\Theta , A, C) \rightarrow \max
\end{eqnarray*}$$

There are approximate solutions of this problem with an additional input—a demographic model with a fixed number of parameters, using various local search algorithms, but in practice, as mentioned above, these algorithms have proven to be ineffective. We present a new algorithm for the approximate solution of this more general problem using one of the most effective methods of global optimization—the GA.

### Representation of the demographic model and its structure

Assume a division of 1 ancestral population into 2 new isolated subpopulations. Then the number of population-splitting events directly depends on *P*, the number of considered populations. We represent the demographic model as a sequence of “time intervals” and population splits, each of which has a fixed number of parameters. Assume a fixed temporal order of the current observed populations: from an ancient ancestral population to the more recently formed subpopulations. This temporal order is usually known or can be imputed. If the number of populations is ≤3, then each split will divide the last formed population. Thus, a splitting event has only 1 parameter—the fraction of the population, which separates to form a new subpopulation.

The next important component of the demographic model is the concept of the time interval. First, we define this as a segment of time during which a certain dynamic of change of effective size is maintained for each population. We consider 3 main demographic dynamics of population growth: sudden, linear, and exponential change of the effective population size (Fig. S1). Sudden change is very popular for its simplicity, but exponential change is a commonly used model for population growth as well. We include linear change because it is tradeoff between sudden and exponential change and is more realistic than sudden change. Second, the parameters of migration rates between populations are constant during a given time interval. Thus, each time interval has the following parameters:

- time,
- effective population sizes at the end of the time interval,
- demographic dynamics of effective population size change,
- migration between populations, if there is >1 population.

The sizes of the populations at the beginning of any time interval are equal to the sizes of the populations at the end of the previous interval. The first time interval is a special one: we consider that it lasts from the beginning of the existence of the species and assume a demographic dynamic of sudden change for the effective population size of the ancestral population [11]. Therefore, in this interval the only parameter estimated is the size of the ancestral population. Note that the number of splitting events is determined by the number of populations under consideration, but the number of intervals can be varied and thus change the number of parameters of the demographic model, its detail, and complexity.

**Figure 3:**
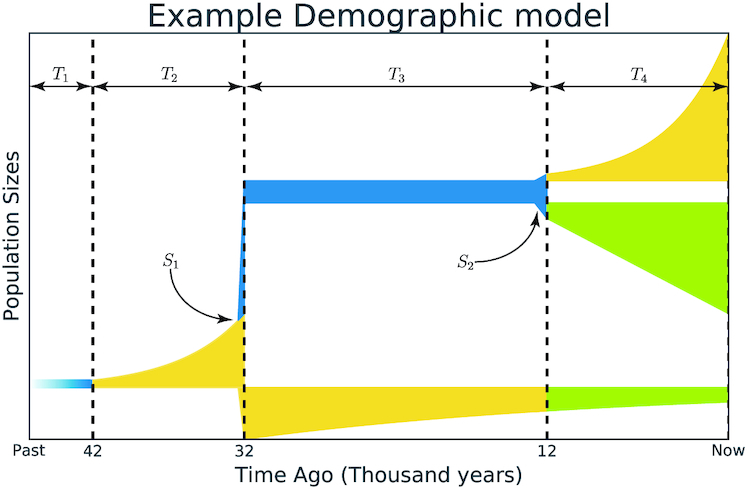
Example of a demographic model with a (2, 1, 1) structure. Time is shown along the x-axis and population size on the y-axis. Four time intervals are shown: *T*_1_, *T*_2_, *T*_3_, and *T*_4_, and 2 populations splits: *S*_1_ and *S*_2_. The structure of this model is (2, 1, 1) because *T*_1_ and *T*_2_ are time intervals for 1 ancestral population, followed by split *S*_1_, *T*_3_ is the time interval for 2 populations, and *T*_4_ is the time interval for 3 final populations after the second split *S*_2_. Time interval *T*_2_ has the following parameters: time of this interval, size of the ancestral population at the end, and the dynamic of size change. Time intervals *T*_3_ and *T*_4_ for each population will contain the same parameters plus migrations between populations. Split events *S*_1_ and *S*_2_ have fraction of size split as parameter. The first interval contains the size of an ancestral population, but it can be ignored because it could be implicitly evaluated from other parameters [11].

We now can define the concept of the "structure of the demographic model." In the case of an ancestral population splitting into 2 subpopulations, the structure of the model will include a number of time intervals that occur before and after a single splitting event. In the case of 3 populations, the structure includes a number of time intervals prior to the first split, those between the first and second split, and the ones after the second split. For example, assume we observe 3 populations. At the beginning, there was an ancestral population (*P_A_*) and this population started to grow in effective size. Then a split occurred that divided this ancestral population into 2 daughter populations (*P*_1_ and *P*_2_) that changed in effective size during 1 interval, followed by a split of the second population (into *P*_2*a*_ and *P*_2*b*_), resulting in 3 descendant populations that changed within 1 subsequent time interval. The structure of such a model would be described as (2, 1, 1) (Fig. 3). The simplest model structures would be for 2 populations—(1, 1), and for 3 populations—(1, 1, 1).

More formally, the structure of the model is a sequence of the form $S^* = \lbrace s^*_i\rbrace _{i=1}^P, \quad s^*_i \in \mathbb {N}$, where *P* ∈ {2, 3} is the number of populations. In this case, the number of parameters $N_\Theta (S^*)$ of the demographic model with the structure S* will be determined as follows:

$$\begin{eqnarray*}
N_\Theta (S^*) = (P-1) + \sum\nolimits_{i = 0}^P{N_\Theta ^i(s^*_i)},\quad \\
\text{where} \quad N_\Theta ^i(s^*_i) = \left\lbrace \begin{array}{@{}l@{\quad }l@{}}3(s^*_1 - 1), & \text{if } i = 1,\\
7s^*_2, & \text{if } i = 2,\\
13s^*_3, & \text{if } i = 3. \end{array}\right.
\end{eqnarray*}$$

The term (*P* − 1) corresponds to the number of split parameters, and $\sum _{i = 0}^P{N_\theta ^i(s^*_i)}$ is the number of time interval parameters. This number of parameters is valid for the GA that is described below, but the number of final model parameters is different. During the local search, which occurs after the GA, the dynamics of population size change are fixed and the final number of parameters $\overline{N}_\Theta (S^*)$ is:

$$\begin{eqnarray*}
\overline{N}_\Theta (S^*) = (P-1) + \sum\nolimits_{i = 0}^P{\overline{N}_\theta ^i(s^*_i)},\quad \\
\text{where} \quad \overline{N}_\Theta ^i(s^*_i) = \left\lbrace \begin{array}{@{}l@{\quad }l@{}}2(s^*_1 - 1), & \text{if } i = 1,\\
5s^*_2, & \text{if } i = 2,\\
10s^*_3, & \text{if } i = 3. \end{array}\right.
\end{eqnarray*}$$

Thus, we can unambiguously interpret the demographic model according to the list of parameters and its structure by fixing for each time interval a certain order of parameters.

### General approach

The general algorithm is a series of executions of the GA (Fig. S2). Suppose we have the initial and final structures that define the initial and final number of parameters (definition of the model structure is presented in the previous section) of the demographic models, derived from considerations about the populations we are trying to model. During the GA, the structure of the model does not change; i.e., the model’s parameters for the current structure are optimized. This restriction makes the procedure of crossover in the GA simpler. As soon as the GA stops, the parameters of the best-fitting model are adjusted to improve the likelihood with a local search algorithm. If the structure of the model is not quite complex enough, i.e., some value in the model structure (e.g., model structure (1, 1, 1)) is less than the corresponding value in the final model structure (e.g., model structure (2, 1, 1)), its complexity (in terms of parameters) is increased and the GA is run again for the new model structure. The GA and local search are executed until the best-fitting parameters of the model with the final model structure are obtained.

#### Akaike information criterion

With an increase in the number of model parameters, we risk overfitting the model. A model with a large number of parameters will be better able to find parameter values corresponding to the observed data than a model with a smaller number of parameters, but at the same time it will correspond less to reality, e.g., due to data errors. The Akaike information criterion (AIC) [25] is commonly used to compare models with different numbers of parameters:

$$\begin{equation*}
\mathrm{AIC}(M(\theta ), S) = 2 \cdot k - 2 \cdot \log (\mathcal {L}(M(\theta )|S)),
\end{equation*}$$

where *k* is number of parameters of the model and $\log (\mathcal {L}(M(\theta )|S))$ is the value of the log-likelihood function.

The composite likelihood Akaike information criterion (CLAIC) is a modification of the AIC for composite likelihoods, which is important to implement when single-nucleotide polymorphisms (SNPs) that are used to build the AFS are linked [26,27]. The CLAIC is defined as follows:

$$\begin{equation*}
\mathrm{CLAIC}(M(\theta ), S) = 2 \cdot \mathrm{tr}(J(\theta ) H^{-1}(\theta )) - 2 \cdot \log (\mathcal {L}(M(\theta )|S)),
\end{equation*}$$

where *J*(θ) and *H*(θ) are the variability and Hessian matrices, respectively:

$$\begin{equation*}
J(\theta )=E_\theta \left\lbrace \frac{\partial \mathcal {L}(\theta |S)}{\partial \theta } \left( \frac{\partial \mathcal {L}(\theta |S)}{\partial \theta }\right)^T \right\rbrace,
\end{equation*}$$

$$\begin{equation*}
H(\theta ) = E_\theta \left\lbrace -\frac{\partial ^2 }{\partial \theta \partial \theta ^T} \mathcal {L}(\theta |S)\right\rbrace.
\end{equation*}$$

The smaller the AIC or CLAIC score is, the better the model fits the observed data. In practice, calculation of the CLAIC is very challenging. Coffman et al. [26] applied bootstrapping to adjust composite likelihoods during statistical inference of demographic history using the programs ∂a∂i and TRACTS [28] and thereby calculate the CLAIC. This implementation was included in GADMA. Therefore, to obtain an accurate CLAIC score, one should perform block bootstrapping over unlinked regions of the genome.

Obviously, for AIC it is enough to compare only the final models for each model structure after the local search because the number of parameters between the increases in model structure does not change, and therefore the value of the AIC score depends only on the likelihood values. In the implementation of GADMA, if the models with the best likelihood and best AIC or CLAIC (depending on whether SNPs are linked or not) score do not match, the user is informed about the overfitting.

There are other methods for determining the selection of the best demographic model given an AFS dataset, e.g., likelihood ratio tests, which were introduced by Coffman et al. [26]. However, it is not possible to use them, because they assume nested demographic models, which is incorrect in our case because the dynamics of population change can vary during the GA.

### Genetic algorithm

The GA is one of the most effective heuristic algorithms [21]. It is based on the principles of evolution, where the aim of the algorithm is to find an approximate solution to a problem that has the maximum or minimum value of the fitness function. At the beginning of the algorithm, a fixed-sized set of random solutions, called individuals, is created. The set itself is called a generation. Each individual is assigned a value of fitness, which is determined by the value of the fitness function. After this, new generations are iteratively produced with the help of mutations, crossover, and selection of the fittest individuals (i.e., the models with the highest likelihood scores). All these operations can be either deterministic or random, and their order can vary in different implementations. In our case, individuals are demographic models of the same structure, and the fitness function is the log-likelihood, $\log (\mathcal {L} (M | S))$, as described in Materials and Methods.

**Figure 4:**
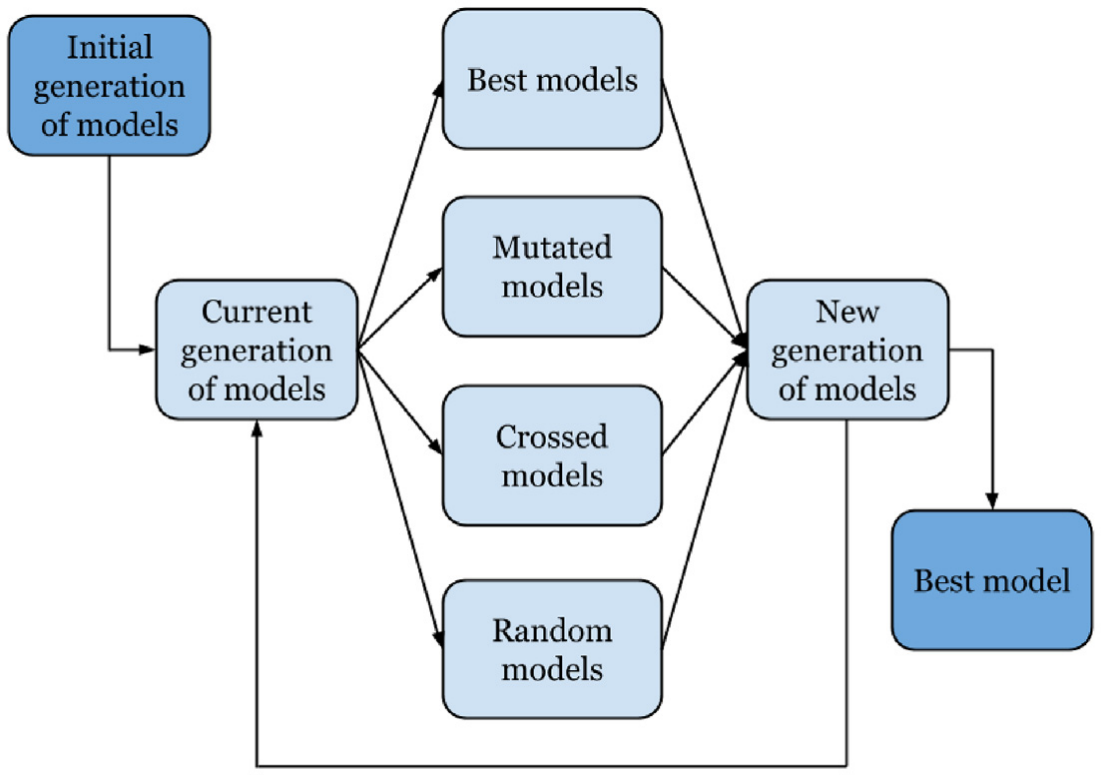
Diagram showing the scheme of the genetic algorithm used in GADMA.

In the first step of GADMA, a set of demographic models are randomly generated, if they have not been already specified. To form a new generation of demographic models, we select the most adapted models among a set of mutated, crossed, and random models in the previous generation. The value of the fitness function is used to select the most adapted models. The choice of models to be mutated or have crossover is random, but the probability of choice is directly proportional to the value of fitness: the better the fitness of the model is, the more likely it is to be selected. The GA stops when it can no longer obtain a better demographic model by the value of the fitness function for several iterations (Fig. 4).

#### Mutation of the demographic model

**Figure 5:**
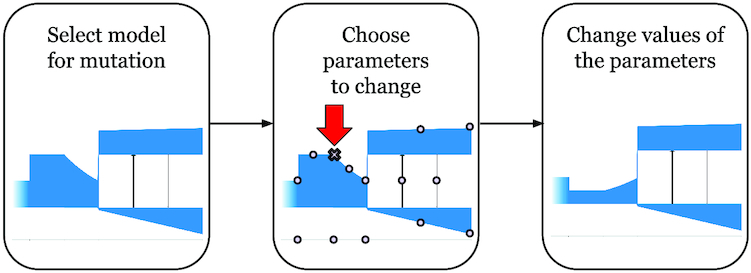
Diagram showing how mutation is used to change parameters of the demographic model in GADMA. In this example, ancestral population size is mutated at the second time interval prior to a population-splitting event.

The mutation of the demographic model (Fig. 5) is equal to the process of changing the values of several parameters. There are 2 constants associated with mutation of the model: the rate and the strength of mutation. The number of parameters to be mutated is sampled from a binomial distribution with a mean equal to the mutation strength. Parameters that are mutated are chosen with the probability that is directly proportional to their weights, which at the beginning are equal (i.e., the choice is equally probable), and then each weight can be increased when a mutation of the corresponding parameter has occurred, which leads to an improvement in the model. The measure of how much the value of each parameter is mutated is determined by the sign (+1 or −1, which are equally probable) and the rate of the mutation that is randomly sampled from the normal distribution, with the mean equal to the mutation rate and a variance equal to half of the mean. Among the parameters that can be mutated during estimation of the demographic model is the mode of population size change (sudden, growth, and exponential). If this parameter is chosen to be mutated, then the value (mode) will change to 1 of the other 2 population size change dynamics with equal probability.

#### Adaptive mutation rate and mutation strength

In the initial iterations of the GA, strong mutations of a large number of parameters are much more effective than weaker mutations of a small number of parameters, whereas when approaching the optimal solution the opposite is true. Therefore, the rate and strength of mutation can be adaptive; i.e., it changes during the operation of the algorithm. There are several ways to make an adaptive value, one of the most popular being the one-fifth algorithm [29]. First, we apply it to the mutation rate: at each iteration, if we have a “successful” solution, i.e., it becomes better after mutation, then we multiply the mutation rate by the constant *C* ∈ [1, 2]. If the solution is not “successful,” then we divide by a fourth-degree root of *C*, decreasing the mutation rate. In the case of the mutation strength for the “successful” solution, it is necessary to additionally check whether the decision has become the best solution during the entire course of the algorithm’s run.

Thus, often getting a new best solution with a mutation that occurs at the beginning of the GA, we increase the number of parameters that are changed during the mutation operation and the degree to which they are changed. As we approach the optimum solution and decrease in frequency, the number of parameters will also decrease and lead to a more accurate search. An increase in the number of parameters being mutated leads to a more efficient crossover. At the same time, the mutation rate is changed more frequently than the mutation strength, which makes the mutation procedure more effective.

#### Crossover of 2 demographic models

**Figure 6:**
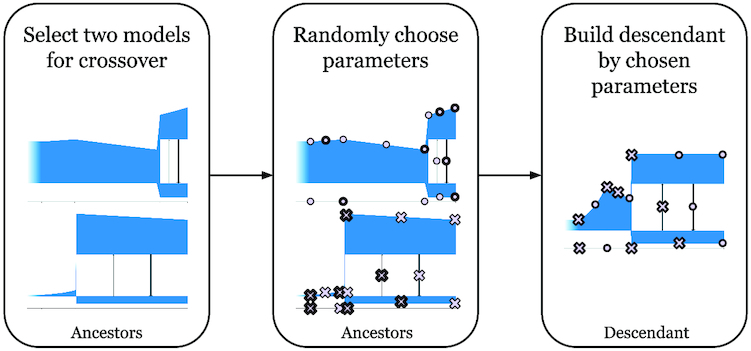
Diagram showing how crossover of 2 demographic models is performed in GADMA.

In order to have crossover of 2 demographic models, the models are represented as sequences of parameters. Each parameter of their descendant is chosen randomly with equal probability from one or the second parent (Fig. 6). Because the structure of models does not change during the operation of the GA, the number of parameters for all models will be the same. Consequently, the parameters can be unambiguously interpreted and easily crossed according to these criteria.

### Local search algorithms

Local optimizations are effective in cases when the initial solution is close to optimal. They are more accurate in adjusting parameters than the GA and can significantly improve its result. ∂a∂i and moments provide the following choice of algorithms for local optimization:

- The BFGS algorithm.
- L-BFGS-B—a modification of the BFGS algorithm, which is more efficient when the optimal parameters are close to the bounds.
- The Nelder-Mead method or simplex method.
- The Powell method.

The first 2 methods are gradient descent optimization algorithms [30], and the last 2 do not use a gradient. The Powell method was proposed by the authors of moments and was noted as the most effective, so it was adapted for use with ∂a∂i, and for our experimental studies (see below) it was chosen as the local search algorithm.

### Increasing the complexity of the structure of the demographic models

We need to be able to increase the complexity of the structure of the demographic model in order to find an optimal solution. To do this, a time interval is selected and then divided into 2 equal intervals (i.e., the division based on the median). The time interval is chosen randomly on the basis that the new structure S* should not become greater than the final *S*^*F*^ according to 1 of its values: $s^*_k \le s^F_K,\quad \forall k \in [1,P]$. The values of the parameters of the newly formed time intervals are calculated for the parent: the size of the population of the first time interval is equal to the size in the middle of the parental time interval, and the parameters of the second time interval are equal to the population size at the end, with the time of both intervals equal to half of the parental time, while migration between populations and the demographic dynamics of population size change remain the same. In essence, the demographic model has more parameters after its structure is increased; however, the history and likelihood remain the same.

## Results

We implemented the method described above in the program GADMA, written in the Python programming language. We explored the efficiency of our method using simulated data and several previously published datasets. It is important to note that tests using simulated AFS data should be interpreted with caution; these analyses and their associated demographic models are simplified for the sake of computational efficiency, and additional tests must be performed. For previously published datasets, GADMA was used to first infer demographic models for 2 and 3 modern human populations using the dataset analyzed by Gutenkunst et al. [11]. For these analyses, different parameter values within GADMA were examined, including the initial structure of the demographic models and using either ∂a∂i or moments to infer the optimal model of demographic history. The case of 2 human populations when initial structure and the final structure are not equal corresponds to the use of the increase of the model complexity feature that improves the result by finding simple models first and detailing them later. We then inferred demographic models for the history of 2 populations of the Gillette’s checkerspot butterfly, *Euphydryas gillettii*, and these were compared to the previous models reported by McCoy et al. [31]. Last, we used GADMA to reconstruct the demographic history of the Gaboon forest frog, *Scotobleps gabonicus*, which occurs in central and western African rainforests, based on a dataset generated and originally reported by Portik et al. [32].

### Tests on the simulated AFS data

In order to demonstrate that GADMA is computationally and statistically efficient, 3 datasets were simulated with moments using the following sudden population size dynamics (all parameter values are presented in Tables S6–S8):

- Bottleneck model for 1 population (4 parameters),
- Simple ancestral population division with asymmetric migration between 2 descendant populations (5 parameters),
- Secondary contact with symmetrical migration for 3 populations following split of 1 of the 2 descendant populations (8 parameters).

All simulated AFS were unfolded with a size of 20 chromosomes per population. The Powell method was chosen as local search algorithm. All runs were repeated 50 times for 1 and 2 populations and 10 times for 3 populations.

GADMA was compared with 2 methods: (i) local searches starting from different initial values and (ii) using the ∂a∂i pipeline, which was readjusted for moments use. The number of initial parameters for the first method and the number of replicates in the ∂a∂i pipeline were selected so that the mean number of fitness function evaluations was almost the same as in GADMA. For example, in case of 1 population: 40 initial points for the local search and 5 rounds of 10, 10, 10, 10, and 20 replicates for the ∂a∂i pipeline. For the comparison, GADMA was launched for the same demographic models as the local search approaches. But 2 additional GADMA variations were included: 1 with all parameters, but with fixed dynamics of population sizes (sudden), and a second with variation of these dynamics. Therefore, 5 different optimizations were compared and the results including parameter values, maximum, mean, and standard deviation of log-likelihood are provided in Tables S6–S8.

For 1 population, 50 runs of the ∂a∂i pipeline showed the best demographic model compared to the other methods. Although the best model obtained using GADMA had slightly worse likelihood score, GADMA was better on average. During demographic inferences without limitations on the population dynamics, the known problem about the uncertainties of the AFS appeared: the model with an early exponential bottleneck has almost the same likelihood as the model with sudden population size changes. All 3 versions of GADMA showed nearly identical results with the better mean and standard deviation of the likelihood score compared to local search and ∂a∂i pipeline.

For 2 populations, all methods were comparable in terms of the best model inferred after 50 replicate runs. All optimizations except the GADMA without limitation on size dynamics were comparable in terms of mean and variation of likelihood. The local search from different initial points and GADMA with prior knowledge about model were both able to infer the true model. Two additional simulations with GADMA also showed close to the optimum demographic models and the optimization without limitation on size dynamics inferred sudden changes as expected.

For 3 populations GADMA with presized model received the best maximum, mean, and variation of likelihood among all observed methods. Both additional optimizations without prior demographic model knowledge showed symmetric migrations and all extra parameters close to true values. GADMA without limitation on size change dynamics inferred non-zero migration after split of ancestral population; however, we assume that this could be because not enough runs were performed. All inferred dynamics—in spite of the fact that not all of them are sudden—showed the constancy of size change during time intervals. Thus we could argue that GADMA could infer the true demographic history without any knowledge of its model.

### Testing the human Out of Africa model with GADMA

One of the most popular demographic history models for human populations is the so-called “Out of Africa” model, which consists of 3 populations [11,12,33,34]:

- YRI — Yoruba individuals from Ibadan,
- CEU — Utah residents with ancestry from northern and western Europe,
- CHB — Han Chinese individuals from Beijing.

**Figure 7:**
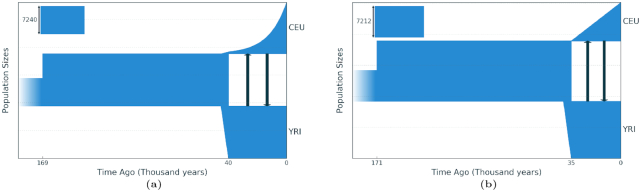
(a) Demographic model of 2 human populations (YRI and CEU) inferred using ∂a∂i, as originally reported by Gutenkunst et al. [11]. This model has 6 parameters. The size of the African population after its growth 169,000 years ago is constant and migration rates between African and European populations are symmetric. (b) The demographic model from the same allele frequency spectrum inferred using GADMA with 12 parameters. Note that the differences between the inferred models are the slightly later age of the split between the YRI and CEU populations and the linear population growth (as opposed to exponential growth) in the CEU population in the model obtained with GADMA.

**Figure 8:**
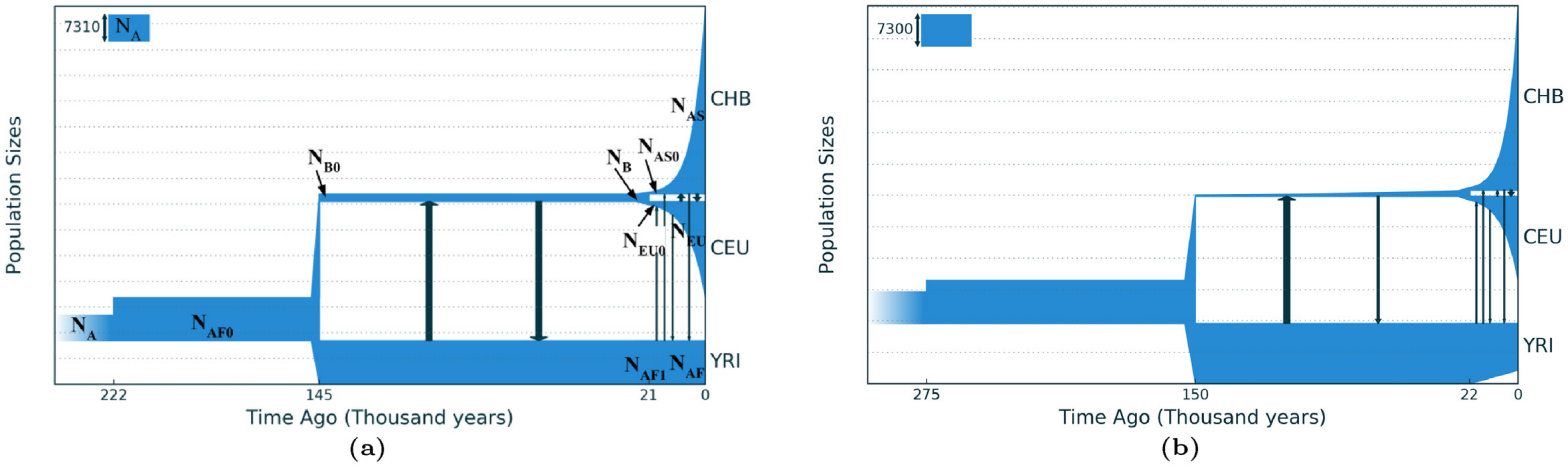
(a) Demographic model of 3 human populations (YRI, CEU, CHB) inferred using ∂a∂i, as originally reported by Gutenkunst et al. [11], which included 13 parameters. (b) The demographic model from the same allele frequency spectrum inferred using GADMA with 20 parameters.

To demonstrate the effectiveness of our method, we choose to use the AFS from Gutenkunst et al. [11], in which the ∂a∂i method is introduced and the demographic history models for 2 (YRI, CEU) and 3 (YRI, CEU, CHB) populations are inferred from this spectrum. These models (Fig. 7a and 8a) were obtained from a large number of local optimization launches and also have a number of restrictions on the number of population parameters: the size of the YRI population does not change after the first expansion of the ancestral population, migrations are symmetrical, and the dynamics of population size change are fixed as sudden, except for the last time interval for CEU and CHB, where exponential growth occurs. The model for 2 populations has a total of 6 parameters whereas the model for 3 populations has a total of 13.

The 21 × 21 × 21 AFS was constructed by Gutenkunst et al. [11] on the basis of the Environmental Genome Project [35]. All biallelic SNVs from non-coding regions of 219 genes (totaling 5.01 Mb) were considered and the effective length of the used sequence was equal to *L* = 4.04 × 10^6^. We used the same neutral mutation rate equal to μ = 2.35 × 10^−8^ per site per generation and the same generation time equal to *T*_g_ = 25 years as in Gutenkunst et al. [11]. Thus, the average frequency of mutation in 1 individual per generation is equal to θ_0_ = 4μ*L* = 0.37976.

The following parameters of the GA were used: the size of the generation of the demographic models was chosen to be equal to 10, the strength and mutation rate was equal to 0.2, and the proportions of the best, mutated, crossed, and random models in the new generation were 0.2: 0.3: 0.3: 0.2. The strength and rate of mutation were adaptive with the constants of 1.05 and 1.02, respectively. The AFS was simulated using ∂a∂i with a *G* = {40, 50, 60} grid size, the value of the likelihood was considered significant to the second decimal point, and the GA stopped after 100 iterations without improvement. As for the local optimization search, the Powell method was chosen.

For the human population data, we used the block bootstrapped dataset from Gutenkunst et al. [11], where it was performed over 219 sequenced loci under the assumption that the loci are well separated and can be treated as independent. The confidence intervals (CIs) reported in Tables 1 and 3 were calculated as $\overline{\theta ^*} \pm \sigma (\theta ^*)$, where θ* denotes the maximum likelihood values of parameters, and $\overline{\theta ^*}$ and σ(θ*), the mean and standard deviation of the bootstrap results. All our model parameters are positive by definition, so their logarithms were used to calculate CIs. In the case of 3 human populations, CIs are different to those from the original paper by Gutenkunst et al. [11]. However this fact popped out in our analysis and we argue that is because of the different optimization method: for each bootstrapped data point, only 1 local optimization with wider search intervals for parameter values was launched from the found optimum point.

**Table 1:** Maximum likelihood parameters for different demographic models for 2 human populations (YRI and CEU) inferred using either ∂a∂i or GADMA (the latter under 3 different parameter settings)

	Model from ∂a∂i example		Model from GADMA (1)		Model from GADMA (2)		Model from GADMA (3)	
No. of parameters	6		6		8		8	
Log-likelihood	−1,066.35		−1,066.28		−1,065.87		**−1,065.15**	
CLAIC (ϵ = 10^−5^)	35,059.26		33,702.64		**28,389.58**		53,101.50	
Population size (95% CI)								
* N_*A	7,240	(6,841−9,166)	7,230	(6,840−9,167)	7,200	(6,920−7,519)	7,210	(6,561−8,914)
* N*_AF0	13,620	(11,575−16,177)	13,580	(11,574−16,180)	13,400	(11,331−18,032)	14,000	(10,682−25,283)
* N*_EU0	515	(383−809)	530	(383−810)	560	(415−673)	25	(14−170)
* N*_EU	13,360*^e^*	(7,320−18,892)	12,400*^e^*	(7,314−18,906)	12,100*^e^*	(9,171−18,576)	8,950*^l^*	(5,985−10,623)
* N*_AF	(=*N*_AF0)		(=*N_*AF)		13,500	(11,698−14,834)	13,300	(10,221−15,611)
Migration rate per generation (95% CI)								
* m*_AF−EU(×10^−5^)	6.3	(4.5−9.6)	6.3	(4.5−9.6)	7.1	(1.1−1.9)	5.8	(4.0−11.3)
* m*_EU−AF(×10^−5^)	(=*m*_AF−EU)		(=*m*_AF−EU)		6.1	(9.5−14.8)	6.1	(4.1−9.7)
* T*_AF (kya)	168.5	(87.3−165.8)	171.5	(87.3−165.9)	176.5	(122.1−230.6)	171.1	(75.1−224.2)
* T*_AF−EU (kya)	40.0	(30.8−58.5)	40.8	(30.8−58.6)	42.3	(35.3−46.7)	34.8	(29.1−47.6)

^*e*^Exponential growth.

^*l*^Linear growth.

kya: thousand years;*N*_A : size of ancestral population; *N*_AF0: size of ancestral population after growth; *N*_EU0: size of European population after divergence of ancestral population; *N*_EU: current size of European population (after growth); *N*_AF: current size of African population; *m*_AF − EU: migration rate from European to African population; *m*_EU−AF: migration rate from African to European population; *T*_AF_:_ time of ancestral population size growth; *T*_AF−EU: time of divergence. Best log likeliood value and CLAIC score are marked in bold.

#### The YRI, CEU 2-population example

Three demographic models were inferred from the same AFS: 1 model using the same parameters as in Gutenkunst et al. [11] (model 1) and 2 with all possible 9 parameters (models 2, 3) but with different initial demographic model structures. Model 2 had a structure (1, 1), which then expanded to (2, 1) during the GADMA run, whereas model 3 had an initial structure of (2, 1). We ran GADMA 10 times for each of the 3 models (Table 1, Fig. 7). All 3 models resulted in likelihoods better than the final demographic model originally inferred in Gutenkunst et al. [11]. The parameters of models 1 and 2 are not significantly different, and model 3 has the best likelihood value and CLAIC score. Model 3 shows a lower population size of Europeans, a larger rate in their growth (from 25 individuals to 9,000), and a shorter separation time than the best model found in Gutenkunst et al. [11], as well as models 1 and 2. Migration rates between the populations were chosen to be asymmetric, but they are largely equal to each other and coincide with values among the 3 models.

To demonstrate the inefficiency of the methods of local optimization for the model from Gutenkunst et al. [11], 1 of the methods proposed by ∂a∂i, BFGS, was launched 100 times. In each run, the initial parameter values were chosen randomly. The best value of the log-likelihood was −1,629.24, which is quite far from the optimal value of −1,066.35 reported in Gutenkunst et al. [11]. The average time of 1 optimization run was ∼21 minutes. We then used a more efficient local optimization method implemented in the dadi-pipeline developed by Portik et al. [32]. It implements a scheme of sequential runs using the Nelder-Mead local optimization with initial random parameter values and perturbation of values between runs. We used the following settings of the dadi-pipeline tool: 3 rounds with 10, 20, 50 replications with 3-, 2-, and 1-fold perturbations, respectively, for each round. The dadi-pipeline was launched 50 times and the best resulting model had a log-likelihood equal to −1,073.98. The average run time for 1 launch of optimization was ∼10 minutes.

To compare the runs with different initial demographic model structures (models 2 and 3), characteristics such as time for 1 iteration of the GA, number of iterations, and mean and standard deviation of the log-likelihood value were calculated (Table 2). Launches with a simple initial model structure show a more stable result in terms of the likelihood value, but they have a longer average run time for 1 iteration. Furthermore, all the models obtained for simple-structure launches have the same demographic dynamics of population size change and similar parameters as the final model reported in Gutenkunst et al. [11], which is incorrect in cases involving launches of complex-structure models, as the best model shows a linear growth of the European (CEU) population as opposed to exponential growth in other cases. At the same time, although the launches of models with complex model structures result in a final model with a better log-likelihood score, it differs from the other models in terms of parameter values, which may indicate that it is inaccurate.

**Table 2:** Comparison of runs using GADMA with different initial demographic model structures: simple (10 times) and complex (10 times) for the case of searching for the optimum demographic models for 2 human populations, YRI and CEU

Model	Initial structure	Mean time per iteration (sec)	Mean No. of iterations	Mean ± SD log LL
2	(1, 1)	4.06	2,938	−1,066.39 ± 0.22
3	(2, 1)	3.77	1,400	−1071.16 ± 14.35

log LL: log likelihood.

#### The YRI, CEU, CHB 3-population example

We also applied GADMA to infer demographic models for the case of 3 human populations based on same the AFS used in Gutenkunst et al. [11]. The first model (model 1) used the same parameters as in Gutenkunst et al. [11], and their corresponding values were inferred (Table 3). GADMA yielded better log-likelihood values for parameters than those reported in Gutenkunst et al. [11]. However, the timing of the split between the YRI population and the CEU+CHB populations was dated to 400,000 years ago, which is 250,000 years older than that inferred in any previous studies. To correct this, we restricted the age of this splitting event to 150,000 years ago based on previously published estimates [36–38]. A demographic model (model 2) was inferred with this age restriction, which yielded a better log-likelihood value than that in Gutenkunst et al. [11]. Next, we inferred the demographic model (model 3) that included all 20 parameters. Here we also observed an unrealistic earlier age for the divergence between YRI and CEU+CHB (results not shown). When we applied the 150,000 year age constraint as in model 2, we inferred a demographic model (model 3) that not only showed the highest log-likelihood value, but also the best CLAIC value.

**Table 3:** Maximum likelihood parameters inferred from the demographic models for the YRI, CEU, and CHB human populations

	Model from Gutenkunst et al. [11]		Model from GADMA (1)		Model from GADMA (2)		Model from GADMA (3)	
No. of parameters	13		13		13		20	
Log-likelihood	−6,316.89		−6,314.40		−6,315.86		**−6,288.36**	
CLAIC (ϵ = 10^−4^)	43,865.22		45,182.80		46,161.50		**42,761.68**	
Population size (95% CI)								
* N*_A	7,300	(6,065−8,185)	6,000	(4,841−11,781)	7,300	(6,084−11,227)	7,300	(6,713−10,696)
* N*_AF0	12,300	(10,177−13,668)	11,840	(10,126−13,750)	12,200	(10,152−13,862)	9,900	(3,837−17,433)
* N*_B0	2,100	(1,667−2,299)	2,050	(1,651−2,276)	2,070	(1,652−2,308)	280	(16−528)
* N*_B	(=*N*_B0)		(=*N*_B0)		(=*N*_B0)		1,450*^e^*	(1,127−1,798)
* N*_AF1	(=*N*_AF0)		(=*N*_AF0)		(=*N*_AF0)		14,000	(10,812−18,451)
* N*_AF	(=*N*_AF0)		(=*N*_AF0)		(=*N*_AF0)		11,000*^l^*	(4,629−20,567)
* N*_EU0	938	(598−1,496)	930	(556−1,686)	950	(543−1,721)	890	(613−1,164)
* N*_EU	27,300*^e^*	(16,844−38,704)	24,700*^e^*	(12,232−48,543)	23,700*^e^*	(12,310−48,914)	19,600*^e^*	(13,745−35,578)
* N*_AS0	510	(326−788)	500	(302−876)	510	(299−867)	560	(361−877)
* N*_AS	53,200*^e^*	(26,001−91,501)	50,000*^e^*	(14,211−159,816)	46,200*^e^*	(14,205−167,491)	42,200*^e^*	(18,188−100,754)
Migration rate per generation (95% CI)								
* m*_AF−B(×10^−5^)	25.0	(22.3−33.8)	26.2	(22.7−33.9)	25.4	(20.9−35.4)	17.0	(11.8−29.3)
* m_*B−AF(×10^−5^)	(=*m*_AF−B)		(=*m*_AF−B)		(=*m*_AF−B)		57.4	(44.0−74.4)
* m*_AF−EU(×10^−5^)	3.0	(1.47−5.7)	3.1	(1.45−5.84)	3.1	(1.58−5.52)	4.5	(1.98−8.26)
* m*_EU−AF(×10^−5^)	(=*m*_AF−EU)		(=*m*_AF−EU)		(=*m*_AF−EU)		2.8	(1.34−5.91)
* m*_AF−AS(×10^−5^)	1.9	(0.02−77.3)	1.9	(0.11−20.5)	2.0	(0.35−8.37)	0.9	(0.01−16.2)
* m*_AS−AF(×10^−5^)	(=*m*_AF−AS)		(=*m*_AF−AS)		(=*m*_AF−AS)		2.1	(0.58−6.35)
* m*_EU−AS(×10^−5^)	9.6	(0.39−164.6)	10.2	(3.43−25.9)	10.2	(2.60−31.2)	18.8	(11.5−29.7)
* m*_AS−EU(×10^−5^)	(=*m*_AS−EU)		(=*m*_AS−EU)		(=*m*_AS−EU)		6.8	(1.09−28.35)
* T*_AF (kya)	220.8	(249.4−5,201.3)	570	(297.6−2,827.9)	232	(193.3−2,211.2)	274.8	(198.2−804.7)
* T*_B (kya)	144.0	(246.4−2,503.7)	400	(234.2−2,309.9)	150	(152.1−2,218.3)	149.8	(98.9−666.9)
* T*_EU−AS (kya)	21.1	(17.6−25.1)	21.0	(17.2−26.4)	21.1	(17.1−26.4)	22.4	(18.2−26.9)

^*e*^Exponential growth.

^*l*^Linear growth.

kya: thousand years; *N*_A: size of ancestral population; *N*_AF0: size of ancestral population after growth; *N*_B0: size of CEU+CHB population after divergence of ancestral population; *N*_B: size of CEU+CHB population before its split; *N_*AF1: size of YRI population at the time of CEU+CHB population divergence; *N*_AF: current size of YRI population; *N*_EU0: size of CEU population after divergence; *N*_EU: current size of CEU population; *N_*AS0: size of CHB population after divergence; *N*_AS: current size of CHB population; *m_*1−2: migration rate from population 2 to population 1; *T*_AF: time of growth of size of ancestral population; *T_*B: time of divergence of ancestral population to YRI population and CEU+CHB population; *T*_EU−AS: time of divergence of CEU+CHB population to CEU population and CHB population. Best log likeliood value and CLAIC score are marked in bold.

As in the case of the 2 human population example, we also tested the 3-population case with the BFGS local optimization used in ∂a∂i. We launched the optimization 100 times from randomly selected initial parameters, and the best log-likelihood value obtained was −6,323.99, which slightly differs from the optimal log-likelihood −6,316.89, which is much less than that from the comparison for 2 populations.

With the exception of the earlier age of divergence between the YRI and CEU+CHB populations in model 1, demographic models 1 and 2 and the one inferred by Gutenkunst et al. [11] have similar log-likelihood values and parameter estimates. In model 3, which has 20 parameters and the best CLAIC value, some parameters are also quite similar to the values in the other 2 models. The major exceptions, however, are the inferred migration rates and population size of the Eurasian population, which exponentially grows from 200 to 1,500 individuals after the split of the ancestral population. For comparison, in other models this number is a constant equal to 2,000 individuals, which seems less realistic than exponential growth. Migration rates vary considerably: they are higher in model 3 compared to models 1 and 2 or the one found by Gutenkunst et al. [11]. Model 3 shows that the largest migration occurred between the YRI and CEU populations, and following the ancestral division, between the CEU and CHB populations. Moreover, the more geographically distant the populations are, the smaller is the observed migration rate.

GADMA was launched 10 times for each of the 3 models using ∂a∂i, and the best solutions were observed (Table 1, Figure 8). We also launched GADMA using moments 10 times to compare its effectiveness with ∂a∂i. The authors of moments conducted similar comparisons on simulated data [12]. Various characteristics of run time and stability of the log-likelihood value based on the results of 20 GADMA runs (10 using ∂a∂i, 10 using moments) are presented in Table 4. Log-likelihood values of models inferred using moments were recalculated using ∂a∂i with the *G* = {40, 50, 60} grid size simulated AFS so that log-likelihoods obtained with the 2 methods were comparable. Analyses using moments were 7.5 times faster than ∂a∂i, whereas those using ∂a∂i were more accurate: the average and variance of the likelihood values of the inferred models were better than the values inferred using moments.

**Table 4:** Comparison of outputs from different GADMA runs using either ∂a∂i or moments for inferring demographic models for the YRI, CEU, CHB human populations with 20 parameters.

	Mean time per iteration (sec)	Mean No. of iterations	Mean ± SD log LL
∂a∂i	136.81	5,531	−6,293.56 ± 7.26
moments	18.44	4,700	−6,305.21 ± 12.72

For the runs performed with ∂a∂i, the log-likelihood (log LL) value was calculated using a *G* = {40, 50, 60} grid size.

#### Estimation of the *CLAIC* for human population data

To compare demographic models of the human dataset with different numbers of parameters, we estimated the CLAIC scores for each model. As the implementation from Coffman et al. [26] uses numerical methods for Hessian and gradient estimations, the gradient descent depends on the step size, denoted by epsilon (ϵ). Very small values of ϵ causes numerical issues, and the analysis should be done with different ϵ values to ensure that the results are stable (R. Gutenkunst, personal communication). We performed experiments to show how the value of CLAIC depends on the value of ϵ and on the choice of using either ∂a∂i or moments. The results of CLAIC calculation for all models (8 models for modern human data and 15 models for Gillette’s checkerspot butterfly population; see below) are presented in Table S5. The value of ϵ was taken to equal to 1 of 7 values: 10^−8^, 10^−7^, ... up to 10^−2^. Using ∂a∂i, the most stable result was for ϵ values between 10^−5^ and 10^−3^ (for all models). The interval over which models were stable using moments was wider: [10^−6^, 10^−2^]. Values 10^−8^, 10^−7^ do turn out to cause numerical issues resulting in the CLAIC to be ∼2 · logLL due to the small value of trace. Thus, moments showed very stable and reliable results for all values of ϵ except for those with small values. For ∂a∂i the results were not as reliable, and we recommend checking the CLAIC for different values of ϵ when ∂a∂i is used.

### Demographic history of Gillette’s checkerspot butterfly

We next tested GADMA using data from McCoy et al. [31], who examined the demographic history of Gillette’s checkerspot butterfly (*Euphydryas gillettii*). We used the same AFS as that used in the original article, which consisted of 8 individuals from a population in Colorado (CO) and 8 individuals from the native population in Wyoming (WY). For our analyses, we used 2 AFSs of size 13 × 13, 1 for synonymous SNVs only and 1 including all SNVs.

McCoy et al. [31] tested 3 types of demographic models: (i) type A—models without migration between populations, (ii) type B1—models with unidirectional migration from CO to WY, and (iii) type B2—models with bidirectional migration between CO and WY. In the original article, the 3 demographic models were tested using the AFS based on synonymous SNVs only. Model A had the best CLAIC value, so the type A demographic model was inferred from the AFS using all SNVs. We used models A and B2 to infer the demographic models from both AFSs (synonymous SNVs and all SNVs) with GADMA.

Without considering migration, the models used by McCoy et al. [31] had the following structure: there was 1 population of *N*_A size, which at some point in time divided into 2 subpopulations, the size of which did not change further (sudden change of population size). All parameters were calculated with respect to *N*_A and had the following notation: η_WY, η_CO — relative population sizes at the current time; τ_SPLIT — time/age of the population-splitting event; and *M*_WY−CO, *M_*CO−WY — scaled migration rates. For the models we inferred using GADMA, we also included the parameter η_WY0 ∈ [0, 1] — the size of the WY population immediately after the splitting event or the fraction of the size of the ancestral population that forms the WY population. The size of CO population immediately following the splitting event is equal to 1 − η_WY0, because *N*_A = 1 before the ancestral population splits. However, in the case when the population size change is sudden, the size of the populations following the splitting event is equal to the size at the present time.

We ran 4 executions of GADMA with different data inputs: (i) the AFS using synonymous SNVs only without migration, (ii) the AFS using synonymous SNVs only with migration, (iii) the AFS using all SNVs without migration, and (iv) the AFS using all SNVs with migration. For each execution, the analysis was repeated 50 times. moments was used to simulate the AFS owing to its faster computational speed. However, of the final log-likelihood scores that are presented in Table S1, 5 were calculated using ∂a∂i with a *G* = {32, 42, 52} grid size in order to compare our results with the original findings reported by McCoy et al. [31].

**Table 5:** Demographic models and their associated parameters inferred in GADMA using an allele frequency spectrum based on all SNVs for Gillette’s checkerspot butterfly populations

	Model A McCoy et al. [31]		Model A		Model B2 (1)		Model B2 (2)		Model B2 (3)	
log LL	−283.53		−277.82		**−267.49**		−278.57		−282.74	
CLAIC (ϵ = 10^−4^)	1,620.85		1,619.03		**−354.00**		−185.48		51.85	
Population size (95% CI)										
η_WY0	NA		0.584	(0.359−0.738)	0.028	(0.0−0.254)	0.566	(0.463−0.660)	(=η*_WY*)	
η_WY	1.320	(1.085−1.594)	1.358	(1.152−1.650)	1.225	(1.052−1.494)	1.773*^l^*	(1.383−2.379)	1.261	(1.053−1.559)
η_CO	0.173	(0.151−0.209)	0.089*^l^*	(0.058−0.127)	0.074*^l^*	(0.050−0.105)	0.105*^l^*	(0.078−0.144)	0.183	(0.162−0.217)
Migration rate per generation (95% CI)										
* m*_WY−CO	NA		NA		1.244	(0.515−1.637)	0.801	(0.170−1.231)	0.316	(0.0−0.677)
*m*_CO−WY	NA		NA		0.171	(0.0−0.362)	0.056	(0.0−0.266)	0.091	(0.0−0.273)
τ_SPLIT	0.117	(0.103−0.141)	0.138	(0.120−0.172)	0.259	(0.190−0.312)	0.162	(0.129−0.206)	0.129	(0.108−0.162)
Time of split in generations (95% CI)										
*t*_SPLIT	45	(44−48)	37	(28−45)	33	(28−41)	40	(35−49)	47	(44−54)

^*l*^Linear growth.

log LL: log-likelihood; η_WY0: relative (to *N*_A) size of WY population after divergence of ancestral population; η_WY: current relative (to *N_*A) size of WY population; η_CO: current relative (to *N*_A) size of CO population; *m_*WY−CO: scaled (by 2*N_*A) migration rate from CO population to WY population; *m_*CO−WY: scaled (by 2*N_*A) migration rate from WY population to CO population; NA: not applicable; τ_SPLIT: scaled (by 1/(2*N_*A)) time of divergence of ancestral population. Best log likeliood value and CLAIC score are marked in bold.

The length of 1 generation of the demographic models was selected to be 10; the strength and mutation rate were set to 0.2, with constants 1.0 and 1.02, respectively; and the proportions of the best, mutated, crossed, and random models in the new generation equal to 0.2: 0.3: 0.3: 0.2. The likelihood was considered significant to 2 significant digits and the GA stopped after 100 iterations without improvement. As for the local search, the Powell method was chosen. Because the structure of the demographic model from McCoy et al. [31] corresponds to the simplest structure (1, 1), it was chosen as both the initial and final structure of the demographic model.

The CIs were calculated for all resulting models (Table S1 and Table 5) the same way as was done for the human population data. In the case of the demographic models for the Gillette’s checkerspot butterfly, all our parameters are positive. McCoy et al. [31] did not use logarithms to calculate CIls; they just assumed the lower bound of the intervals to be positive. However, when we used logarithms to calculate CIs, we found them to be extremely wide for migration between the 2 populations. Another problem we encountered was the performance associated with bootstrapping the data for the Gillette’s checkerspot butterfly. The data were derived from RNA sequencing data, in which different alleles are likely linked. Bootstraping should be performed over unlinked regions of the genome. McCoy et al. [31] provide the assembly of the transcriptome, so it became possible for us to perform bootstrapping over the contigs of the assembly. This was applied to generate CIs and CLAIC scores.

**Figure 9:**
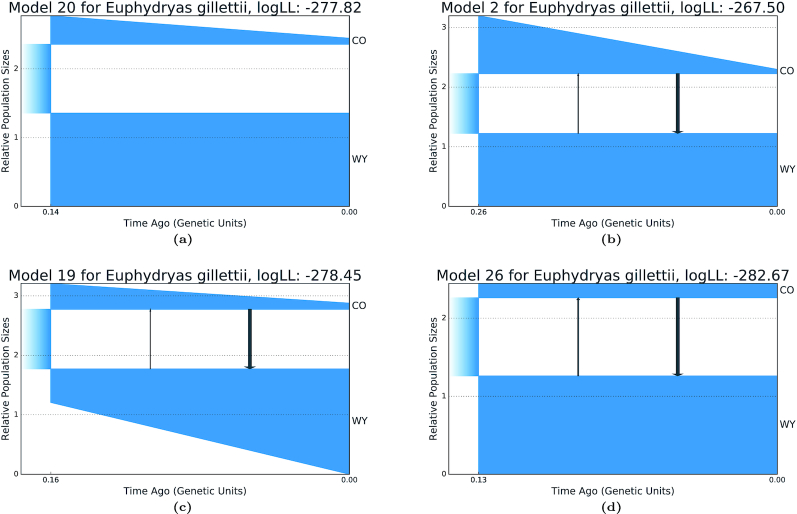
Alternative demographic models based on an AFS for Gillette’s checkerspot butterflies from Colorado (CO) and Wyoming (WY), inferred with GADMA. Models of type B2 have negligible migration rates. (a) Type A model: without migration; (b) Type B2 model with migration; (c) Type B2 model with migration; (d) Type B2 model with migration.

Several runs of GADMA produced different local minima, and the resulting alternative models and their inferred parameters are presented in Fig. 9 and Table 5, respectively. We note that 1 of the models selected by GADMA, Model 26 (Fig. 12d), was the same model as was inferred by McCoy et al. [31], which includes a sudden increase in population size following the splitting of the ancestral population. For the AFS-based synonymous SNVs, only models of type B2 had better likelihood values than models of type A, in contrast to the models inferred by McCoy et al. [31].

For all models, the CLAIC values were calculated and are presented in Table S5. The scores were calculated on the bootstrapped data to generate CIs by sampling over the contigs of the transcriptome assembly to avoid linkage (see above). For the AFS generated from synonymous SNPs, the model without migrations showed the best CLAIC score. However, the model with migration, which had the best likelihood, had the best CLAIC score for the AFS generated from all SNPs.

One of the findings reported by McCoy et al. [31] was that the demographic models that included migration may not be applicable when the real population history shows no evidence for migration. This conclusion was based on 2 factors: (i) the type A demographic model, inferred from the AFS using synonymous SNVs, had the best AIC score; and (ii) the estimate of the splitting time of the ancestral population in models that included migration had wide 95% CIs, such that the parameter boundaries included zero (see McCoy et al. [31]). However, during our regeneration of these authors’ results, we found errors associated with some of the parameters of the demographic models: the split time of the ancestral population and migrations were mixed up with each other, resulting in migrations, but not split times, having wide CIs that included zero. Moreover, our analyses using GADMA showed that the 95% Cl for the split time of the ancestral population is rather good (e.g., 0.189−0.323 for the case of the best B2 model) and that the migration rates are so small that they can be considered as zero. Therefore, the best-fitting model that includes migration suggests a demographic history without migrations. The demographic models inferred by GADMA also have negligible migration values, and demographic models of type B2 (those with bidirectional migration), inferred as the best-fitting models, had better CLAIC values than type A models.

The average population size of butterflies from CO was estimated as *N*_CO = 34 individuals by McCoy et al. [31]. If we scale the parameters of the best-fitting model so that the average size of the CO population is equal to this value (*x* · [η_CO + (1 − η_WY0)]/2 = *N*_CO), then for best model we get 33.6 generations ( *t*_SPLIT = 2 · *x* · τ_SPLIT) after the division of the ancestral population, which corresponds to the actual 33 generations observed (1977–2010). Such estimates were made for all resulting models (Table 5). However, the best-fitting model had the best estimated value of split time of the ancestral population (33.6 generations).

### Demographic history of the Gaboon forest frog

For our third evaluation of performance, we compared the demographic models inferred using GADMA with those inferred with the recently developed optimization method implemented in the dadi pipeline [32]. This pipeline uses ∂a∂i to simulate an AFS and infers parameters of the researcher-specified demographic model by several rounds of consistent runs using the Nelder-Mead local optimization method. During the first round, random parameter values are estimated, and during each successive round, the best parameters from the previous rounds are used. Prior to each Nelder-Mead local optimization, current parameter values are perturbed. Portik et al. [32] demonstrated the dadi pipeline using AFSs generated from the Gaboon forest frog (*Scotobleps gabonicus*), and these same data were used to perform the analyses using GADMA. Sampling included 84 samples from 33 localities of Lower Guinea, West Africa, which were divided into Northern and Southern populations according to hierarchical Bayesian clustering analysis of 7,633 unlinked SNPs generated using RADseq. Each Northern and Southern group was further divided into 3 clusters: Cameroon Volcanic Line North (CVLN), Cameroon Volcanic Line South (CVLS), and Cross River (CrossRiver) populations for the Northern group; and North Coast, South Coast, and East Gabon populations for the Southern group.

To perform our analyses using GADMA, 3 2Dl folded AFSs were chosen from Portik et al. [32]: (i) a 41 × 19 spectrum for Northern and Southern populations, (ii) a 31 × 19 spectrum for CVLN and CVLS, and (iii) a 15 × 31 spectrum for the CrossRiver and CVLN populations. In generating the AFSs, only a single SNP per RAD locus was kept, so loci are assumed to be independent. For each spectrum, we estimated best-fitting log-likelihood values, AIC scores, Akaike weights (ω_*i*_) [39], and parameters for 14 demographic models. Two demographic models assumed no divergence between populations, while the remaining 12 models assumed a split of the ancestral population and different assumptions related to migration rates, isolation, and population size changes. All population size changes were considered to have sudden change dynamics, which we included in our analyses as well. For all models, we provide inferred parameter values in Tables S2–S4, including the value of θ = 4*N*_A_μ*L*, where μ is the mutation rate per generation per site and *L* is effective sequence length.

The best-fitting demographic model inferred using the dadi pipeline for the Northern and Southern populations only suggests a population expansion, followed by secondary contact and symmetrical migration between populations (ΔAIC = 13.6, ω_*i*_ = 0.99). For the CVLN and CVLS populations, the best demographic model included population expansion, secondary contact, and asymmetrical gene flow from CVLN to CVLS (ΔAIC = 94.2, ω_*i*_ = 0.99). Finally, 2 demographic models explain the AFS data equally well for the CrossRiver and CVLN populations: 1 with secondary contact but no population size change and asymmetrical migration from CVLN to Cross River (ω_*i*_ = 0.53) and another that included an ancestral population division with continuous asymmetric gene flow (ω_*i*_ = 0.43).

We inferred all 12 demographic models with divergence as in Portik et al. [32] for each of the 3 AFSs with GADMA. Each of 36 runs was repeated 10 times as opposed to the 50 runs launched with the dadi pipeline. We originally had planned to conduct the analyses with ∂a∂i to simulate the AFSs with the same grid size as that used by Portik et al. [32]: *G* = {40, 50, 60} for the CrossRiver, CVLN, and CVLS populations; and *G* = {50, 60, 70} for the Northern and Southern populations. However, we found that the optimization with ∂a∂i was unstable and therefore, we set upper bounds on population size (12*N*_A) and generation time (5*N*_A). Under these parameters, ∂a∂i was found to be stable, but some parameters were equal to the upper bounds and in such cases GADMA was launched 10 times for each model using moments with larger upper bounds (100*N*_A for population size and 10*N*_A for generation time). The inferred demographic models and their estimated parameters are presented in Tables S2–S4. We report demographic models estimated with moments, and if a similar model was inferred by both ∂a∂i and moments, we only report the result with the best log-likelihood value.

For almost all 12 demographic scenarios, GADMA was able to infer better models in terms of both log-likelihood scores and parameters than were previously inferred using the dadi pipeline in Portik et al. [32]. Moreover, we obtained better alternative models for the CrossRiver, CVLN, Northern, and Southern populations. These models included an initial split of the ancestral population with ancestral asymmetric migration and a population size change (ΔAIC = 3.24, ω_*i*_ = 0.82) for the CrossRiver and CVLN populations, and secondary contact with asymmetric migration and population size change (ΔAIC = 6.82, ω_*i*_ = 0.97) for the Northern and Southern populations. For the third AFS of the CVLN and CVLS populations, the model that includes secondary contact with asymmetric migration and population size change was found to be superior on the basis of comparison of the AIC (ΔAIC = 87.98, ω_*i*_ > 0.99), similar to the findings by Portik et al., but received a higher log-likelihood value: −455.17 using GADMA versus −463.3 in Portik et al. [32].

**Figure 10:**
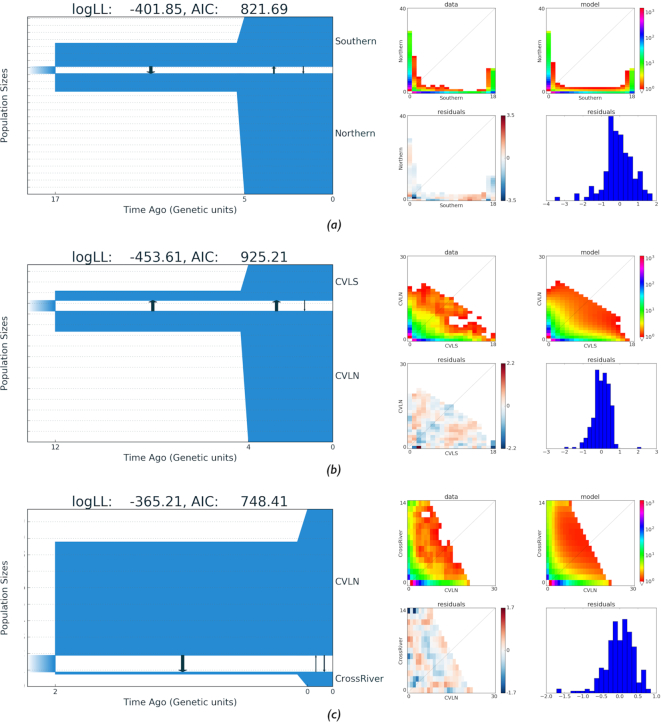
Demographic models with the highest log-likelihoods obtained from allele frequency spectra for different populations of the Gaboon forest frog, inferred using GADMA. (a) Demographic model inferred for the Northern and Southern populations; (b) demographic model inferred for the CVLN and CVLS populations; and (c) demographic model inferred for the CrossRiver and CVLN populations. The plots to the right of the demographic models show the AFS data (upper left), AFS of the demographic model (upper right), Anscombe residuals between model and data (lower left), and the histogram of the Anscombe residuals (lower right).

We next inferred the demographic model with a structure equal to (1, 2) with GADMA (Table 6, Fig. 10). For all 3 AFSs, such a model had the best log-likelihood among previously inferred models (Tables S2–S4). For the Northern and Southern populations, the demographic model with the (1, 2) structure also had the best AIC score. This model showed similar features for all observed data: an interval of time after the splitting of the ancestral population with unidirectional migration, followed by a time interval with bidirectional migration and population size change. The direction of the unidirectional migration was as follows: from CVLN to CrossRiver, from CVLN to CVLS, and from Southern to Northern. We also constructed 2 additional demographic models based on observed features and inferred their parameters: splitting of the ancestral population with unidirectional migration followed by either symmetric or asymmetric migrations with population size change. The demographic model with unidirectional migration followed by asymmetric migration and population size change was found to be the best for all 3 AFSs (Table 6): Northern and Southern populations: ΔAIC = 4.41, ω_*i*_ = 0.89; CVLN and CVLS populations: ΔAIC = 1.04, ω_*i*_ = 0.58; and CrossRiver and CVLN populations: ΔAIC = 0.04, ω_*i*_ = 0.44. But there were several additional demographic models that explained the data equally well: the same demographic model as just described but that also included symmetric migration for CrossRiver, CVLN (ω_*i*_ = 0.43), and secondary contact with asymmetric migration and population size change for CVLN and CVLS (ω_*i*_ = 0.34).

**Table 6:** Demographic models and associated parameters inferred in GADMA for 3 pairs of populations of the Gaboon forest frog: Northern and Southern, CVLN and CVLS, and CrossRiver and CVLN

	Northern, Southern			CVLN, CVLS			CrossRiver, CVLN		
	Best from dadi pipeline*^1^*	Model structure (1, 2)	Best from GADMA^2^	Best from dadi pipeline*^3^*	Model structure (1, 2)	Best from GADMA^2^	Best from dadi pipeline*^4^*	Model structure (1, 2)	Best from GADMA^2^
No. of parametirs	7	11	9	8	11	9	6	11	9
Log-likelihood	−439.91	−402.20	**−402.00**	−463.33	−453.67	**−453.65**	−377.99	**−365.26**	−365.29
AIC score	893.81	826.40	**821.99**	942.67	929.34	**925.30**	769.98	752.52	**748.58**
Parameters									
θ	573.9	134.7	107.0	572.18	134.17	145.5	287.96	250.58	251.88
η_1a	0.259	1.894	2.515	0.338	2.010	1.893	0.350	0.149	0.139
η_2a	0.247	2.634	3.339	1.331	0.840	0.880	6.746	7.034	6.899
m_12a	NA	0.046	0.033	NA	0.0	NA	NA	2.510	2.639
m_21a	NA	0.0	NA	NA	0.400	0.399	NA	0.001	NA
η_1b	3.121	13.544	17.139	4.528	13.220	12.266	$=\nu ^a_{1}$	0.974	0.889
η_2b	1.291	5.584	7.057	1.193	3.620	3.349	$=\nu ^a_{2}$	8.707	8.873
m_12b	0.049	0.008	0.006	0.108	0.0419	0.046	1.272	0.507	0.556
m_21b	$=m^b_{12}$	0.017	0.014	1.084	0.365	0.395	0.221	0.328	0.312
* T_a*	0.302	4.335	5.779	0.325	5.000	4.148	0.495	1.110	1.089
* T_b*	0.556	2.162	2.692	0.870	1.970	1.808	0.402	0.101	0.109

^1^ Divergence, secondary contact with population size change and symmetric migration.

^2^ Divergence, unidirectional migration, followed by population size growth and bidirectional asymmetric migrations.

^3^ Divergence, secondary contact with no population change and asymmetric migration.

^4^ Divergence, secondary contact with no population change and asymmetric migration.

θ = 4*N*_Aμ*L*, μ — mutation rate per generation per site, *L* — the effective sequence length; η_*px*—relative (to *N*_A) size of population *p* (1 for Northern/CVLN/CrossRiver, 2 for Southern/CVLS/CVLN) during time interval *x* after divergence (*a*—first time interval, *b*—second time interval); m_*pkx*—scaled (by 2*N_*A) migration rate from population *k* to population *p* during time interval *x; T_x*—scaled (by 1/(2*N*_A)) time of time interval *x*. NA: not applicable. Expected AFS was simulated using ∂a∂i with *G* = {50, 60, 70} for Northern, Southern populations and with *G* = {40, 50, 60} for other cases. See text for more details. Best log likeliood value and AIC score are marked in bold.

## Discussion

We report the development and mathematical justification of GADMA and demonstrate its effectiveness using several previously published datasets. Our method is based on the GA and uses existing solutions from either ∂a∂i or moments to simulate the AFS from the proposed demographic model. GADMA is the first program that allows the automatic inference of demographic history of up to 3 populations from an AFS. Existing optimizations, implemented in either ∂a∂i or moments, require prior specification of demographic models to be tested and are thus inefficient in practice, given the large number of possible demographic scenarios that can be constructed for 1 or more populations. Our method is implemented in the GADMA software, which is openly accessible (see Availability of Source Code and Requirements).

GADMA was shown to be efficient in performance: it was applied to both simulated data and to 3 empirical datasets, representing different organismal systems and associated demographic histories. The inferred demographic models had better log-likelihood scores than those reported in the original papers, which were derived from optimizations using either ∂a∂i or moments alone. Moreover, the demographic histories inferred with GADMA were consistent with the known history of the 3 taxa [11,31,37]. We also demonstrated the stability of the search, starting with demographic models with simpler structures rather than more complex ones, which reflects the profitability of using a search scheme that includes an increase in model structure complexity. Additionally, we compared pipelines using moments or ∂a∂i and showed that the computational speed of moments was much greater than for ∂a∂i. Thus, GADMA is the first software that effectively infers a demographic model from an AFS with nothing required from the user, except the structure of the demographic model, which determines the extent of the model complexity and associated details.

Despite the increasing use of AFSs for inferring demographic history, there are some limitations of the informativeness of AFS with regards to historical demographic inference that have been noted (see Beichman et al. [3]). For example, previous studies have shown that the AFS of a single panmictic population can be matched to different demographic scenarios [9]. We also observed this issue in our simulated data for the demographic model that included a bottleneck event of a panmictic population. The inferred model with an earlier exponential bottleneck had almost the same likelihood score as the model with the sudden population size dynamic. We should expect the same behavior in the case of multiple populations, which requires estimating the joint AFS. Moreover Rosen et al. [40] showed the limitation of AFS-based methods and their pathological behavior. They also proved that expected AFS data from *n* samples under any demographic model could be generated by a piecewise-constant model with ≥2*n* − 1 time intervals. This research is a complement to the paper Terhorst and Song [41], who showed the minimax error of the inference of population size history to be $\geq\mathcal {O}(1/\log {}(s))$, where *s* is the number of segregating sites. This result means that the accuracy of demographic inference does not depend on the size of AFS but on the number of considered segregating sites. Although it was provided only for the populations that have experienced a bottleneck, the authors argue that this behavior should be expected for the real data. We should consider described limitations of AFS, and because of such behavior, the structure of the demographic model should not be very complex. We suggest using structures no more than (2, 1) and (2, 1, 1). This limitation can be solved by using additional information about observed populations, e.g., 2-locus statistics [42] or focus on inverse instantaneous coalescence rate summary [43]. Incorporating genetic linkage information with AFS data could also improve the accuracy of inference of demographic history [42]. Including such information in the GADMA pipeline will allow the use of more complex demographic model structures in the future.

In our analyses using the AFS from 3 human populations (YRI, CEU, and CHB), we inferred a best-fitting demographic model that showed an expansion out of Africa ∼400,000 years ago, which is not supported by previous studiess [11, 44,45]. This can be caused by the fact that tree-based models often do not take into account processes such as admixture and introgression that can under- or overestimate population divergence times (e.g., Kamm et al. [13]). Alternatively, these differences could result from the limitation of the informativeness of the AFS or by noise in the spectrum as a result of including low-quality variant calls because AFS-based methods should be highly sensitive to noise [40]. However, Gutenkunst et al. [11] noted the high quality of this dataset. The demographic model inferred with GADMA using the same parameters as in Gutenkunst et al. [11], under the assumption that expansion took place not earlier than 150,000 years ago, resulted in parameter values similar to those reported in Gutenkunst et al. [11]. We also inferred all possible parameters, including asymmetric migration rates and different dynamics of population size changes, and obtained a demographic model with the best CLAIC score. With GADMA, we observed higher asymmetric migration rates and the growth of the CEU+CHB population after its split from the African population.

AFSs of 2 isolated populations of Gillette’s checkerspot butterfly from Wyoming and Colorado showed several alternative models with values very close to the best value of the composite likelihood found by McCoy et al. [31]. All migrations that were inferred are negligible, which confirmed the isolation of the 2 populations. One of the inferred models is consistent with the demographic history that was estimated previously by McCoy et al. [31]. The demographic model inferred using GADMA with the best likelihood value seems to be a better model overall because, in addition to the best CLAIC score (for the AFS using all SNPs), it correctly inferred the timing of the population split to the actual known value of 33 years. However, the model without migration showed the best CLAIC score for the AFS using all synonymous SNPs and could be the right choice too, so we suggest that further research is necessary to identify alternative models that may better fit the demographic history of these checkerspot butterfly populations.

We conducted a series of experiments for selecting demographic models for the Gaboon forest frog, also repeating the analyses performed by Portik et al. [32]. Nearly all of the 12 models inferred previously were found to be suboptimal. For 2 of the 3 population sets analyzed with GADMA, demographic models with higher log-likelihoods were chosen compared to those previously inferred. For the comparison that included the CVLN and CVLS populations, the demographic model with the highest log-likelihood was consistent with the model inferred by Portik et al. [31], but new values of parameters with better likelihood values were found. Demographic model optimization using ∂a∂i proved to be unstable, and we were consequently forced to either specify lower limits on specific parameter values or instead use moments for model optimization. moments proved to be indeed more stable in simulating the expected AFS from the demographic model. We then inferred the full parameters of the demographic model with a structure equal to (1, 2) for each of 3 AFS datasets. For Northern and Southern populations, models using this structure resulted in higher log-likelihood scores. However, we noticed some peculiar properties in parameter values and generated new demographic models based on these properties and inferred their parameters using the 3 AFSs. These analyses resulted in a model with improved AIC scores for each of the 3 AFSs. This model contains divergence of the ancestral population, a time interval with unidirectional migration followed by a time interval with population size change and bidirectional asymmetric gene flow.

In this work, we focused on benchmarking the effectiveness of the GA for demographic model inference on real data. We found that GADMA managed to infer better demographic models than those that were found in the original analyses for all tested datasets. However, we highlight 2 caveats that warrant further research. First, our method only used AFS data. Other data, such as those based on haplotypes or SNP data may prove to be more informative about demographic history, but current methods using such data are restricted to simple population size change dynamics (e.g., identical by state [IBS] tract method [34], DIYABC [46], MSMC [47]) whereas ∂a∂i and moments, which are implemented in GADMA, are less restricted in these dynamics. Second, it is possible to add other solutions like fastsimcoal2 or momi2 for simulating the expected AFS from demographic models and make comparisons between all methods. Also, even though we performed some analyses on simulated data that included models with known global optima, we suggest our method could be further verified through the use of additional simulated datasets.

While the optimization search implemented in GADMA is able to find demographic models with the best likelihood score and their associated parameters, it is important to minimize the number of parameters so as to avoid the possibility of overfitting the model to the AFS data. Fortunately, such a strategy is included in GADMA using AIC and CLAIC scores: it informs the user about overfitting when the demographic model with the best likelihood score and best AIC (or CLAIC) score do not match. Additionally, GADMA can infer demographic models with all possible parameters, allowing researchers to explore additional models based on the inferred model, as we demonstrated in the case of the Gaboon forest frog. However, we note that GADMA does not sort out all possible numbers of parameters, so it is not guaranteed to find the model(s) with best AIC (or CLAIC) score(s). Moreover, we recommend performing block bootstrapping of datasets over unlinked regions of the genome for accurate estimation of the CLAIC score. We note, however, that detection of such regions can be difficult.

Another direction in the further development of our work is increasing the number of considered populations. Currently, GADMA can analyze up to 3 populations, similar to ∂a∂i. In contrast, moments can simulate AFS for up to 5 populations. Because the limitations of AFS-based methods that were presented above are the important restrictions in increasing the number of populations and complexity of demographic models (we again propose the simplest structures then), we expect that this modification should be done in parallel with the incorporation of additional summaries of genetic data. Moreover, including the estimation of selection coefficients (e.g., Gutenkunst et al. [11]) and the development of a user-friendly interface for various types of datasets (e.g., all SNPs, synonymous SNPs only), will help to further expand the capabilities of GADMA. In this work we focused on the application of the GA as global optimization on the demographic inference. We have shown that effective global search is possible, and we assume the existence of a more powerful optimization method. Thus, it is also possible to improve the proposed method, using another optimizations or various modifications of the GA, e.g., one that infers deliberately different demographic models [48].

## Availability of Source Code and Requirements

- Project name: GADMA
- Version: 1.0.0
- Project home page: https://github.com/ctlab/GADMA
- RRID:SCR_017680
- biotoolsID: biotools:GADMA
- Operating system(s): Platform independent
- Programming language: Python
- Other requirements: Python (2.5, 2.6, 2.7), NumPy (≥1.2.0), Scipy (≥0.6.0), ∂a∂i (≥1.7.0) or moments (≥1.0.0)
- License: GNU GPL v3.

## Availability of Supporting Data and Materials

All data, parameters for GADMA runs, and results are available in the Bitbucket repository [49]. The supplementary materials and the archival snapshot of code are published as a GigaDB dataset [50]. Supplementary tables are presented in both PDF and tabular formats.

## Additional Files

Supplementary Figure S1. Diagrams of three primary demographic dynamics of population size change.

Supplementary Figure S2. Diagram showing the general algorithm used in GADMA.

Supplementary Table S1. Demographic models for the allele frequency spectrum using synonymous SNP's only of the Gillette's checkerspot butterfly.

Supplementary Table S2. Demographic models for Northern and Southern populations of Gaboon forest frog.

Supplementary Table S3. Demographic models for CVLN and CVLS populations of Gaboon forest frog.

Supplementary Table S4. Demographic models for CrossRiver and CVLN populations of Gaboon forest frog.

Supplementary Table S5. CLAIC values, calculated with different ϵ values of step size.

Supplementary Table S6. Results of 50 runs of optimizations on the simulated data for one population.

Supplementary Table S7. Results of 50 runs of optimizations on the simulated data for two populations.

Supplementary Table S8. Results of 10 runs of optimizations on the simulated data for three populations.

giaa005_GIGA-D-19-00143_Original_Submission

giaa005_GIGA-D-19-00143_Revision_1

giaa005_GIGA-D-19-00143_Revision_2

giaa005_GIGA-D-19-00143_Revision_3

giaa005_Response_to_Reviewer_Comments_Original_Submission

giaa005_Response_to_Reviewer_Comments_Revision_1

giaa005_Response_to_Reviewer_Comments_Revision_2

giaa005_Reviewer_1_Report_Original_Submission Ryan Gutenkunst -- 5/15/2019 Reviewed

giaa005_Reviewer_1_Report_Revision_1 Ryan Gutenkunst -- 9/27/2019 Reviewed

giaa005_Reviewer_1_Report_Revision_2 Ryan Gutenkunst -- 12/10/2019 Reviewed

giaa005_Reviewer_2_Report_Original_Submission Lounes Chikhi -- 6/10/2019 Reviewed

giaa005_Supplemental_File

## Abbreviations

AFS: allele frequency spectrum; AIC: Akaike information criterion; BFGS: Broyden-Fletcher-Goldfarb-Shanno; CLAIC: composite likelihood Akaike information criterion; CVLN: Cameroon Volcanic Line North; CVLS: Cameroon Volcanic Line South; GA: genetic algorithm; GADMA: Genetic Algorithm for Demographic Model Analysis; Mb: megabase pairs; PDE: partial diffusion equation; SNP: single-nucleotide polymorphism; SNV: single-nucleotide variant.

## Competing Interests

The authors declare that they have no competing interests.

## Funding

This work was financially supported by JetBrains Research and by the Government of the Russian Federation (Grant 08-08).

## Authors' Contributions

P.D. conceived and designed the project; E.N. developed the software presented in this article and wrote the draft manuscript.; V.U. provided guidance in algorithms development and evaluation; K.P.K was major contributor in review and editing of the manuscript.; V.U., S.J.O., and P.D. supervised the project. All authors contributed in writing the manuscript.
