# Supplementary material for: GADMA: Genetic algorithm for inferring demographic history of multiple populations from allele frequency spectrum data
Source: Gigascience. 2020 Feb 29;9(3):giaa005. doi: 10.1093/gigascience/giaa005 (PMC7049072; doi:10.1093/gigascience/giaa005)
Supplement: giaa005_GIGA-D-19-00143_Revision_3 [file giaa005_giga-d-19-00143_revision_3.pdf]

GigaScience

GADMA: Genetic algorithm for inferring demographic history of multiple populations from allele frequency spectrum data

--Manuscript Draft--

**Manuscript Number:**	GIGA-D-19-00143R3
**Full Title:**	GADMA: Genetic algorithm for inferring demographic history of multiple populations from allele frequency spectrum data
**Article Type:**	Technical Note
**Funding Information:**	
**Abstract:**	The demographic history of any population is imprinted in the genomes of the individuals that make up the population. One of the most popular and convenient representations of genetic information is the allele frequency spectrum or AFS, the distribution of allele frequencies in populations. The joint allele frequency spectrum is commonly used to reconstruct the demographic history of multiple populations and several methods based on diffusion approximation (e.g., ∂a∂i) and ordinary differential equations (e.g., moments) have been developed and applied for demographic inference. These methods provide an opportunity to simulate AFS under a variety of researcher-specified demographic models and to estimate the best model and associated parameters using likelihood-based local optimizations. However, there are no known algorithms to perform global searches of demographic models with a given AFS. Here, we introduce a new method that implements a global search using a genetic algorithm for the automatic and unsupervised inference of demographic history from joint allele frequency spectrum data. Our method is implemented in the software GADMA (Genetic Algorithm for Demographic Model Analysis, https://github.com/ctlab/GADMA). We demonstrate the performance of GADMA by applying it to sequence data from humans and non-model organisms and show that it is able to automatically infer a demographic model close to or even better than the one that was previously obtained manually. Moreover, GADMA is able to infer multiple demographic models at different local optima close to the global one, providing a larger set of possible scenarios to further explore demographic history.
**Corresponding Author:**	Ekaterina Noskova<br><br>RUSSIAN FEDERATION
**Corresponding Author Secondary Information:**	
**Corresponding Author's Institution:**	
**Corresponding Author's Secondary Institution:**	
**First Author:**	Ekaterina Noskova
**First Author Secondary Information:**	
**Order of Authors:**	Ekaterina Noskova
	Vladimir Ulyantsev
	Klaus-Peter Koepfli
	Stephen J. O’Brien
	Pavel Dobrynin
**Order of Authors Secondary Information:**	
**Response to Reviewers:**	Dear Editor,<br><br>We are grateful for your comments and believe that our edits will improve the clarity of the manuscript. A point-by-point response can be found below:

Powered by Editorial Manager® and ProduXion Manager® from Aries Systems Corporation

	"1) Supporting data / archival snapshot of code in GigaDB:<br><br>Our data curators will contact you shortly to prepare any supporting data (and/or an archival copy of the code) for release via our repository GigaDB. Once this is sorted, please include a citation to your upcoming GigaDB dataset (including the DOI link) to your reference list, and cite this dataset in the "data availability" section and elsewhere in the manuscript, where appropriate.<br><br>Please follow this example for the reference:<br><br>[xx] Author1 N, Author2 N, AuthorX N. Supporting data for "Title of your manuscript". GigaScience Database. 2019. http://dx.doi.orgxxxxxxxxxxxx<br><br>You will get the exact citation, including the DOI link, from our curators."<br><br>We have published the supplementary materials as GigaDB dataset and cited it in the manuscript.<br><br>"2) Citing your bitbucket repository.<br>I see you link to a bitbucket repository in the supplemental files. Please also cite this in in the "availability" section of the paper and include a reference to the bibliography, including a DOI if available. (i.e. please cite the bitbucket repo the same way as the GigaDB data, see above).<br><br>If data and code has been modified in the revision process please be sure to update the public versions (e.g. in github or bitbucket) of this too."<br><br>Bitbucket repository was added to the references and cited in the "Availability of supporting data and materials" section of the manuscript.<br><br>"3) We seem to have the tables and parameters in the supplemental files in PDF format only. I feel it would be better for re-use and reproducibility if these data and parameters are made available in re-usable, tabulated form (you may be able host them in GigaDB or bitbucket - our data curators can give advice if needed)."<br><br>Supplementary tables are presented in both formats (PDF and tabular format) in GigaDB dataset.<br><br>"4) Please register your new software application in the bio.tools and SciCrunch.org databases to receive RRID (Research Resource Identification Initiative ID) and biotoolsID identifiers, and include these in the "availability and requirements" section of your manuscript. This will facilitate tracking, reproducibility and re-use of your tool."<br><br>We have registered GADMA in this databases and included received ID's in the "Availability of source code and requirements" section of the manuscript.<br><br>"5) At this stage, please remove any yellow highlighting that was made for the purpose of peer review."<br><br>We removed highlighting.
**Additional Information:**	
**Question**	**Response**
Are you submitting this manuscript to a special series or article collection?	No
**Experimental design and statistics**<br><br>Full details of the experimental design and statistical methods used should be given in the Methods section, as detailed in our	Yes

Powered by Editorial Manager® and ProduXion Manager® from Aries Systems Corporation

Minimum Standards Reporting Checklist. Information essential to interpreting the data presented should be made available in the figure legends.<br><br>Have you included all the information requested in your manuscript?	
**Resources**<br><br>A description of all resources used, including antibodies, cell lines, animals and software tools, with enough information to allow them to be uniquely identified, should be included in the Methods section. Authors are strongly encouraged to cite Research Resource Identifiers (RRIDs) for antibodies, model organisms and tools, where possible.<br><br>Have you included the information requested as detailed in our Minimum Standards Reporting Checklist?	Yes
**Availability of data and materials**<br><br>All datasets and code on which the conclusions of the paper rely must be either included in your submission or deposited in publicly available repositories (where available and ethically appropriate), referencing such data using a unique identifier in the references and in the “Availability of Data and Materials” section of your manuscript.<br><br>Have you have met the above requirement as detailed in our Minimum Standards Reporting Checklist?	Yes

Manuscript
Click here to access/download;Manuscript;manuscript_R3.pdf

OXFORD (GIGA)^n SCIENCE

GigaScience, 2018, 1–16

doi: xx.xxxx/xxxx
Manuscript in Preparation
Technical note

TECHNICAL NOTE

GADMA: Genetic algorithm for inferring demographic history of multiple populations from allele frequency spectrum data

Ekaterina Noskova[1] *, Vladimir Ulyantsev[1], Klaus-Peter Koepfli[1, 2], Stephen J. O'Brien[1, 3] and Pavel Dobrynin[1, 2]

[1]Computer Technologies Laboratory, ITMO University, St. Petersburg, Russian Federation and [2]Smithsonian Conservation Biology Institute, Center for Species Survival, National Zoological Park, Washington, D.C., USA and [3]Guy Harvey Oceanographic Center, Nova Southeastern University Ft. Lauderdale, Florida, USA

* noskova.e.ekaterina@gmail.com

Abstract

The demographic history of any population is imprinted in the genomes of the individuals that make up the population. One of the most popular and convenient representations of genetic information is the allele frequency spectrum or AFS, the distribution of allele frequencies in populations. The joint allele frequency spectrum is commonly used to reconstruct the demographic history of multiple populations and several methods based on diffusion approximation (e.g., ∂a∂i) and ordinary differential equations (e.g., *moments*) have been developed and applied for demographic inference. These methods provide an opportunity to simulate AFS under a variety of researcher-specified demographic models and to estimate the best model and associated parameters using likelihood-based local optimizations. However, there are no known algorithms to perform global searches of demographic models with a given AFS. Here, we introduce a new method that implements a global search using a genetic algorithm for the automatic and unsupervised inference of demographic history from joint allele frequency spectrum data. Our method is implemented in the software GADMA (Genetic Algorithm for Demographic Model Analysis, https://github.com/ctlab/GADMA). We demonstrate the performance of GADMA by applying it to sequence data from humans and non-model organisms and show that it is able to automatically infer a demographic model close to or even better than the one that was previously obtained manually. Moreover, GADMA is able to infer multiple demographic models at different local optima close to the global one, providing a larger set of possible scenarios to further explore demographic history.

Key words: demographic inference; population genetics; genetic algorithm; allele frequency spectrum, genomics

Introduction

To understand the evolution of species and their populations, it is important to understand what events occurred in their past and when. The genetic diversity and structure of species are shaped by the combined processes of changes in effective population size, population divergence and/or migration (gene flow) operating over the course of thousands of generations. Records of population history are imprinted in the genomes of individuals within species and this history can be inferred using a variety of algorithmic and statistical methods. With the rise of next-generation sequencing (NGS) technologies and abundant genome data, it has become possible to explore complex and parameter-rich demographic models that include the estimation of mutation rate, changes in effective population size, nonrandom mating, admixture, and selection [1, 2]. However, given the infinitely large number of permutations at which

Compiled on: January 9, 2020.
Draft manuscript prepared by the author.

Figure 1. General scheme of ∂a∂i. In order to compare a demographic model and the real observed AFS, ∂a∂i extracts the expected AFS from the demographic model using a numerical solution of the partial differential equation (PDE) that corresponds to that demographic model, and calculates the composite likelihood between the expected and observed allele frequency spectrum.

these processes operate over various time intervals, there is no method that can guarantee to find the demographic model that best fits the observed data.

One of the primary methods for inferring demographic models from genomic data is based on the allele frequency spectrum (AFS), also known as the site frequency spectrum [2, 3]. In essence, the AFS describes the distribution of derived allele frequencies of bi-allelic loci (SNVs) in a population or sample of populations based on the number of sequenced chromosomes [4]. An AFS can provide information about how the populations developed based on observed genetic variation sampled from current individuals of those populations. Many studies have been devoted to testing and understanding the behavior of allele-frequency spectra under different demographic scenarios [5, 6, 7, 8, 9].

Two of the most popular methods of historical demographic inference based on AFS are the faster continuous-time sequential Markovian coalescent approximation (fastsimcoal2, [10]) and the diffusion approximation (∂a∂i, [11]). fastsimcoal2 can successfully handle more than three populations, but it is computationally challenging because it simulates multiple AFS simultaneously to estimate the most stable one. ∂a∂i simulates AFS using a numerical solution of the partial diffusion equation (PDE), which corresponds to the presented demographic model and then provides the likelihood of the model (Figure 1). Unfortunately, PDE leads to some computational difficulties associated with analyses of complicated demographic models and large sample sizes. As a result, ∂a∂i can only handle up to three populations. More recently, two new methods called *moments* [12] and Moran Models of Inference (momi2; [13]) have been introduced. *moments*, like ∂a∂i, is based on two models of population genetics: the Wright-Fisher generation model and the infinite sites mutational model, whereas momi2 is based on another generation model — the Moran model [14]. As momi2 is a new method, we have not considered it in the present study. *moments* uses ordinary differential equations to simulate AFS, which is faster and more stable than diffusion approximation in ∂a∂i, based on simulations comparing the two methods [12]. *moments* presents a tradeoff between speed and accuracy in AFS-based demographic inference, can handle up to five populations and provides the same interface as ∂a∂i.

Ideally, researchers seek to find the model of demographic history that best describes or "fits" their data. ∂a∂i and *moments* provide an opportunity to run multiple optimizations to help fit parameters of a given demographic model that maximizes the value of the composite likelihood. But optimizations based on gradient descent, for example, the Broyden-Fletcher-Goldfarb-Shanno (BFGS) algorithm [15, 16, 17, 18] or its modifications, use numerical differentiation and are ineffective in practice, because of the complex structure of the search space for demographic models. Optimizations may also be inefficient due to another set of methods that offer existing solutions based on local search algorithms without gradients, such as the Nedler and Mead method [19] or Powell's method [20]. As a result, all existing optimizations find local optima close to the initial values and require many runs to be performed using different initial model parameters, most of which are unknown or lack empirical data. Despite these drawbacks, ∂a∂i and *moments* are efficient instruments, since they can simulate an allele frequency spectrum from any demographic model. In other words, the problem of finding a demographic model from the allele frequency spectrum is the

Figure 2. Examples of different allele frequency spectra. (A) Isolation of a population after divergence; (B) Low symmetrical migrations between populations following divergence; (C) High asymmetrical rates of migrations between populations following divergence.

inverse problem, which can be solved by solving the direct problem, that is simulation of the AFS from a given demographic model, with approximate numerical methods, such as diffusion or *moments* approximations. The lack of accurate and rapid differentiation and the complex structure of the search space led us to consider the usage of global optimization methods, such as the genetic algorithm.

The genetic algorithm (GA) [21] is one of the most efficient heuristic algorithms for global searching of complex and rich parameter space. Its primary application is optimization of a fitness function, which is either not differentiable or it cannot be differentiated in a sufficiently effective way, for example, when the function is not representable in "closed-form expression". GA is based on the principle of evolution and simulates natural selection using operations of "mutation" and "crossover", which ideally results in the most adapted individual, the one which has the best value of the fitness function. The versatility of the GA has led to its wide application, including reconstruction of phylogenetic trees [22], ancestral genome composition inference [23], and evolutionary biology in general [24].

In this paper we present a new method based on the genetic algorithm to automatically infer the best fitting demographic model from AFS data for two or three populations. Our method assumes the ability to simulate the AFS from the demographic model, for example, using either ∂a∂i or *moments*. The GA framework overcomes limitations of local search optimizations and is more flexible in handling the complexity of demographic models by allowing an increase in the number of parameters and estimation of parameters such as functions of population size changes that are sudden, linear or exponential. We implemented our method in the software GADMA (Genetic Algorithm for Demographic Model Analysis), which is written in Python and available at Github (`https://github.com/ctlab/GADMA`).

Materials and Methods

This section provides definitions of the allele frequency spectrum and the composite likelihood scheme that is used in existing optimizations (∂a∂i and *moments*) and implemented in our method. After this background, the problem of demographic model search from observed AFS data is formulated in terms of computer science. We then introduce a developed representation of a demographic model for a general approach to our method, including the genetic algorithm with its operations of "mutation" and "crossover".

Basic definitions and concepts

Assume there are P populations and for each population i there exists information about n_i chromosomes. The AFS is the P-dimensional array A, where each entry $A[d_1, \ldots, d_P] \in \mathbb{N}$, $d_i \in [0, n_i]$, $\forall i \in [1, P]$ records the number of SNVs (relative to the common reference genome), which are exactly seen at d_1 chromosomes from population 1, d_2 chromosomes from population

2, . . . and d_P chromosomes from population P. For example, if we have two populations, then the AFS is a two-dimensional matrix, where $A[i, j]$ represents the number of polymorphisms that occurred exactly in i individuals in the first population and in j individuals in the second population (Figure 1). Several examples of allele frequency spectra are presented in Figure 2.

Assume that we can simulate the AFS M from the proposed demographic model. Assuming no linkage between derived alleles, each element of the allele frequency spectrum $S[d_1, \ldots, d_P]$ is an independent Poisson value with a mean equal to $M[d_1, \ldots, d_P]$. We then calculate the likelihood — the probability of obtaining the observed spectrum S, if the expectation spectrum is M, as the product of $(n_1 + 1)(n_2 + 1) \ldots (n_P + 1)$ Poisson likelihood functions:

$$\mathcal{L}(M|S) = \prod_{\substack{d_i=0,..,n_i,\\ i=1..P}} \frac{e^{-M[d_1,\ldots,d_P]} M[d_1, \ldots, d_P]^{S[d_1,\ldots,d_P]}}{S[d_1, \ldots, d_P]!}$$

In the case of linked alleles, $\mathcal{L}(M|S)$ is the composite likelihood. Demographic models inferred by ∂a∂i and *moments* can be compared by computing the $\log(\mathcal{L}(M|S))$. Because $\mathcal{L}(M|S) \in [0, 1]$, then $\log(\mathcal{L}(M|S)) \in [-\inf, 0]$ and the greater is the log-likelihood, the better the model fits the observed AFS. In this paper, $\log(\mathcal{L}(M|S))$ was chosen as the fitness function of the genetic algorithm, as discussed below.

Formulation of the problem

Consider a function $f(\Theta, A, C)$ that takes the parameters $\Theta = \{\theta^k\}_{k=1}^{N_\Theta}$, $\theta^k \in \mathbb{R}$, the AFS $A \in \mathbb{R}^{P \times P}$, the set of constants $C = \{c^k\}_{k=1}^{N_C}$ and returns the measure of the correspondence between the parameters Θ and the AFS, A.

The function $f(\Theta, A, C)$ builds a demographic model with respect to the parameters Θ that unambiguously determine it, calculates the expected AFS M with respect to the constants C, and then determines the degree of similarity between M and the observed A by the composite likelihood. The constants can be various parameters of algorithms for calculating the expected AFS, such as grid sizes for the numerical solution of a differential equation in ∂a∂i, or population models parameters, including the average number of new mutated sites per individual in a generation θ_0 for the infinite-sites mutational model, or the time T_g for one generation in the Wright-Fisher model. The function f can have different implementation details. Here ∂a∂i and *moments* were selected for this purpose.

The purpose of this work is to develop an algorithm to search for the demographic model that best corresponds to the observed AFS. Formally, the problem can be formulated as follows:

Input

- $A \in \mathbb{R}^{P \times P}$ — the P-dimensional array, $P \in \{2, 3\}$.
- $C = \{c_k\}_{k=1}^{N_C}$ — the set of constants.

Output

- The set $\Theta \in \mathbb{R}^{N_\Theta}$ of values, that maximize the value of f:

$$\Theta : \quad f(\Theta, A, C) \to \max$$

There are approximate solutions of this problem with an additional input — a demographic model with a fixed number of parameters, using various local search algorithms, but in practice, as mentioned above, these algorithms have proven to be ineffective. We present a new algorithm for the approximate solution of this more general problem using one of the most effective methods of global optimization — the genetic algorithm.

Representation of the demographic model and its structure

Assume a division of one ancestral population into two new isolated subpopulations. Then the number of population splitting events directly depends on P, the number of considered populations. We represent the demographic model as a sequence of "time intervals" and population splits, each of which has a fixed number of parameters. Assume a fixed temporal order of the current observed populations: from an ancient ancestral population to the more recently formed subpopulations. This temporal order is usually known or can be imputed. If the number of populations is ≤ 3, then each split will divide the last formed population. Thus, a splitting event has only one parameter — the fraction of the population, which separates to form a new subpopulation.

The next important component of the demographic model is the concept of the time interval. First, we define this as a segment of time during which a certain dynamic of change of effective size is maintained for each population. We consider three main demographic dynamics of population growth: sudden, linear and exponential change of the effective population size (Figure S1). Sudden change is very popular for its simplicity, but exponential change is a commonly used model for population growth as well. We include linear change as it is tradeoff between sudden and exponential change and is more realistic than sudden change. Secondly, the parameters of migration rates between populations are constant during a given time interval. Thus, each time interval has the following parameters:

- time,
- effective population sizes at the end of the time interval,
- demographic dynamics of effective population size change,
- migration between populations, if there is more than one population.

The sizes of the populations at the beginning of any time interval are equal to the sizes of the populations at the end of the previous interval. The first time interval is a special one: we consider that it lasts from the beginning of the existence of the species and assume a demographic dynamic of sudden change for the effective population size of the ancestral population [11]. Therefore, in this interval the only parameter estimated is the size of the ancestral population. Note that the number of splitting events is determined by the number of populations under consideration, but the number of intervals can be varied and thus change the number of parameters of the demographic model, its detail, and complexity.

We now can define the concept of the **structure of the demographic model**. In the case of an ancestral population splitting into two subpopulations, the structure of the model will include a number of time intervals that occur before and after a single splitting event. In the case of three populations, the structure includes a number of time intervals prior to the first split, those between the first and second split, and the ones after the second split. For example, assume we observe three populations. At the beginning, there was an ancestral population (P_A) and this population started to grow in effective size. Then a split occurred that divided this ancestral population into two daugh-

Figure 3. Example of a demographic model with a (2,1,1) structure. Time is shown along the x-axis and population size on the y-axis. Four time intervals are shown: T_1, T_2, T_3 and T_4, and two populations splits: S_1 and S_2. The structure of this model is (2,1,1) because T_1 and T_2 are time intervals for one ancestral population, followed by split S_1, T_3 is the time interval for two populations, and T_4 is the time interval for three final populations after the second split S_2. Time interval T_2 has the following parameters: time of this interval, size of the ancestral population at the end and the dynamic of size change. Time intervals T_3 and T_4 for each population will contain the same parameters plus migrations between populations. Split events S_1 and S_2 have fraction of size split as parameter. The first interval contains the size of an ancestral population, but it can be ignored as it could be implicitly evaluated from other parameters [11].

ter populations (P_1 and P_2) that changed in effective size during one interval, followed by a split of the second population (into P_{2a} and P_{2b}), resulting in three descendant populations that changed within one subsequent time interval. The structure of such a model would be described as (2,1,1) (Figure 3). The simplest model structures would be for two populations — (1,1), and for three populations — (1,1,1).

More formally, the structure of the model is a sequence of the form $S^* = \{s_i^*\}_{i=1}^P$, $s_i^* \in \mathbb{N}$, where $P \in \{2, 3\}$ is the number of populations. In this case, the number of parameters $N_\Theta(S^*)$ of the demographic model with the structure S^* will be determined as follows:

$$N_\Theta(S^*) = (P-1) + \sum_{i=0}^{P} N_\Theta^i(s_i^*),$$

$$\text{where} \quad N_\Theta^i(s_i^*) = \begin{cases} 3(s_1^*-1), & \text{if } i=1, \\ 7s_2^*, & \text{if } i=2, \\ 13s_3^*, & \text{if } i=3. \end{cases}$$

The term $(P-1)$ corresponds to the number of split parameters, and $\sum_{i=0}^{P} N_\theta^i(s_i^*)$ is the number of time intervals parameters. This number of parameters is valid for the genetic algorithm that is described below, but the number of final model parameters is different. During the local search, which occurs after the genetic algorithm, the dynamics of population size change are fixed and the final number of parameters $\overline{N}_\Theta(S^*)$ is:

$$\overline{N}_\Theta(S^*) = (P-1) + \sum_{i=0}^{P} \overline{N}_\theta^i(s_i^*),$$

$$\text{where} \quad \overline{N}_\Theta^i(s_i^*) = \begin{cases} 2(s_1^*-1), & \text{if } i=1, \\ 5s_2^*, & \text{if } i=2, \\ 10s_3^*, & \text{if } i=3. \end{cases}$$

Thus, we can unambiguously interpret the demographic model according to the list of parameters and its structure by fixing for each time intervals a certain order of parameters.

General approach

The general algorithm is a series of executions of the genetic algorithm (Figure S2). Suppose we have the initial and final structures that define the initial and final number of parameters (definition of the model structure is presented in the previous section) of the demographic models, derived from considerations about the populations we are trying to model. During the genetic algorithm, the structure of the model does not change; that is, the model's parameters for the current structure are optimized. This restriction makes the procedure of crossover in the genetic algorithm simpler. As soon as the genetic algorithm stops, the parameters of the best-fitting model are adjusted in order to improve the likelihood with a local search algorithm. If the structure of the model is not quite complex enough, that is, some value in the model structure (e.g., model structure (1,1,1)) is less than the corresponding value in final model structure (e.g., model structure (2,1,1)), its complexity (in terms of parameters) is increased and the genetic algorithm is run again for the new model structure. The genetic algorithm and local search are executed until the best-fitting parameters of the model with the final model structure are obtained.

Akaike information criterion.

With an increase in the number of model parameters, we risk overfitting the model. A model with a large number of parameters will be better able to find parameter values corresponding to the observed data than a model with a smaller number of parameters, but at the same time it will correspond less to reality, for example, due to data errors. The Akaike information criterion (*AIC*) [25] is commonly used to compare models with different numbers of parameters:

$$AIC(M(\theta), S) = 2 \cdot k - 2 \cdot \log(\mathcal{L}(M(\theta)|S)),$$

where k is number of parameters of the model and $\log(\mathcal{L}(M(\theta)|S))$ is the value of the log-likelihood function.

The composite likelihood Akaike information criterion (*CLAIC*) is a modification of the *AIC* for composite likelihoods, which is important to implement when SNPs that are used to build the AFS are linked [26, 27]. *CLAIC* is defined as follows:

$$CLAIC(M(\theta), S) = 2 \cdot tr(J(\theta)H^{-1}(\theta)) - 2 \cdot \log(\mathcal{L}(M(\theta)|S)),$$

where $J(\theta)$ and $H(\theta)$ are the variability and Hessian matrices, respectively:

$$J(\theta) = E_\theta \left\{ \frac{\partial \mathcal{L}(\theta|S)}{\partial \theta} \left(\frac{\partial \mathcal{L}(\theta|S)}{\partial \theta} \right)^T \right\}$$

$$H(\theta) = E_\theta \left\{ -\frac{\partial^2}{\partial \theta \partial \theta^T} \mathcal{L}(\theta|S) \right\}$$

The smaller the *AIC* or *CLAIC* score is, the better the model fits the observed data. In practice, calculation of the *CLAIC* is very challenging. Coffman et al., 2015 [26] applied bootstrapping to adjust composite likelihoods during statistical inference of demographic history using the programs ∂a∂i and TRACTS [28] and thereby calculate the *CLAIC*. This implementation was included in GADMA. Therefore, to obtain an accurate *CLAIC* score, one should perform block bootstrapping over unlinked regions of the genome.

Obviously, for *AIC* it is enough to compare only the final models for each model structure after the local search, since the number of parameters between the increases in model structure does not change, and therefore the value of the *AIC* score depends only on the likelihood values. In the implementation of GADMA, if the models with the best likelihood and best *AIC* or *CLAIC* (depending on whether SNPs are linked or not) score do not match, the user is informed about the overfitting.

Figure 4. Diagram showing the scheme of the genetic algorithm used in GADMA.

Figure 5. Diagram showing how mutation is used to change parameters of the demographic model in GADMA. In this example, ancestral population size is mutated at the second time interval prior to a population splitting event.

Figure 6. Diagram showing how crossover of two demographic models is performed in GADMA.

There are other methods for determining the selection of the best demographic model given an AFS data set . For example, likelihood ratio tests, which were introduced by Coffman et al., (2015) [26]. However, it is not possible to use them, because they assume nested demographic models, which is incorrect in our case, as the dynamics of population change can vary during the genetic algorithm.

Genetic algorithm

The genetic algorithm is one of the most effective heuristic algorithms [21]. It is based on the principles of evolution, where the aim of the algorithm is to find an approximate solution to a problem that has the maximum or minimum value of the fitness function. At the beginning of the algorithm, a fixed-sized set of random solutions, called individuals, is created. The set itself is called a generation. Each individual is assigned a value of fitness, which is determined by the value of the fitness function. After this, new generations are iteratively produced with the help of mutations, crossover and selection of the fittest individuals (i.e,. the models with the highest likelihood scores). All these operations can be either deterministic or random, and their order can vary in different implementations. In our case, individuals are demographic models of the same structure, and the fitness function is the log-likelihood, $\log(\mathcal{L}(M|S))$, as described in Materials and Methods.

In the first step of GADMA, a set of demographic models are randomly generated, if they have not been already specified. To form a new generation of demographic models, we select the most adapted models among a set of mutated, crossed, and random models in the previous generation. The value of the fitness function is used to select the most adapted models. The choice of models to be mutated or have crossover is random, but the probability of choice is directly proportional to the value of fitness: the better the fitness of the model is, the more likely it is to be selected. The genetic algorithm stops when it can no longer obtain a better demographic model by the value of the fitness function for several iterations (Figure 4).

Mutation of the demographic model.

The mutation of the demographic model (Figure 5) is equal to the process of changing the values of several parameters. There are two constants associated with mutation of the model: the rate and the strength of mutation. The number of parameters to be mutated is sampled from a binomial distribution with a mean equal to the mutation strength. Parameters that are mutated are chosen with the probability that is directly proportional to their weights, which at the beginning are equal (that is, the choice is equally probable), and then each weight can be increased when a mutation of the corresponding parameter has occurred, which leads to an improvement in the model. The measure of how much the value of each parameter is mutated is determined by the sign (+1 or −1, which are equally probable) and the rate of the mutation that is randomly sampled from the normal distribution, with the mean equal to the mutation rate and a variance equal to half of the mean. Among the parameters that can be mutated during estimation of the demographic model is the mode of population size change (sudden, growth, and exponential). If this parameter is chosen to be mutated, then the value (mode) will change to one of the other two population size change dynamics with equal probability.

Adaptive mutation rate and mutation strength.
In the initial iterations of the genetic algorithm, strong mutations of a large number of parameters are much more effective than weaker mutations of a small number of parameters, whereas when approaching the optimal solution the opposite is true. Therefore, the rate and strength of mutation can be adaptive, that is, it changes during the operation of the algorithm. There are several ways to make an adaptive value, one of the most popular being the one-fifth algorithm [29]. First, we apply it to the mutation rate: at each iteration, if we have a "successful" solution, that is, it becomes better after mutation, then we multiply the mutation rate by the constant $C \in [1, 2]$. If the solution is not "successful", then we divide by a fourth-degree root of C, decreasing the mutation rate. In the case of the mutation strength for the "successful" solution, it is necessary to additionally check whether the decision has become the best solution during the entire course of the algorithm's run.

Thus, often getting a new best solution with a mutation that occurs at the beginning of the genetic algorithm, we increase the number of parameters that are changed during the mutation operation and the degree to which they are changed. As we approach the optimum solution and decrease in frequency, the number of parameters will also decrease and lead to a more accurate search. An increase in the number of parameters being mutated leads to a more efficient crossover. At the same time, the mutation rate is changed more frequently than the mutation strength, which makes the mutation procedure more effective.

Crossover of two demographic models.
In order to have crossover of two demographic models, the models are represented as sequences of parameters. Each parameter of their descendant is chosen randomly with equal probability from one or the second parent (Figure 6). Since the structure of models do not change during the operation of the genetic algorithm, the number of parameters for all models will be the same. Consequently, the parameters can be unambiguously interpreted and easily crossed according to these criteria.

Local search algorithms.

Local optimizations are effective in cases when the initial solution is close to optimal. They are more accurate in adjusting parameters than the genetic algorithm, and can significantly improve its result. ∂a∂i and *moments* provide the following choice of algorithms for local optimization:

- The Broyden–Fletcher–Goldfarb–Shanno (BFGS) algorithm.
- L-BFGS-B — a modification of the BFGS algorithm, which is more efficient when the optimal parameters are close to the bounds.
- The Nelder–Mead method or simplex-method.
- Powell's method.

The first two methods are gradient descent optimization algorithms [30] and the last two do not use a gradient. Powell's

method was proposed by the authors of *moments* and was noted as the most effective, so it was adapted for usage with ∂a∂i and for our experimental studies (see below) it was chosen as the local search algorithm.

Increasing the complexity of the structure of the demographic models

We need to be able to increase the complexity of the structure of the demographic model in order to find an optimal solution. To do this, a time interval is selected and then divided into two equal intervals (i.e., the division based on the median). The time interval is chosen randomly on the basis that the new structure S^* should not become greater than the final S^F according to one of its values: $s^*_k \leq s^F_K, \quad \forall k \in [1, P]$. The values of the parameters of the newly formed time intervals are calculated for the parent: the size of the population of the first time interval is equal to the size in the middle of the parental time interval, and the parameters of the second time interval are equal to the population size at the end, with the time of both intervals equal to half of the parental time, while migration between populations and the demographic dynamics of population size change remain the same. In essence, the demographic model has more parameters after its structure is increased, however the history and likelihood remain the same.

Results

We implemented the method described above in the program GADMA (Genetic Algorithm for Demographic Model Analysis), written in the Python programming language. We explored the efficiency of our method using simulated data and several previously published data sets. It is important to note that tests using simulated AFS data should be interpreted with caution, these analyses and their associated demographic models are simplified for the sake of computational efficiency and additional tests must be performed. For previously published data sets, GADMA was used to first infer demographic models for two and three modern human populations using the data set analyzed by Gutenkunst et al., (2009) [11]. For these analyses, different parameter values within GADMA were examined, including the initial structure of the demographic models and using either ∂a∂i or *moments* to infer the optimal model of demographic history. The case of two human populations when initial structure and the final structure are not equal corresponds to the usage of the increase of the model complexity feature that improve the result by finding simple models first and detail them later. We then inferred demographic models for the history of two populations of the Gillette's checkerspot butterfly, *Euphydryas gillettii*, and these were compared to the previous models reported by McCoy et al., (2014) [31]. Lastly, we used GADMA to reconstruct the demographic history of the Gaboon forest frog, *Scotobleps gabonicus*, which occurs in Central and Western African rainforests, based on a data set generated and originally reported by Portik et al. (2017) [32].

Tests on the simulated AFS data

In order to demonstrate that GADMA is computationally and statistically efficient, three datasets were simulated with *moments* using the following sudden population size dynamics (all parameter values are presented in Tables S6-S8):

i. Bottleneck model for one population (4 parameters),
ii. Simple ancestral population division with asymmetric migration between two descendant populations (5 parameters),
iii. Secondary contact with symmetrical migration for three populations following split of one the two descendant populations (8 parameters).

All simulated AFS were unfolded with a size of 20 chromosomes per population. Powell's method was chosen as local search algorithm. All runs were repeated 50 times for one and two populations and 10 times for three populations.

GADMA was compared with two methods: 1) local searches starting from different initial values, and 2) using the *∂a∂i pipeline*, which was readjusted for *moments* usage. The number of initial parameters for the first method and the number of replicates in the *∂a∂i pipeline* were selected so that the mean number of fitness function evaluations was almost the same as in GADMA. For example, in case of one population: 40 initial points for the local search and five rounds of 10, 10, 10, 10 and 20 replicates for the *∂a∂i pipeline*. For the comparison, GADMA was launched for the same demographic models as the local search approaches. But two additional GADMA variations were included: one with all parameters, but with fixed dynamics of population sizes (sudden), and a second with variation of these dynamics. Therefore, five different optimizations were compared and the results including parameters values, maximum, mean and standard deviation of log-likelihood are provided in Tables S6-S8.

For one population, 50 runs of the *∂a∂i pipeline* showed the best demographic model compared to the other methods. Although, the best model obtained using GADMA had slightly higher likelihood score, GADMA was better on average. During demographic inferences without limitations on the population dynamics, the known problem about the uncertainties of the AFS appeared: the model with an early exponential bottleneck has almost the same likelihood as the model with sudden population size changes. All three versions of GADMA showed nearly identical results with the better mean and standard deviation of the likelihood score compared to local search and *∂a∂i pipeline*.

For two populations, all methods were comparable in terms of the best model inferred after 50 replicate runs. All optimizations except the GADMA without limitation on size dynamics were comparable in terms of mean and variation of likelihood. The local search from different initial points and GADMA with prior knowledge about model were both able to infer the true model. Two additional simulations with GADMA also showed close to the optimum demographic models and the optimization without limitation on size dynamics inferred sudden changes as expected.

For three populations GADMA with presized model received the best maximum, mean and variation of likelihood among all observed methods. Both additional optimizations without prior demographic model knowledge showed symmetric migrations and all extra parameters close to true values. GADMA without limitation on size change dynamics inferred not zero migration after split of ancestral population, however, we assume that this could be because not enough runs were performed. All inferred dynamics in spite of the fact that not all of them are sudden showed the constancy of size change during time intervals. Thus we could argue that GADMA could infer the true demographic history without any knowledge of its model.

Testing the human Out of Africa model with GADMA

One of the most popular demographic history models for human populations is the so-called "Out of Africa" model, which consists of three populations [11, 12, 33, 34]:

- YRI — Yoruba individuals from Ibadan,
- CEU — Utah residents with ancestry from northern and western Europe,
- CHB — Han Chinese individuals from Beijing.

In order to demonstrate the effectiveness of our method, we choose to use the allele frequency spectrum from the paper of Gutenkunst et al, (2009) [11], in which the ∂a∂i method is introduced and the demographic history models for two (YRI, CEU) and three (YRI, CEU, CHB) populations are inferred from this spectrum. These models (Figure 10a and Figure 11a) were obtained from a large number of local optimization launches and also have a number of restrictions on the number of population parameters: the size of the YRI population does not change after the first expansion of the ancestral population, migrations are symmetrical and the dynamics of population size change are fixed as sudden, except for the last time interval for CEU and CHB, where exponential growth occurs. The model for two populations has a total of 6 parameters (Table 2) whereas the model for three populations has a total of 14 (Table 4).

The $21 \times 21 \times 21$ AFS was constructed in Gutenkunst et al., (2009) [11] on the basis of the Environmental Genome Project [35]. All biallelic SNVs from non-coding regions of 219 genes (totaling 5.01 Mb) were considered and the effective length of the used sequence was equal to $L = 4.04 \times 10^6$. We used the same neutral mutation rate equal to $\mu = 2.35 \times 10^{-8}$ per site per generation and the same generation time equal to $T_g = 25$ years as in Gutenkunst et al., (2009). Thus, the average frequency of mutation in one individual per generation is equal to $\theta_0 = 4\mu L = 0.37976$.

The following parameters of the genetic algorithm were used: the size of the generation of the demographic models was chosen to be equal to 10, the strength and mutation rate was equal to 0.2, and the proportions of the best, mutated, crossed and random models in the new generation were 0.2 : 0.3 : 0.3 : 0.2. The strength and rate of mutation were adaptive with the constants of 1.05 and 1.02, respectively. The AFS was simulated using ∂a∂i with a $G = \{40, 50, 60\}$ grid size, the value of the likelihood was considered significant to the second decimal point, and the genetic algorithm stopped after 100 iterations without improvement. As for the local optimization search, the Powell method was chosen.

For the human population data, we used the block bootstrapped data set from Gutenkunst et al. 2009, where it was done over 219 sequenced loci under the assumption that the loci are well separated and can be treated as independent. The confidence intervals reported in Table 2 and Table 4 were calculated as $\overline{\theta^*} \pm \sigma(\theta^*)$, where θ^* denotes the maximum likelihood values of parameters, $\overline{\theta^*}$ and $\sigma(\theta^*)$ — the mean and standard deviation of the bootstrap results. All our model parameters are positive by definition, so their logarithms were used to calculate confidence intervals. In case of three human population confidence intervals are different to those from the original paper Gutenkunst et al., (2009). However this fact popped out in our analysis and we argue that is because of different way of optimization: for each bootstrapped data only one local optimization with wider search intervals for parameters values was launched from found optimum point.

The YRI, CEU two population example

Three demographic models were inferred from the same AFS: using the same parameters as in Gutenkunst et al. (2009) [11] (model 1) and two with all possible nine parameters (models 2, 3) but with different initial demographic model structures. Model 2 had a structure (1,1), which then expanded to (2,1) during the GADMA run, whereas model 3 had an initial structure of (2,1). We ran GADMA 10 times for each of the three models (Table 2, Figure 7). All three models resulted in likelihoods better than the final demographic model originally inferred in Gutenkunst et al., (2009). The parameters of models 1 and 2 are not significantly different, and model 3 has the best likelihood value and *CLAIC* score. Model 3 shows a lower population size of Europeans, a larger rate in their growth (from 25 individuals to 9 thousand) and a shorter separation time than the best model found in Gutenkunst et al., (2009) as well as models 1 and 2. Migration rates between the populations were chosen to be asymmetric, but they are largely equal to each other and coincide with values among the three models.

To demonstrate the inefficiency of the methods of local optimization for the model from Gutenkunst et al., (2009) [11], one of the methods proposed by ∂a∂i, BFGS, was launched 100 times. In each run, the initial parameters values were chosen randomly. The best value of the log-likelihood was −1629.24, which is quite far from the optimal value of −1066.35 reported in Gutenkunst et al., (2009). The average time of one optimization run was about 21 minutes. We then used a more efficient local optimization method implemented in the *dadi-pipeline* developed by Portik et al., (2017) [32]. It implements a scheme of sequential runs using the Nelder-Mead local optimization with initial random parameter values and perturbation of values between runs. We used the following settings of the *dadi-pipeline* tool: three rounds with 10, 20, 50 replications with 3-, 2-, and 1-fold perturbations, respectively, for each round. The *dadi-pipeline* was launched 50 times and the best resulting model had a log-likelihood equal to −1073.98. The average run time for one launch of optimization was approximately 10 minutes.

Table 1. Comparison of runs using GADMA with different initial demographic model structures: simple (10 times) and complex (10 times) for the case of searching for the optimum demographic models for two human populations, YRI and CEU.

	Initial struct.	Mean T of one it. (sec)	Mean num. of iterations	Mean $\log LL$	Std $\log LL$
Model 2	(1,1)	4.06	2938	**−1066.39**	**0.22**
Model 3	(2,1)	**3.77**	**1400**	−1071.16	14.35

$\log LL$ — log likelihood.

To compare the runs with different initial demographic model structures (models 2 and 3), characteristics such as time for one iteration of the genetic algorithm, number of iterations, and mean and standard deviation of the log-likelihood value were calculated (Table 1). Launches with a simple initial model structure show a more stable result in terms of the likelihood value but they have a longer average run time for one iteration. Furthermore, all the models obtained for simple-structure launches have the same demographic dynamics of population size change and similar parameters as the final model reported in Gutenkunst et al., (2009), which is incorrect in cases involving launches of complex-structure models, as the best model shows a linear growth of the European (CEU) population as opposed to exponential growth in other cases. At the same time, although the launches of models with complex model structures result in a final model with a better log-likelihood score, it differs from the other models in terms of parameters values, which may indicate that it is inaccurate.

The YRI, CEU, CHB three population example

We also applied GADMA to infer demographic models for the case of three human populations based on same the AFS used in Gutenkunst et al., (2009) [11]. The first model (model 1) used the same parameters as in Gutenkunst et al., (2009), and their corresponding values were inferred (Table 4). GADMA yielded

Figure 7. (a) Demographic model of two human populations (YRI and CEU) inferred using ∂a∂i, as originally reported by Gutenkunst ey al., (2009). This model has 6 parameters. The size of the African population after its growth 169,000 years ago is constant and migration rates between African and European populations are symmetric. (b) The demographic model from the same allele frequency spectrum inferred using GADMA with 12 parameters. Note that the differences between the inferred models is the slightly later age of the split between the YRI and CEU populations and the linear population growth (as opposed to exponential growth) in the CEU population in the model obtained with GADMA.

Table 2. Maximum likelihood parameters for different demographic models for two human populations (YRI and CEU) inferred using either ∂a∂i or GADMA (the latter under three different parameter settings). The migration rates are per generation. 95% confidence intervals are indicated in brackets.

	Model from ∂a∂i example		Model from GADMA (1)		Model from GADMA (2)		Model from GADMA (3)	
Param. number:	6		6		8		8	
Log-likelihood:	−1066.35		−1066.28		−1065.87		**−1065.15**	
CLAIC (*eps* = 10^{-5})	35059.26		33702.64		**28389.58**		53101.50	
Parameters:								
N_A	7240	[6841 – 9166]	7230	[6840 – 9167]	7200	[6920 – 7519]	7210	[6561 – 8914]
N_{AF0}	13620	[11575 – 16177]	13580	[11574 – 16180]	13400	[11331 – 18032]	14000	[10682 – 25283]
N_{EU0}	515	[383 – 809]	530	[383 – 810]	560	[415 – 673]	25	[14 – 170]
N_{EU}	13360^e	[7320 – 18892]	12400^e	[7314 – 18906]	12100^e	[9171 – 18576]	8950^l	[5985 – 10623]
N_{AF}	(=N_{AF0})		(=N_{AF})		13500	[11698 – 14834]	13300	[10221 – 15611]
$m_{AF-EU}(\times 10^{-5})$	6.3	[4.5 – 9.6]	6.3	[4.5 – 9.6]	7.1	[1.06 – 1.93]	5.8	[4.0 – 11.3]
$m_{EU-AF}(\times 10^{-5})$	(=m_{AF-EU})		(=m_{AF-EU})		6.1	[9.47 – 14.8]	6.1	[4.1 – 9.7]
T_{AF} (kya)	168.5	[87.3 – 165.8]	171.5	[87.3 – 165.9]	176.5	[122.1 – 230.6]	171.1	[75.1 – 224.2]
T_{AF-EU} (kya)	40	[30.8 – 58.5]	40.8	[30.8 – 58.6]	42.3	[35.3 – 46.7]	34.8	[29.1 – 47.6]

^{e}Exponential growth
^{l}Linear growth
N_A — size of ancestral population; N_{AF0} — size of ancestral population after growth; N_{EU0} — size of European population after divergence of ancestral population; N_{EU} — current size of European population (after growth); N_{AF} — current size of African population; m_{AF-EU} — migration rate from European pop. to African pop.; m_{EU-AF} — migration rate from African pop. to European pop.; T_{AF} — time of ancestral population size growth; T_{AF-EU} — time of divergence.

better log-likelihood values for parameters than those reported in Gutenkunst et al., (2009). However, the timing of the split between the YRI population and the CEU+CHB populations was dated to 400,000 years ago, which is 250 thousand years older than that inferred in any previous studies. To correct this, we restricted the age of this splitting event to 150,000 years ago based on previously published estimates [36, 37, 38]. A demographic model (model 2) was inferred with this age restriction, which yielded a better log-likelihood value than that in Gutenkunst et al., (2009). Next, we inferred the demographic model (model 3) that included all 20 parameters. Here we also observed an unrealistic earlier age for the divergence between YRI and CEU+CHB (results not shown). When we applied the 150, 000 year age constraint as in model 2, we inferred a demographic model (model 3) that not only showed the highest log-likelihood value, but also the best *CLAIC* value.

As in the case of the two human population example, we also tested the three population case with the BFGS local optimization used in ∂a∂i. We launched the optimization 100 times from randomly selected initial parameters and the best log-likelihood value obtained was −6323.99, which slightly differs from the optimal log-likelihood −6316.89, which is much less than that from the comparison for two populations.

With the exception of the earlier age of divergence between the YRI and CEU+CHB populations in model 1, demographic models 1 and 2 and the one inferred by Gutenkunst et al., (2009) have similar log-likelihood values and parameter estimates. In model 3, which has 20 parameters and the best *CLAIC* value, some parameters are also quite similar to the values in the other two models. The major exceptions, however, are the inferred migration rates and population size of the Eurasian population, which exponentially grows from 200 to 1500 individuals after the split of the ancestral population. For comparison, in other models this number is a constant equal to 2,000 individuals, which seems less realistic than exponential growth. Migration rates vary considerably: they are higher in model 3 compared to models 1 and 2 or the one found by Gutenkunst et al., (2009). Model 3 shows that the largest migration occurred between the YRI and CEU populations, and following the ancestral division, between the CEU and CHB populations. Moreover, the more geographically distant the populations are, the smaller is the observed migration rate.

Table 3. Comparison of outputs from different GADMA runs using either ∂a∂i or *moments* for inferring demographic models for the YRI, CEU, CHB human populations with 20 parameters. For the runs performed with ∂a∂i, the log-likelihood value was calculated using a $G = \{40, 50, 60\}$ grid size.

	Mean time for one iter. (sec)	Mean num. of iterations	Mean $\log LL$	Std $\log LL$
∂a∂i	136.81	5531	**−6293.56**	**7.26**
moments	**18.44**	**4700**	−6305.21	12.72

$\log LL$ — log-likelihood.

GADMA was launched 10 times for each of the three models using ∂a∂i and the best solutions were observed (Table 2). We also launched GADMA using *moments* 10 times to compare its effectiveness with ∂a∂i. The authors of *moments* conducted similar comparisons on simulated data [12]. Various characteristics of run time and stability of the log-likelihood value based on the results of 20 GADMA runs (10 using ∂a∂i, 10 using *moments*) are presented in the Table 3. Log-likelihood values of models inferred using moments were recalculated using ∂a∂i with the $G = \{40, 50, 60\}$ grid size simulated AFS so that log-likelihoods obtained with the two methods were comparable. Analyses using moments were 7.5 times faster than ∂a∂i,

Figure 8. (a) Demographic model of three human populations (YRI, CEU, CHB) inferred using ∂a∂i, as originally reported by Gutenkunst ey al., (2009), which included 14 parameters. (b) The demographic model from the same allele frequency spectrum inferred using GADMA with 23 parameters.

Table 4. Maximum likelihood parameters inferred from the demographic models for the for YRI, CEU, CHB human populations. Migration rates are per generation. 95% confidence intervals are indicated in brackets.

	Model from Gutenkunst		Model from GADMA (1)		Model from GADMA (2)		Model from GADMA (3)	
Param. number:	13		13		13		20	
Log-likelihood:	−6316.89		−6314.40		−6315.86		**−6288.36**	
CLAIC ($\epsilon = 10^{-4}$)	43865.22		45182.80		46161.50		**42761.68**	
Parameters:								
N_A	7300	[6065 – 8185]	6000	[4841 – 11781]	7300	[6084 – 11227]	7300	[6713 – 10696]
N_{AF0}	12300	[10177 – 13668]	11840	[10126 – 13750]	12200	[10152 – 13862]	9900	[3837 – 17433]
N_{B0}	2100	[1667 – 2299]	2050	[1651 – 2276]	2070	[1652 – 2308]	280	[16 – 528]
N_B		$(=N_{B0})$		$(=N_{B0})$		$(=N_{B0})$	1450^e	[1127 – 1798]
N_{AF1}		$(=N_{AF0})$		$(=N_{AF0})$		$(=N_{AF0})$	14000	[10812 – 18451]
N_{AF}		$(=N_{AF0})$		$(=N_{AF0})$		$(=N_{AF0})$	11000^l	[4629 – 20567]
N_{EU0}	938	[598 – 1496]	930	[556 – 1686]	950	[543 – 1721]	890	[613 – 1164]
N_{EU}	27300^e	[16844 – 38704]	24700^e	[12232 – 48543]	23700^e	[12310 – 48914]	19600^e	[13745 – 35578]
N_{AS0}	510	[326 – 788]	500	[302 – 876]	510	[299 – 867]	560	[361 – 877]
N_{AS}	53200^e	[26001 – 91501]	50000^e	[14211 – 159816]	46200^e	[14205 – 167491]	42200^e	[18188 – 100754]
m_{AF-B} $(\times 10^{-5})$	25	[22.3 – 33.8]	26.2	[22.7 – 33.9]	25.4	[20.9 – 35.4]	17.0	[11.8 – 29.3]
m_{B-AF} $(\times 10^{-5})$		$(=m_{AF-B})$		$(=m_{AF-B})$		$(=m_{AF-B})$	57.4	[44.0 – 74.4]
m_{AF-EU} $(\times 10^{-5})$	3.0	[1.47 – 5.7]	3.1	[1.45 – 5.84]	3.1	[1.58 – 5.52]	4.5	[1.98 – 8.26]
m_{EU-AF} $(\times 10^{-5})$		$(=m_{AF-EU})$		$(=m_{AF-EU})$		$(=m_{AF-EU})$	2.8	[1.34 – 5.91]
m_{AF-AS} $(\times 10^{-5})$	1.9	[0.02 – 77.3]	1.9	[0.11 – 20.5]	2.0	[0.35 – 8.37]	0.9	[0.01 – 16.2]
m_{AS-AF} $(\times 10^{-5})$		$(=m_{AF-AS})$		$(=m_{AF-AS})$		$(=m_{AF-AS})$	2.1	[0.58 – 6.35]
m_{EU-AS} $(\times 10^{-5})$	9.6	[0.39 – 164.6]	10.2	[3.43 – 25.9]	10.2	[2.60 – 31.2]	18.8	[11.5 – 29.7]
m_{AS-EU} $(\times 10^{-5})$		$(=m_{AS-EU})$		$(=m_{AS-EU})$		$(=m_{AS-EU})$	6.8	[1.09 – 28.35]
T_{AF} (kya)	220.8	[249.4 – 5201.3]	570	[297.6 – 2827.9]	232	[193.3 – 2211.2]	274.8	[198.2 – 804.7]
T_B (kya)	144.0	[246.4 – 2503.7]	400	[234.2 – 2309.9]	150	[152.1 – 2218.3]	149.8	[98.9 – 666.9]
T_{EU-AS} (kya)	21.1	[17.6 – 25.1]	21.0	[17.2 – 26.4]	21.1	[17.1 – 26.4]	22.4	[18.2 – 26.9]

^{e}Exponential growth
^{l}Linear growth
N_A — size of ancestral population; N_{AF0} — size of ancestral population after growth; N_{B0} — size of CEU+CHB population after divergence of ancestral population; N_B — size of CEU+CHB population before its split; N_{AF1} — size of YRI population at the time of CEU+CHB population divergence; N_{AF} — current size of YRI population; N_{EU0} — size of CEU population after divergence; N_{EU} — current size of CEU population; N_{AS0} — size of CHB population after divergence; N_{AS} — current size of CHB population; m_{1-2} — migration rate from population 2 to population 1; T_{AF} — time of growth of size of ancestral population; T_B — time of divergence of ancestral population to YRI population and CEU+CHB population; T_{EU-AS} — time of divergence of CEU+CHB population to CEU population and CHB population.

whereas those using ∂a∂i were more accurate: the average and variance of the likelihood values of the inferred models were better than the values inferred using *moments*.

Estimation of the CLAIC for human population data

In order to compare demographic models of the human data set with different numbers of parameters, we estimated the *CLAIC* scores for each model. As the implementation from Coffman et al., 2015 [26] uses numerical methods for Hessian and gradient estimations, the gradient descent depends on the step size, denoted by epsilon (ϵ). Very small values of epsilon causes numerical issues and the analysis should be done with different epsilon values to ensure that the results are stable [R. Gutenkunst, pers. comm.]. We performed experiments to show how the value of *CLAIC* depends on the value of epsilon and on the choice of using either ∂a∂i or *moments*. The results of *CLAIC* calculation for all models (8 models for modern human data and 15 models for Gillette's checkerspot butterfly population (see below)) are presented in Table S5. The value of epsilon was taken to equal to one of 7 values: 10^{-8}, 10^{-7}, ... up to 10^{-2}. Using ∂a∂i, the most stable result was for epsilon values between 10^{-5} and 10^{-3} (for all models). The interval over which models were stable using *moments* was wider: $[10^{-6}, 10^{-2}]$. Values 10^{-8}, 10^{-7} do turn out to cause numerical issues resulting in the *CLAIC* to be around $2 \cdot logLL$ due to the small value of trace. Thus, *moments* showed very stable and reliable results for all values of epsilon except for those with small values. For ∂a∂i the results were not as reliable and we recommend to checking the *CLAIC* for different values of epsilon when ∂a∂i is used.

Demographic history of Gillette's checkerspot butterfly

We next tested GADMA using data from McCoy et al., (2013) [31], which examined the demographic history of Gillette's checkerspot butterfly (*Euphydryas gillettii*). We used the same AFS as that used in the original paper, which consisted of eight individuals from a population in Colorado (CO) and eight individuals from the native population in Wyoming (WY). For our ana that used in the original paper, which consisted of eight individuals from a population in Colorado (CO) and eight individuals from the native population in Wyoming (WY). For our analyses, we used two allele frequency spectra of size 13×13, one for synonymous SNVs only and one including all SNVs.

McCoy et al. tested three types of demographic models: 1) type A — models without migration between populations; 2) type B1 — models with unidirectional migration from CO to WY, and 3) type B2 — models with bidirectional migration between CO and WY. In the original paper, the three demographic models were tested using the AFS based on synonymous SNVs only. Model A had the best CLAIC value, so the type A demo-

Figure 9. Alternative demographic models based on an AFS for Gillette's checkerspot butterflies from Colorado (CO) and Wyoming (WY), inferred with GADMA. Models of type B2 have negligible migration rates. (a) Type A model: without migration; (b) Type B2 model with migration; (c) Type B2 model with migration; (d) Type B2 model with migration.

graphic model was inferred from the AFS using all SNVs. We used models A and B2 to infer the demographic models from both allele frequency spectra (synonymous SNVs and all SNVs) with GADMA.

Without considering migration, the models used in McCoy et al., (2013) had the following structure: there was one population of N_A size, which at some point in time divided into two subpopulations, the size of which did not change further (sudden change of population size). All parameters were calculated with respect to N_A and had the following notation: η_{WY}, η_{CO} — relative population sizes at the current time; τ_{SPLIT} — time/age of the population splitting event; and M_{WY-CO}, M_{CO-WY} — scaled migration rates. For the models we inferred using GADMA, we also included the parameter $\eta_{WY0} \in [0, 1]$ — the size of the WY population immediately after the splitting event or the fraction of the size of the ancestral population that forms the WY population. The size of CO population immediately following the splitting event is equal to $1 - \eta_{WY0}$, because $N_A = 1$ before the ancestral population splits. However, in the case when the population size change is sudden, the size of the populations following the splitting event is equal to the size at the present time.

We ran four executions of GADMA with different data inputs: 1) the AFS using synonymous SNVs only without migration; 2) the AFS using synonymous SNVs only with migration; 3) the AFS using all SNVs without migration; and 4) the AFS using all SNVs with migration. For each execution, the analysis was repeated 50 times. *moments* was used to simulate the AFS due to its faster computational speed. However, of the final log-likelihood scores that are presented in Tables S1, 5 were calculated using ∂a∂i with a $G = \{32, 42, 52\}$ grid size in order to compare our results with the original findings reported in McCoy et al., (2013).

The length of one generation of the demographic models was selected to be 10, the strength and mutation rate were set to 0.2, with constants 1.0 and 1.02, respectively, and the proportions of the best, mutated, crossed and random models in the new generation equal to 0.2 : 0.3 : 0.3 : 0.2. The likelihood was considered significant to two significant digits and the genetic algorithm stopped after 100 iterations without improvement. As for the local search, the Powell method was chosen. Since the structure of the demographic model from McCoy et al., (2013) corresponds to the simplest structure (1,1), it was chosen as both the initial and final structure of the demographic model.

The confidence intervals were calculated for all resulting models (Table S1 and Table 5) the same way as was done for the human population data. In the case of the demographic models for the Gillette's checkerspot butterfly, all our parameters are positive. McCoy et al., (2013) did not use logarithms to calculate confidence intervals; they just assumed the lower bound of the intervals to be positive. However, when we used logarithms to calculate confidence intervals, we found them to be extremely wide for migration between the two populations. Another problem we encountered was the performance associated with bootstrapping the data for the Gillette's checkerspot butterfly. The data was derived from RNA-seq data, in which different alleles are likely linked. Bootstraping should be performed over unlinked regions of the genome. McCoy et al. 2013 provide the assembly of the transcriptome, so it became possible for us to perform bootstrapping over the contigs of the assembly. This was applied to generate confidence intervals and *CLAIC* scores.

Several runs of GADMA produced different local minima and the resulting alternative models and their inferred parameters are presented in Figure 9 and Table 5, respectively. We note that one of the models selected by GADMA, Model 26 (Figure 12d), was the same model as was inferred by McCoy et al., (2013), which includes a sudden growth of population size following the splitting of the ancestral population. For the AFS based synonymous SNVs, only models of type B2 had better likelihood values than models of type A, in contrast to the models inferred by McCoy et al., (2013).

For all models, the *CLAIC* values were calculated and are

Table 5. Demographic models and their associated parameters inferred in GADMA using an allele frequency spectrum based on all SNVs for Gillette's checkerspot butterfly populations. 95% confidence intervals are indicated in brackets. The first column shows the parameters inferred by McCoy et al., (2013).

	A McCoy et al.		A		B2 (1)		B2 (2)		B2 (3)	
log *LL*	−283.53		−277.82		**−267.49**		−278.57		−282.74	
CLAIC ($\epsilon = 10^{-4}$)	1620.85		1619.03		**−354.00**		−185.48		51.85	
Parameters:										
η_{WY0}		NA	0.584	[0.359 – 0.738]	0.028	[0.0 – 0.254]	0.566	[0.463 – 0.660]		(= η_{WY})
η_{WY}	1.320	[1.085 – 1.594]	1.358	[1.152 – 1.650]	1.225	[1.052 – 1.494]	1.773^l	[1.383 – 2.379]	1.261	[1.053 – 1.559]
η_{CO}	0.173	[0.151 – 0.209]	0.089^l	[0.058 – 0.127]	0.074^l	[0.050 — 0.105]	0.105^l	[0.078 – 0.144]	0.183	[0.162 – 0.217]
m_{WY-CO}		NA		NA	1.244	[0.515 – 1.637]	0.801	[0.170 – 1.231]	0.316	[0.0 – 0.677]
m_{CO-WY}		NA		NA	0.171	[0.0 – 0.362]	0.056	[0.0 – 0.266]	0.091	[0.0 – 0.273]
τ_{SPLIT}	0.117	[0.103 – 0.141]	0.138	[0.120 – 0.172]	0.259	[0.190 – 0.312]	0.162	[0.129 – 0.206]	0.129	[0.108 – 0.162]
Time of split in generations:										
t_{SPLIT}	45	[44 – 48]	37	[28 – 45]	**33**	[28 – 41]	40	[35 – 49]	47	[44 – 54]

^{l}Linear growth

log *LL* — log-likelihood.
η_{WY0} — relative (to N_A) size of WY population after divergence of ancestral population; η_{WY} — current relative (to N_A) size of WY population; η_{CO} — current relative (to N_A) size of CO population; m_{WY-CO} — scaled (by $2N_A$) migration rate from CO population to WY population; m_{CO-WY} — scaled (by $2N_A$) migration rate from WY population to CO population; τ_{SPLIT} — scaled (by $\frac{1}{2N_A}$) time of divergence of ancestral population.

presented in the Table S5. The scores were calculated on the bootstrapped data to generate confidence intervals by sampling over the contigs of the transcriptome assembly to avoid linkage (see above). For the AFS generated from synonymous SNPs, the model without migrations showed the best *CLAIC* score. However, the model with migration, which had the best likelihood, had the best *CLAIC* score for the AFS generated from all SNPs.

One of the findings reported by McCoy et al., (2013) was that the demographic models that included migration may not be applicable when the real population history shows no evidence for migration. This conclusion was based on two factors: 1) the type A demographic model, inferred from the AFS using synonymous SNVs, had the best *AIC* score; and 2) the estimate of the splitting time of the ancestral population in models that included migration had wide 95% confidence intervals, such that the parameter boundaries included zero (see McCoy et al., (2013)). However, during our regeneration of these authors' results, we found errors associated with some of the parameters of the demographic models: the split time of the ancestral population and migrations were mixed up with each other, resulting in migrations, but not split times, having wide confidence intervals that included zero. Moreover, our analyses using GADMA showed that the 95% confidence interval for the split time of the ancestral population is rather good (e. g. [0.189 – 0.323] for the case of the best B2 model) and that the migration rates are so small that they can be considered as zero. Therefore, the best-fitting model that includes migration suggests a demographic history without migrations. The demographic models inferred by GADMA also have negligible migration values, and demographic models of type B2 (those with bidirectional migration), inferred as the best-fitting models, had better *CLAIC* values than type A models.

The average population size of butterflies from Colorado (CO) was estimated as $N_{CO} = 34$ individuals by McCoy et al., (2013). If we scale the parameters of the best-fitting model so that the average size of the CO population is equal to this value ($x \cdot (\eta_{CO} + (1 - \eta_{WY0}))/2 = N_{CO}$), then for best model we get 33.6 generations ($t_{SPLIT} = 2 \cdot x \cdot \tau_{SPLIT}$) after the division of the ancestral population, which corresponds to the actual 33 generations observed (1977-2010). Such estimates were made for all resulting models (Table 5). However, the best-fitting model had the best estimated value of split time of the ancestral population (33.6 generations).

Demographic history of the Gaboon forest frog

For our third evaluation of performance, we compared the demographic models inferred using GADMA with those inferred with the recently developed optimization method implemented in the *dadi pipeline* [32]. This pipeline uses ∂a∂i to simulate an AFS and infers parameters of the researcher-specified demographic model by several rounds of consistent runs using the Nelder-Mead local optimization method. During the first round, random parameter values are estimated and during each successive round, the best parameters from the previous rounds are used. Prior to each Nelder-Mead local optimization, current parameter values are perturbed. Portik et al., (2017) demonstrated the *dadi pipeline* using allele frequency spectra generated from the Gaboon forest frog (*Scotobleps gabonicus*) and these same data was used to perform the analyses using GADMA. Sampling included eighty-four samples from 33 localities of Lower Guinea, West Africa, which were divided into Northern and Southern populations according to hierarchical Bayesian clustering analysis of 7,633 unlinked SNPs generated using RADseq. Each Northern and Southern group was further divided into three clusters: Cameroon Volcanic Line North (CVLN) and Cameroon Volcanic Line South (CVLS) and Cross River (CrossRiver) populations for the Northern group, and North Coast, South Coast and East Gabon populations for the Southern group.

In order to perform our analyses using GADMA, three two-dimensional folded allele frequency spectra were chosen from Portik et al., (2017) [32]: 1) a 41×19 spectrum for Northern and Southern populations; 2) a 31×19 spectrum for CVLN and CVLS; and 3) a 15×31 spectrum for the CrossRiver and CVLN populations. In generating the allele frequency spectra, only a single SNP per RAD locus was kept, so loci are assumed to be independent. For each spectrum, we estimated best-fitting log-likelihood values, *AIC* scores, Akaike weights (ω_i) [39] and parameters for 14 demographic models. Two demographic models assumed no divergence between populations, while the remaining 12 models assumed a split of the ancestral population and different assumptions related to migration rates, isolation, and population size changes. All population size changes were considered to have sudden change dynamics, which we included in our analyses as well. For all models, we provide inferred parameter values in Tables S2, S3, and S4, including the value of $\theta = 4N_A\mu L$, where μ is the mutation rate per generation per site and L is effective sequence length.

The best-fitting demographic model inferred using the dadi pipeline for the Northern and Southern populations only suggests a population expansion, followed by secondary contact and symmetrical migration between populations ($\Delta AIC = 13.6$, $\omega_i = 0.99$). For the CVLN and CVLS populations, the best demographic model included population expansion, secondary contact and asymmetrical gene flow from CVLN to CVLS ($\Delta AIC = 94.2$, $\omega_i = 0.99$). Finally, two demographic models explain the AFS data equally well for the CrossRiver and CVLN populations: one with secondary contact but no population size change and asymmetrical migration from CVLN to Cross River ($\omega_i = 0.53$) and another that included an ancestral population division with continuous asymmetric gene flow ($\omega_i = 0.43$).

We inferred all 12 demographic models with divergence as in Portik et al., (2017) for each of the three allele frequency spectra with GADMA. Each of 36 runs was repeated 10 times as opposed to the 50 runs launched with the the *dadi pipeline*. We originally had planned to conduct the analyses with ∂a∂i to simulate the allele frequency spectra with the same grid size as that used by Portik et al., (2017): $G = \{40, 50, 60\}$ for the CrossRiver, CVLN, and CVLS populations; and $G = \{50, 60, 70\}$ for the Northern and Southern populations. However, we found that the optimization with ∂a∂i was unstable and therefore, we set upper bounds on population size ($12N_{ref}$) and generation time ($5N_{ref}$). Under these parameters, ∂a∂i was found to be stable, but some parameters were equal to the upper bounds and in such cases GADMA was launched 10 times for each model using *moments* with larger upper bounds ($100N_{ref}$ for population size and $10N_{ref}$ for generation time). The inferred demographic models and their estimated parameters are presented in Tables S2-S4. We report demographic models estimated with moments, and if a similar model was inferred by both ∂a∂i and *moments*, we only report the result with the best log-likelihood value.

For almost all 12 demographic scenarios, GADMA was able to infer better models in terms of both log-likelihood scores and parameters than were previously inferred using the *dadi pipeline* in Portik et al., (2017) [32]. Moreover, we obtained better alternative models for the CrossRiver, CVLN, Northern, and Southern populations. These models included an initial split of the ancestral population with ancestral asymmetric migration and a population size change ($\Delta AIC = 3.24$, $\omega_i = 0.82$) for the CrossRiver and CVLN populations; and secondary contact with asymmetric migration and population size change ($\Delta AIC = 6.82$, $\omega_i = 0.97$) for the Northern and Southern pop-

Figure 10. Demographic models with the highest log-likelihoods obtained from allele frequency spectra for different populations of the Gaboon forest frog, inferred using GADMA. (a) Demographic model inferred for the Northern, Southern populations; (b) Demographic model inferred for the CVLN and CVLS populations; and (c) Demographic model inferred for the CrossRiver and CVLN populations. The plots to the right of the demographic models show the AFS data (upper-left), AFS of the demographic model (upper-right), Anscombe residuals between model and data (lower-left) and the histogram of the Anscombe residuals (lower-right).

Table 6. Demographic models and associated parameters inferred in GADMA for three pair of populations of the Gaboon forest frog: Northern and Southern; CVLN and CVLS; CrossRiver and CVLN. LogLL — log-likelihood. Expected AFS was simulated using ∂a∂i with $G = \{50, 60, 70\}$ for Northern, Southern populations and with $G = \{40, 50, 60\}$ for other cases. See text for more details.

	Northern, Southern			CVLN, CVLS			CrossRiver, CVLN		
	Best from *dadi pipeline*[1]	Model structure (1,2)	Best from GADMA[2]	Best from *dadi pipeline*[3]	Model structure (1,2)	Best from GADMA[2]	Best from *dadi pipeline*[4]	Model structure (1,2)	Best from GADMA[2]
Param. number	7	11	9	8	11	9	6	11	9
Log-likelihood	−439.91	−402.20	**−402.00**	−463.33	−453.67	**−453.65**	−377.99	**−365.26**	−365.29
AIC score	893.81	826.40	**821.99**	942.67	**929.34**	**925.30**	769.98	752.52	**748.58**
Parameters:									
θ	573.9	134.7	107.0	572.18	134.17	145.5	287.96	250.58	251.88
ν_1^a	0.259	1.894	2.515	0.338	2.010	1.893	0.350	0.149	0.139
ν_2^a	0.247	2.634	3.339	1.331	0.840	0.880	6.746	7.034	6.899
m_{12}^a	NA	0.046	0.033	NA	0.0	NA	NA	2.510	2.639
m_{21}^a	NA	0.0	NA	NA	0.400	0.399	NA	0.001	NA
ν_1^b	3.121	13.544	17.139	4.528	13.220	12.266	$= \nu_1^a$	0.974	0.889
ν_2^b	1.291	5.584	7.057	1.193	3.620	3.349	$= \nu_2^a$	8.707	8.873
m_{12}^b	0.049	0.008	0.006	0.108	0.0419	0.046	1.272	0.507	0.556
m_{21}^b	$= m_{12}^b$	0.017	0.014	1.084	0.365	0.395	0.221	0.328	0.312
T_a	0.302	4.335	5.779	0.325	5.000	4.148	0.495	1.110	1.089
T_b	0.556	2.162	2.692	0.870	1.970	1.808	0.402	0.101	0.109

[1] Divergence, secondary contact with population size change and symmetric migration.
[2] Divergence, unidirectional migration, followed by population size growth and bidirectional asymmetric migrations.
[3] Divergence, secondary contact with no population change and asymmetric migration.
[4] Divergence, secondary contact with no population change and asymmetric migration.
$\theta = 4N_A\mu L$; ν_p^x — relative (to N_A) size of population p (1 for Northern/CVLN/CrossRiver, 2 for Southern/CVLS/CVLN) during time interval x after divergence (a — first time interval, b — second time interval); m_{pk}^x — scaled (by $2N_A$) migration rate from population k to population p during time interval x; T_x — scaled (by $\frac{1}{2N_A}$) time of time interval x.

ulations. For the third AFS of the CVLN and CVLS populations, the model that includes secondary contact with asymmetric migration and population size change was found to be superior based on comparison of the Akaike information criterion ($\Delta AIC = 87.98$, $\omega_i > 0.99$), similar to the findings by Portik et al., but received a higher log-likelihood value: −455.17 using GADMA versus −463.3 in Portik et al., (2017).

We next inferred the demographic model with a structure equal to (1,2) with GADMA (Table 6). For all three allele frequency spectra, such a model had the best log-likelihood among previously inferred models (Tables S2, S3 and S4). For the Northern and Southern populations, the demographic model with the (1,2) structure also had the best AIC score. This model showed similar features for all observed data: an interval of time after the splitting of the ancestral population with unidirectional migration, followed by a time interval with bidirectional migration and population size change. The direction of the unidirectional migration was as follows: from CVLN to CrossRiver; from CVLN to CVLS; and from Southern to Northern. We also constructed two additional demographic models based on observed features and inferred their parameters: splitting of the ancestral population with unidirectional migration followed by either symmetric or asymmetric migrations with population size change. The demographic model with unidirectional migration followed by asymmetric migration and population size change was found to be the best for all three allele frequency spectra (Table 6): Northern and Southern populations: $\Delta AIC = 4.41$, $\omega_i = 0.89$; CVLN and CVLS populations: $\Delta AIC = 1.04$, $\omega_i = 0.58$; and CrossRiver and CVLN populations: $\Delta AIC = 0.04$, $\omega_i = 0.44$. But there were several additional demographic models that explained the data equally well: the same demographic model as just described but which also included symmetric migration for CrossRiver, CVLN ($\omega_i = 0.43$) and secondary contact with asymmetric migration and population size change for CVLN and CVLS ($\omega_i = 0.34$).

Discussion

We report the development and mathematical justification of GADMA and demonstrate its effectiveness using several previously published data sets. Our method is based on the genetic algorithm and uses existing solutions from either ∂a∂i and *moments* to simulate the AFS from the proposed demographic model. GADMA is the first program that allows the automatic inference of demographic history of up to three populations from an allele frequency spectrum. Existing optimizations, implemented in either ∂a∂i and *moments*, require prior specification of demographic models to be tested and are thus inefficient in practice, given the large number of possible demographic scenarios that can be constructed for one or more populations. Our method is implemented in the GADMA software, which is openly accessible via the link `https://github.com/ctlab/GADMA`.

GADMA was shown to be efficient in performance: it was applied to both simulated data and to three empirical datasets, representing different organismal systems and associated demographic histories. The inferred demographic models had better log-likelihood scores than those reported in the original papers, which were derived from optimizations using either ∂a∂i or *moments* alone. Moreover, the demographic his-

tories inferred with GADMA were consistent with the known history of the three taxa [11, 37, 31]. We also demonstrated the stability of the search, starting with demographic models with simpler structures rather than more complex ones, which reflects the profitability of using a search scheme that includes an increase in model structure complexity. Additionally, we compared pipelines using *moments* or ∂a∂i and showed that the computational speed of *moments* was much greater than for ∂a∂i. Thus, GADMA is the first software that effectively infers a demographic model from an allele frequency spectrum with nothing required from the user, except the structure of the demographic model, which determines the extent of the model complexity and associated details.

Despite the increasing use of allele frequency spectra for inferring demographic history, there are some limitations of the informativeness of AFS with regards to historical demographic inference that have been noted (see Beichman et al., (2018) [3]). For example, previous studies have shown that the AFS of a single panmictic population can be matched to different demographic scenarios [9]. We also observed this issue in our simulated data for the demographic model that included a bottleneck event of a panmictic population. The inferred model with an earlier exponential bottleneck had almost the same likelihood score as the model with the sudden population size dynamic. We should expect the same behavior in the case of multiple populations, which requires estimating the joint AFS. Moreover Rosen et al., (2018) [40] showed the limitation of AFS based methods and their pathological behaviour. They also proved that expected AFS data from n samples under any demographic model could be generated by a piecewise-constant model with at least $2n - 1$ time intervals. This research is a complement to the paper Terhorst and Song, (2015) [41], where the minimax error of the inference of population size history was shown to be at least $\mathcal{O}(1/\log(s))$, where s is the number of segregating sites. This result means that the accuracy of demographic inference does not depend on the size of AFS but on the number of considered segregating sites. Although it was provided only for the populations that have experienced a bottleneck, the authors argue that this behaviour should be expected for the real data. We should consider described limitations of AFS and because of such behavior, the structure of the demographic model should not be very complex. We suggest to use structures no more than (2,1) and (2,1,1). This limitation can be solved by using additional information about observed populations, for example, two-locus statistics [42] or focus on IICR summary [43]. Incorporating genetic linkage information with AFS data could also improve the accuracy of inference of demographic history [42]. Including such information in the GADMA pipeline will allow the use of more complex demographic model structures in the future.

In our analyses using the AFS from three human populations (YRI, CEU and CHB), we inferred a best-fitting demographic model that showed an expansion out of Africa around 400 thousand years ago, which is not supported by previous studiess [11, 44, 45]. This can be caused by the fact that tree-based models often do not take into account processes such as admixture and introgression that can under- or overestimate population divergence times (e.g., Kamm et al., (2019) [13]). Alternatively, these differences could result from the limitation of the informativeness of the AFS or by noise in the spectrum as a result of including low-quality variant calls as AFS based methods should be highly sensitive to noise [40]. However, Gutenkunst et al., (2009) [11] noted the high quality of this data set. The demographic model inferred with GADMA using the same parameters as in Gutenkunst et al., (2009), under the assumption that expansion took place not earlier than 150 thousand years ago, resulted in parameter values similar to those reported in Gutenkunst et al., (2009). We also inferred all possible parameters, including asymmetric migration rates and different dynamics of population size changes, and obtained a demographic model with the best *CLAIC* score. With GADMA, we observed higher asymmetric migration rates and the growth of the CEU+CHB population after its split from the African population.

Allele frequency spectra of two isolated populations of Gillette's checkerspot buttery from Wyoming and Colorado showed several alternative models with values very close to the best value of the composite likelihood found by McCoy et al., (2014) [31]. All migrations that were inferred are negligible, which confirmed the isolation of the two populations. One of the inferred models is consistent with the demographic history that was estimated previously by McCoy et al., (2014). The demographic model inferred using GADMA with the best likelihood value seems to be a better model overall because, in addition to the best *CLAIC* score (for the AFS using all SNPs), it correctly inferred the timing of the population split to the actual known value of ~33 years. However, the model without migration showed the best *CLAIC* score for the AFS using all synonymous SNPs and could be the right choice too, so we suggest that further research is necessary to identify alternative models that may better fit the demographic history of these checkerspot butterfly populations.

We conducted a series of experiments for selecting demographic models for the Gaboon forest frog, also repeating the analyses performed by Portik et al., (2017) [32]. Nearly all of the 12 models inferred previously were found to be suboptimal. For two of the three population sets analyzed with GADMA, demographic models with higher log-likelihoods were chosen compared to those previously inferred. For the comparison that included the CVLN and CVLS populations, the demographic model with the highest log-likelihood was consistent with model inferred by Portik et al., 2017, but new values of parameters with better likelihood values were found. Demographic model optimization using ∂a∂i proved to be unstable, and we were consequently forced to either specify lower limits on specific parameter values or instead use *moments* for model optimization. *moments* proved to be indeed more stable in simulating the expected AFS from the demographic model. We then inferred the full parameters of the demographic model with a structure equal to (1,2) for each of three AFS datasets. For Northern and Southern populations, models using this structure resulted in higher log-likelihood scores. However, we noticed some peculiar properties in parameter values and generated new demographic models based on these properties and inferred their parameters using the three allele frequency spectra. These analyses resulted in a model with improved *AIC* scores for each of the three allele frequency spectra. This model contains divergence of the ancestral population, a time interval with unidirectional migration followed by a time interval with population size change and bidirectional asymmetric gene flow.

In this work, we focused on benchmarking the effectiveness of the genetic algorithm for demographic model inference on real data. We found that GADMA managed to infer better demographic models than those that were found in the original analyses for all tested data sets. However, we highlight two caveats that warrant further research. First, our method only used allele frequency spectrum data. Other data, such as those based on haplotypes or SNP data may prove to be more informative about demographic history, but current methods using such data are restricted to simple population size change dynamics [e.g., IBS tract method [34], DIYABC [46], MSMC [47]] whereas ∂a∂i and *moments*, which are implemented in GADMA, are less restricted in these dynamics. Second, it is possible to add other solutions like fastsimcoal2 or momi2 for simulating the expected AFS from demographic models and make compar-

isons between all methods. Also, even though we performed some analyses on simulated data that included models with known global optima, we suggest our method could be further verified through the use of additional simulated data sets.

While the optimization search implemented in GADMA is able to find demographic models with the best likelihood score and their associated parameters, it is important to minimize the number of parameters so as to avoid the possibility of overfitting the model to the AFS data. Fortunately, such a strategy is included in in GADMA using *AIC* and *CLAIC* scores: it informs the user about overfitting when the demographic model with the best likelihood score and best *AIC* (or *CLAIC*) score do not match. Additionally, GADMA can infer demographic models with all possible parameters, allowing researchers to explore additional models based on the inferred model, as we demonstrated in the case of the Gaboon forest frog. However, we note that GADMA does not sort out all possible numbers of parameters, so it is not guaranteed to find the model(s) with best *AIC* (or *CLAIC*) score(s). Moreover, we recommend performing block bootstrapping of data sets over unlinked regions of the genome for accurate estimation of the *CLAIC* score. We note, however, that detection of such regions can be difficult.

Another direction in the further development of our work is increasing the number of considered populations. Currently, GADMA can analyze up to three populations, similar to ∂a∂i. In contrast, moments can simulate allele frequency spectra for up to five populations. As the limitations of AFS based methods that were presented above are the important restrictions in increasing the number of populations and complexity of demographic models (we again propose the simplest structures then), we expect that this modification should be done in parallel with the incorporation of additional summaries of genetic data. Moreover, including the estimation of selection coefficients (e.g., Gutenkunst et al., (2009) [11]) and the development of a user-friendly interface for various types of data sets (e.g., all SNPs, synonymous SNPs only, etc.), will help to further expand the capabilities of GADMA. In this work we were focused on the application of the genetic algorithm as global optimization on the demographic inference. We have shown that effective global search is possible and we assume the existence of more powerful optimization method. So it is also possible to improve the proposed method, using another optimizations or various modifications of the genetic algorithm, for example, one that infers deliberately different demographic models [48].

Availability of source code and requirements

- Project name: GADMA
- Project home page: https://github.com/ctlab/GADMA
- RRID: SCR_017680
- biotoolsID: biotools:GADMA
- Operating system(s): Platform independent
- Programming language: Python
- Other requirements:
  - Python (2.5, 2.6, 2.7),
  - NumPy ($\geq$ 1.2.0),
  - Scipy ($\geq$ 0.6.0),
  - ∂a∂i ($\geq$ 1.7.0) or *moments* ($\geq$ 1.0.0)
- License: GNU GPL v3.

Availability of supporting data and materials

All data, parameters for GADMA runs and results are available in the Bitbucket repository [49]. The supplementary materials and the archival snapshot of code are published as GigaDB dataset [50]. Supplementary tables are presented both in PDF and tabular formats.

Declarations

Competing Interests

The authors declare that they have no competing interests.

Acknowledgements

We would like to express our sincere thanks to Maxim Buzdalov for the fruitful discussion about genetic algorithm, Victoria Efremova for the proofreading of GADMA manual, Nikita Alexeev and Viacheslav Borovitskiy for the statistical assistance, Ryan Gutenkunst, Rajiv Mcoy and Daniel Portik for the provided AFS data and useful discussions about their papers. Finally, we thank both reviewers Lounès Chikhi and Ryan Gutenkunst for providing excellent comments that improved the manuscript. This work was financially supported by JetBrains Research and by the Government of the Russian Federation (Grant 08-08).

References

1. Pool JE, Hellmann I, Jensen JD, Nielsen R. Population genetic inference from genomic sequence variation. Genome research 2010;20(3):291–300.
2. Schraiber JG, Akey JM. Methods and models for unravelling human evolutionary history. Nature Reviews Genetics 2015 11;16:727 EP –. http://dx.doi.org/10.1038/nrg4005.
3. Beichman AC, Huerta-Sanchez E, Lohmueller KE. Using Genomic Data to Infer Historic Population Dynamics of Nonmodel Organisms. Annual Review of Ecology, Evolution, and Systematics 2018;49:433–456.
4. Fisher RA. XVII.—The Distribution of Gene Ratios for Rare Mutations. Proceedings of the Royal Society of Edinburgh 1931;50:204–219.
5. Marth GT, Czabarka E, Murvai J, Sherry ST. The allele frequency spectrum in genome-wide human variation data reveals signals of differential demographic history in three large world populations. Genetics 2004;166(1):351–372.
6. Adams AM, Hudson RR. Maximum-likelihood estimation of demographic parameters using the frequency spectrum of unlinked single-nucleotide polymorphisms. Genetics 2004;168(3):1699–1712.
7. Voight BF, Adams AM, Frisse LA, Qian Y, Hudson RR, Di Rienzo A. Interrogating multiple aspects of variation in a full resequencing data set to infer human population size changes. Proceedings of the National Academy of Sciences of the United States of America 2005;102(51):18508–18513.
8. Chen H, Green RE, Pääbo S, Slatkin M. The joint allele-frequency spectrum in closely related species. Genetics 2007;177(1):387–398.
9. Myers S, Fefferman C, Patterson N. Can one learn history from the allelic spectrum? Theoretical Population Biology 2008;73(3):342–348.
10. Excoffier L, Foll M. Fastsimcoal: a continuous-time coalescent simulator of genomic diversity under arbi-

trarily complex evolutionary scenarios. Bioinformatics 2011;27(9):1332–1334.
11. Gutenkunst RN, Hernandez RD, Williamson SH, Bustamante CD. Inferring the joint demographic history of multiple populations from multidimensional SNP frequency data. PLoS Genetics 2009;5(10):e1000695.
12. Jouganous J, Long W, Ragsdale AP, Gravel S. Inferring the Joint Demographic History of Multiple Populations: Beyond the Diffusion Approximation. Genetics 2017;206(3):1549–1567. http://www.genetics.org/content/206/3/1549.
13. Kamm J, Terhorst J, Durbin R, Song YS. Efficiently inferring the demographic history of many populations with allele count data. Journal of the American Statistical Association 2019;(just-accepted):1–42.
14. Moran PAP. Random processes in genetics. In: Mathematical Proceedings of the Cambridge Philosophical Society, vol. 54 Cambridge University Press; 1958. p. 60–71.
15. Broyden CG. The convergence of a class of double-rank minimization algorithms: 2. The new algorithm. IMA Journal of Applied Mathematics 1970;6(3):222–231.
16. Fletcher R. A new approach to variable metric algorithms. The Computer Journal 1970;13(3):317–322.
17. Goldfarb D. A family of variable-metric methods derived by variational means. Mathematics of Computation 1970;24(109):23–26.
18. Shanno DF. Conditioning of quasi-Newton methods for function minimization. Mathematics of Computation 1970;24(111):647–656.
19. Nelder JA, Mead R. A simplex method for function minimization. The Computer Journal 1965;7(4):308–313.
20. Powell MJ. An efficient method for finding the minimum of a function of several variables without calculating derivatives. The Computer Journal 1964;7(2):155–162.
21. Holland J. Adaptation in natural and artificial systems: an introductory analysis with application to biology. Control and Artificial Intelligence 1975;.
22. Zwickl DJ. Genetic algorithm approaches for the phylogenetic analysis of large biological sequence datasets under the maximum likelihood criterion. PhD thesis; 2006.
23. Gao N, Yang N, Tang J. Ancestral genome inference using a genetic algorithm approach. PloS One 2013;8(5):e62156.
24. Hamblin S. On the practical usage of genetic algorithms in ecology and evolution. Methods in Ecology and Evolution 2013;4(2):184–194.
25. Akaike H. A new look at the statistical model identification. IEEE Transactions on Automatic Control 1974;19(6):716–723.
26. Coffman AJ, Hsieh PH, Gravel S, Gutenkunst RN. Computationally efficient composite likelihood statistics for demographic inference. Molecular Biology and Evolution 2015;33(2):591–593.
27. Varin C, Vidoni P. A note on composite likelihood inference and model selection. Biometrika 2005;92(3):519–528.
28. Gravel S. Population genetics models of local ancestry. Genetics 2012;191(2):607–619.
29. Schumer M, Steiglitz K. Adaptive step size random search. IEEE Transactions on Automatic Control 1968;13(3):270–276.
30. Ruder S. An overview of gradient descent optimization algorithms. arXiv preprint arXiv:160904747 2017;.
31. McCoy RC, Garud NR, Kelley JL, Boggs CL, Petrov DA. Genomic inference accurately predicts the timing and severity of a recent bottleneck in a nonmodel insect population. Molecular Ecology 2014;23(1):136–150.
32. Portik DM, Leaché AD, Rivera D, Barej MF, Burger M, Hirschfeld M, et al. Evaluating mechanisms of diversification in a Guineo-Congolian tropical forest frog using demographic model selection. Molecular Ecology 2017;26(19):5245–5263.
33. Gravel S, Henn BM, Gutenkunst RN, Indap AR, Marth GT, Clark AG, et al. Demographic history and rare allele sharing among human populations. Proceedings of the National Academy of Sciences 2011;108(29):11983–11988.
34. Harris K, Nielsen R. Inferring demographic history from a spectrum of shared haplotype lengths. PLoS Genetics 2013;9(6):e1003521.
35. Sharp RR, Barrett CJ. The environmental genome project: ethical, legal, and social implications. Environmental Health Perspectives 2000;108(4):279.
36. Cruciani F, Trombetta B, Massaia A, Destro-Bisol G, Sellitto D, Scozzari R. A revised root for the human Y chromosomal phylogenetic tree: the origin of patrilineal diversity in Africa. The American Journal of Human Genetics 2011;88(6):814–818.
37. Poznik GD, Henn BM, Yee MC, Sliwerska E, Euskirchen GM, Lin AA, et al. Sequencing Y chromosomes resolves discrepancy in time to common ancestor of males versus females. Science 2013;341(6145):562–565.
38. Shriner D, Tekola-Ayele F, Adeyemo A, Rotimi CN. Genome-wide genotype and sequence-based reconstruction of the 140,000 year history of modern human ancestry. Scientific reports 2014;4:6055.
39. Burnham KP, Anderson DR. Model selection and multimodel inference: a practical information-theoretic approach. Springer Science & Business Media; 2003.
40. Rosen Z, Bhaskar A, Roch S, Song YS. Geometry of the sample frequency spectrum and the perils of demographic inference. Genetics 2018;210(2):665–682.
41. Terhorst J, Song YS. Fundamental limits on the accuracy of demographic inference based on the sample frequency spectrum. Proceedings of the National Academy of Sciences 2015;112(25):7677–7682.
42. Ragsdale AP, Gutenkunst RN. Inferring demographic history using two-locus statistics. Genetics 2017;p. genetics–117.
43. Mazet O, Rodríguez W, Grusea S, Boitard S, Chikhi L. On the importance of being structured: instantaneous coalescence rates and human evolution—lessons for ancestral population size inference? Heredity 2016;116(4):362.
44. Scheinfeldt LB, Soi S, Tishkoff SA. Working toward a synthesis of archaeological, linguistic, and genetic data for inferring African population history. Proceedings of the National Academy of Sciences 2010;107(Supplement 2):8931–8938.
45. Shriner D, Tekola-Ayele F, Adeyemo A, Rotimi CN. Ancient human migration after Out-of-Africa. Scientific reports 2016;6:26565.
46. Cornuet JM, Pudlo P, Veyssier J, Dehne-Garcia A, Gautier M, Leblois R, et al. DIYABC v2. 0: a software to make approximate Bayesian computation inferences about population history using single nucleotide polymorphism, DNA sequence and microsatellite data. Bioinformatics 2014;30(8):1187–1189.
47. Schiffels S, Durbin R. Inferring human population size and separation history from multiple genome sequences. Nature genetics 2014;46(8):919.
48. Squillero G, Tonda A. Divergence of character and premature convergence: A survey of methodologies for promoting diversity in evolutionary optimization. Information Sciences 2016;329:782–799.
49. Noskova E, Ulyantsev V, Koepfli KP, O'Brien SJ, Dobrynin P. Data and results for "GADMA: Genetic algorithm for inferring demographic history of multiple populations from allele frequency spectrum data". Bitbucket repository;https://bitbucket.org/noscode/gadma_results.

50. Noskova E, Ulyantsev V, Koepfli KP, O'Brien SJ, Dobrynin P. Supporting data for "GADMA: Genetic algorithm for inferring demographic history of multiple populations from allele frequency spectrum data". GigaScience Database 2020;http://dx.doi.org/10.5524/100690.

Figure 1

Click here to access/download;Figure;figure_1.pdf

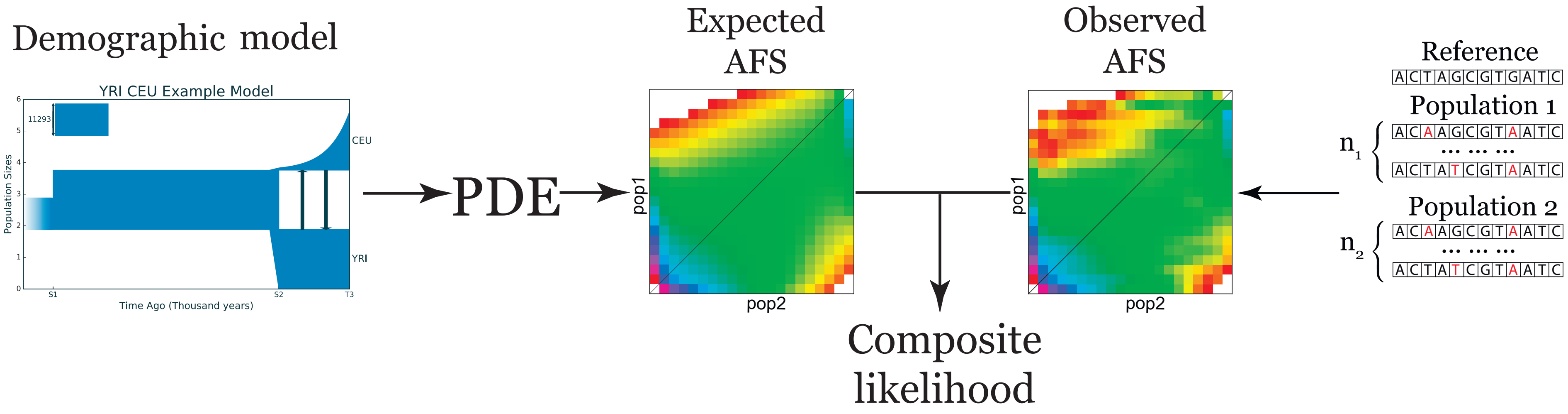

Figure 2

Click here to access/download;Figure;figure_3.pdf

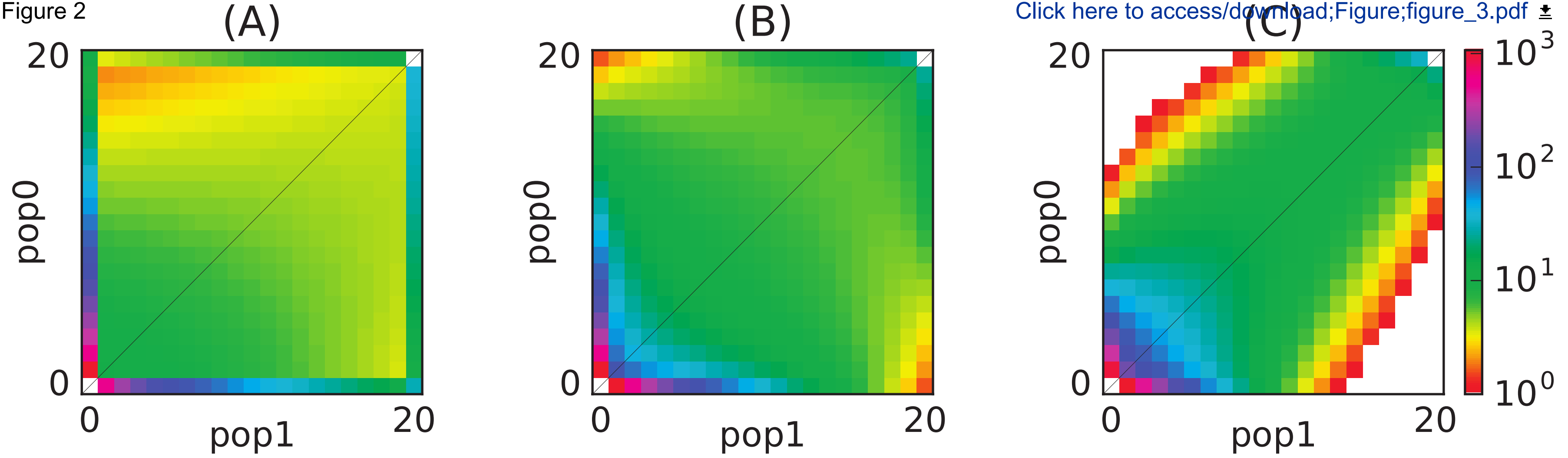

Figure 3

Click here to access/download;Figure;figure_5.pdf

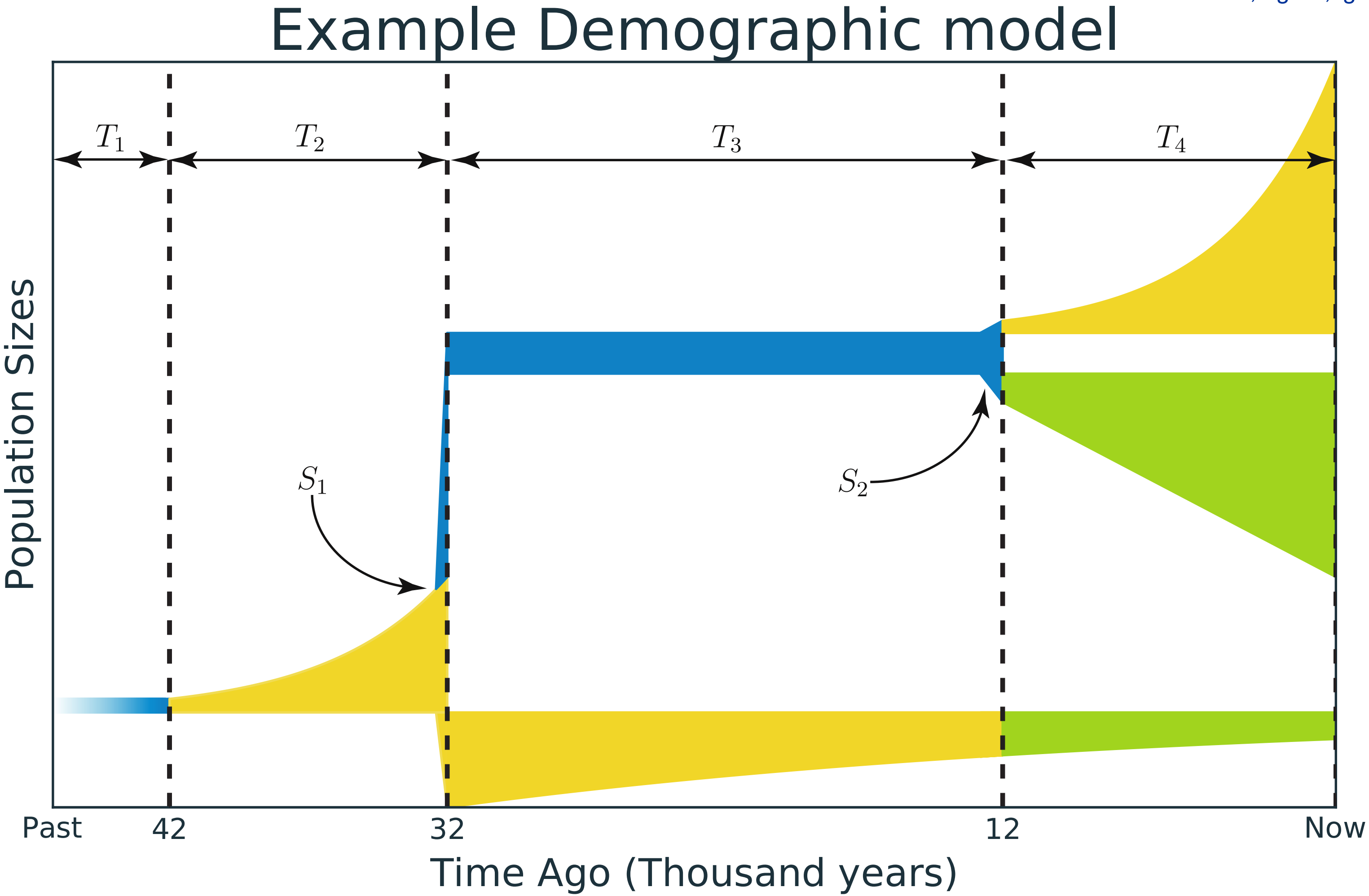

Figure 4

Click here to access/download;Figure;figure_7.png

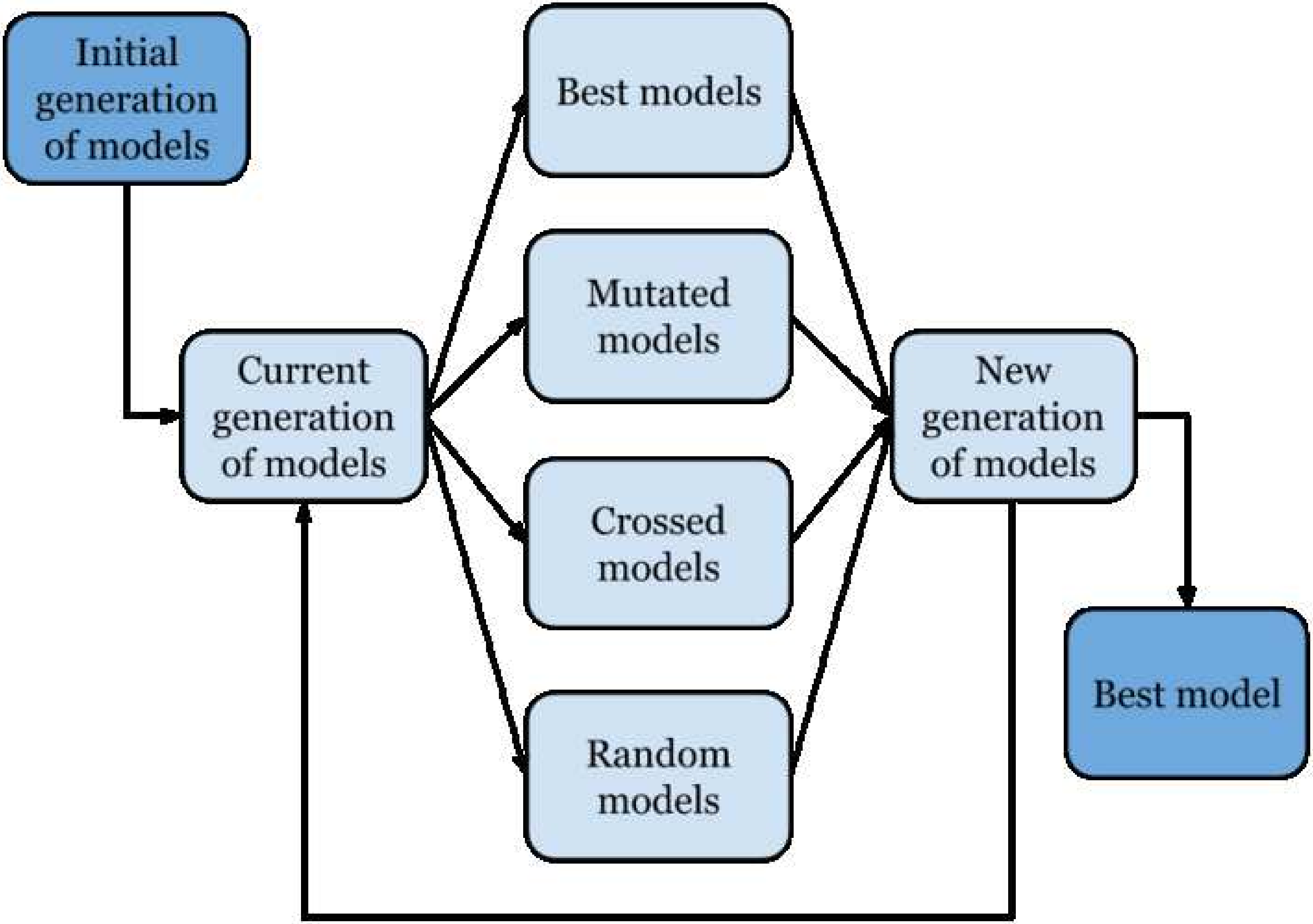

Figure 5

Click here to access/download;Figure;figure_8.pdf

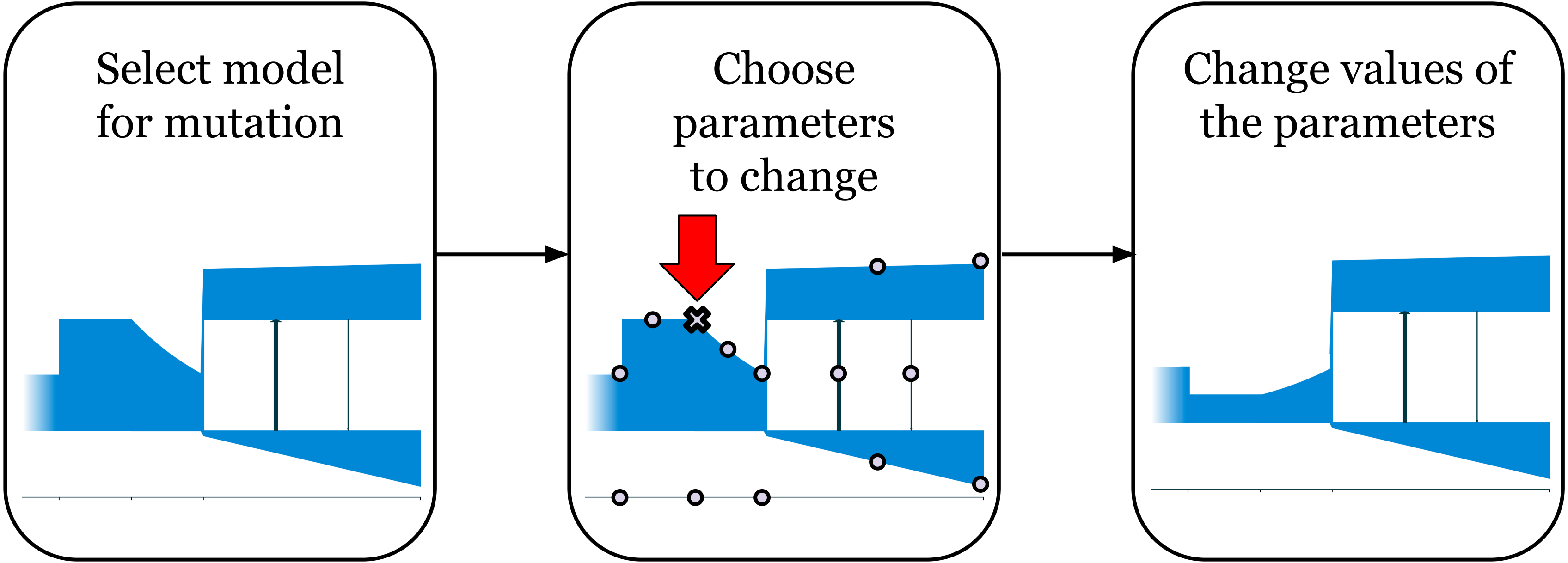

Figure 6

Click here to access/download;Figure;figure_9.pdf

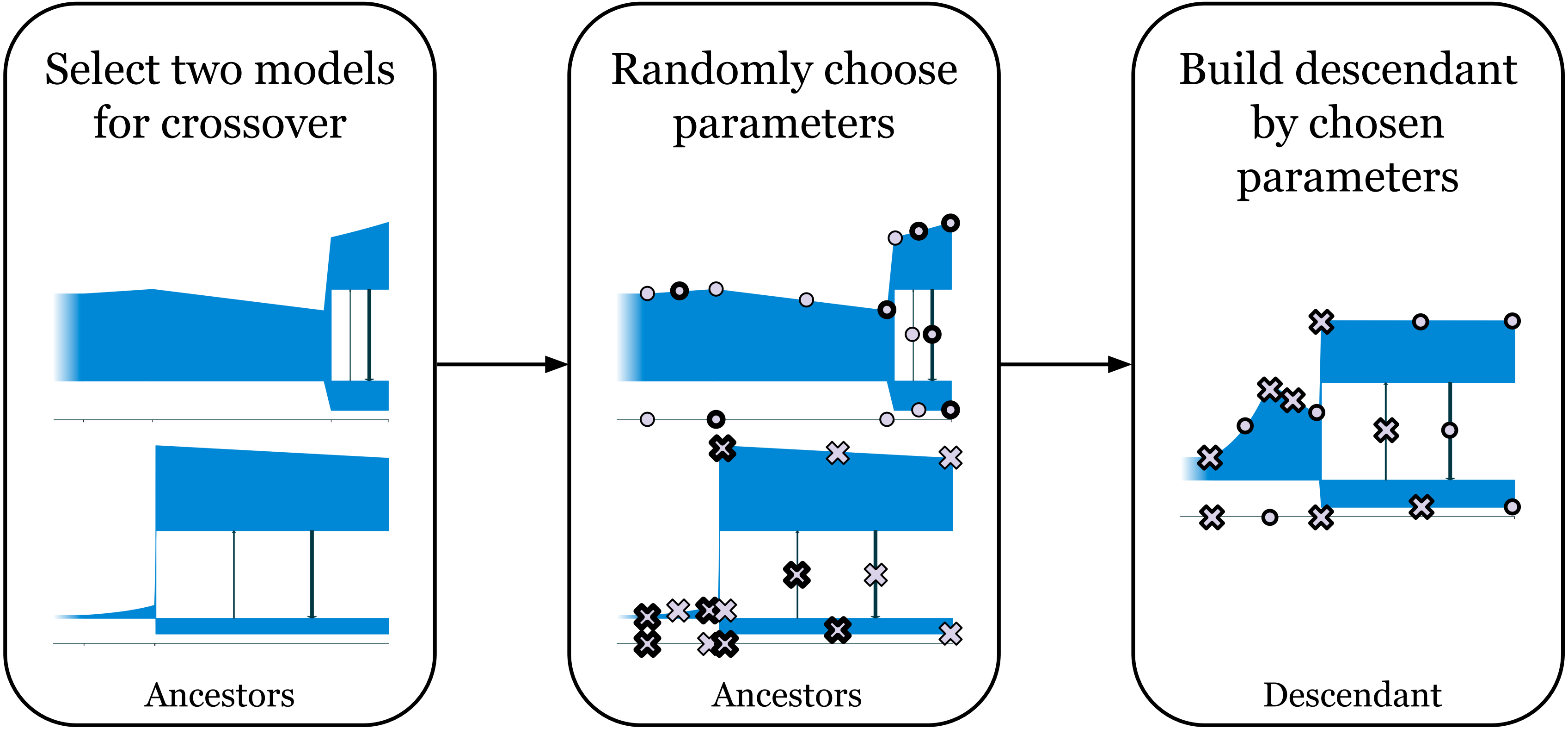

Figure 7

Click here to access/download;Figure;figure_10.pdf

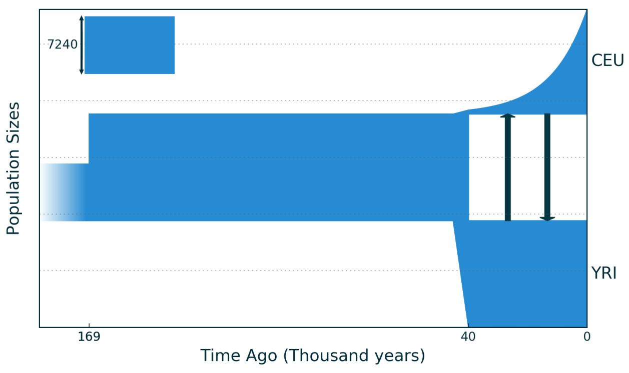

(a)

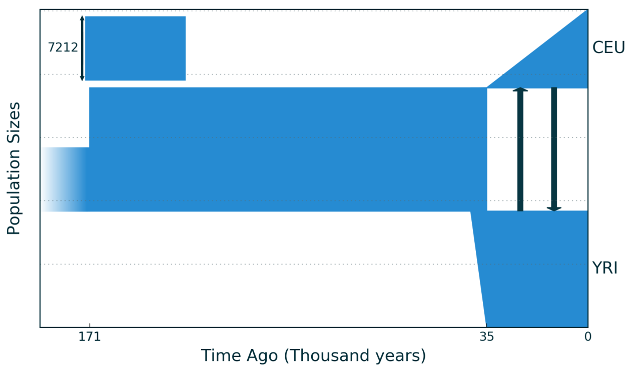

(b)

Figure 8 Click here to access/download;Figure;figure_11.pdf

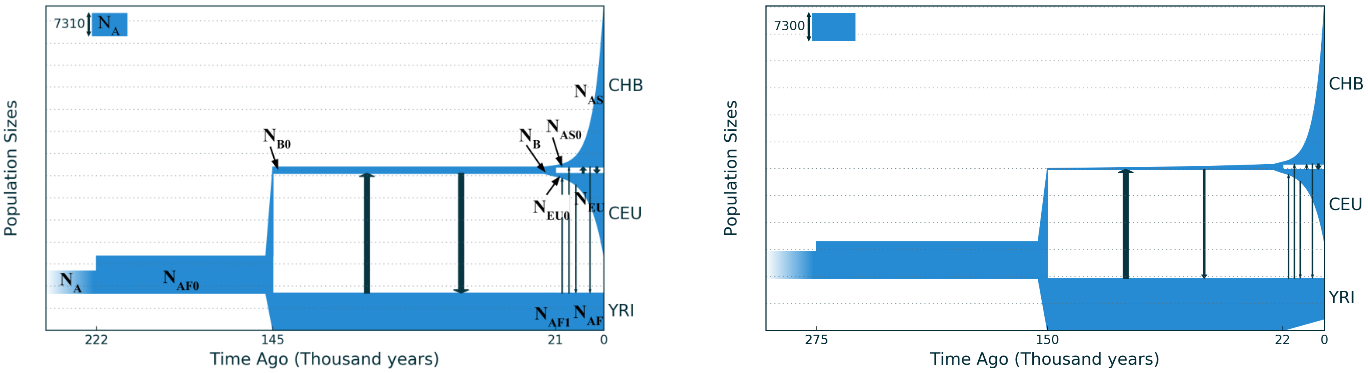

(a) (b)

Figure 9 Click here to access/download;Figure;figure_12.pdf

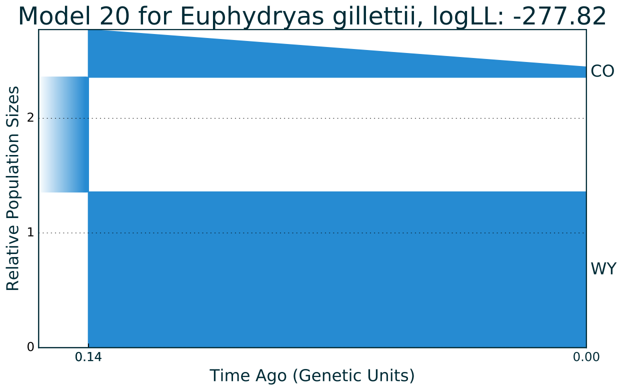

(a)

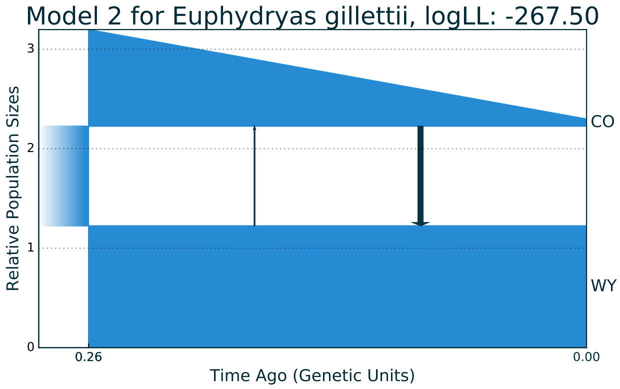

(b)

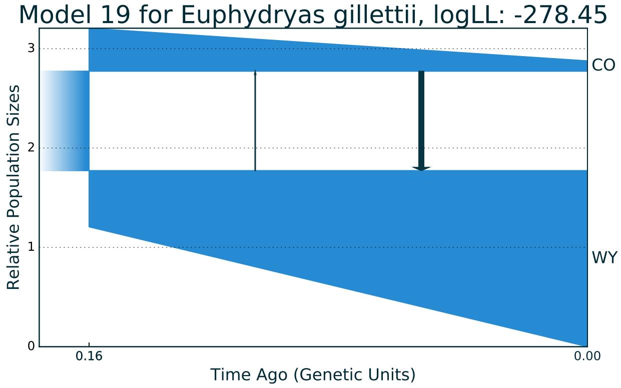

(c)

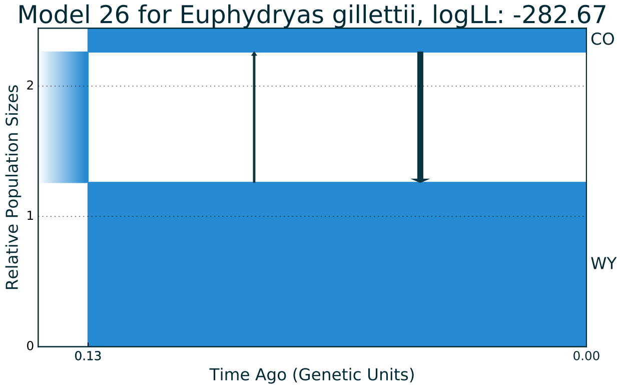

(d)

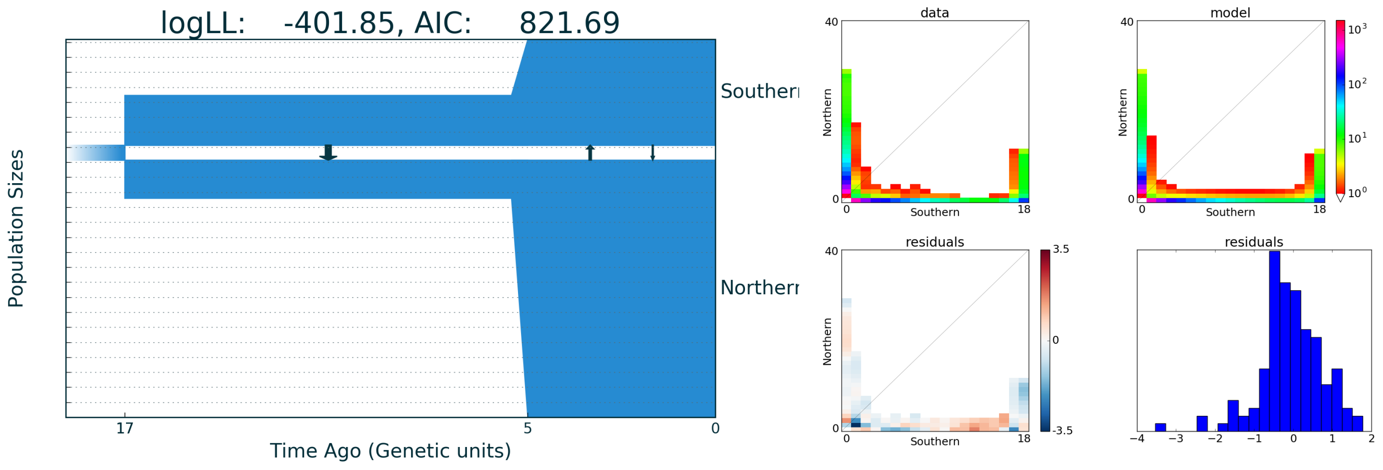
logLL: -401.85, AIC: 821.69
Southern
Northern
Population Sizes
Time Ago (Genetic units)
17
5
0
data
model
residuals
residuals
Northern
Southern

(a)

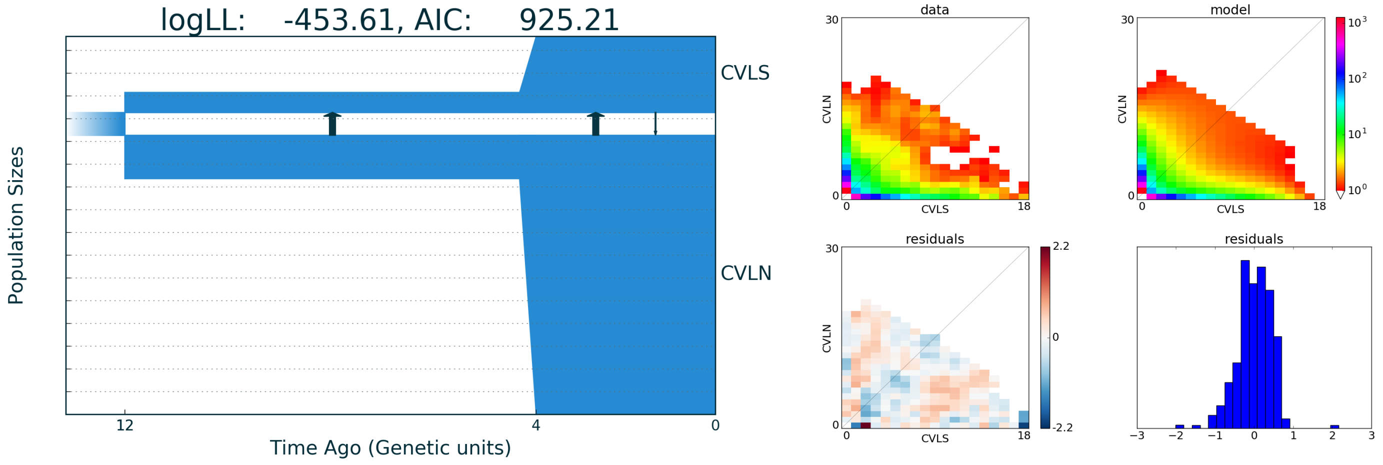
logLL: -453.61, AIC: 925.21
CVLS
CVLN
Population Sizes
Time Ago (Genetic units)
12
4
0
data
model
residuals
residuals

(b)

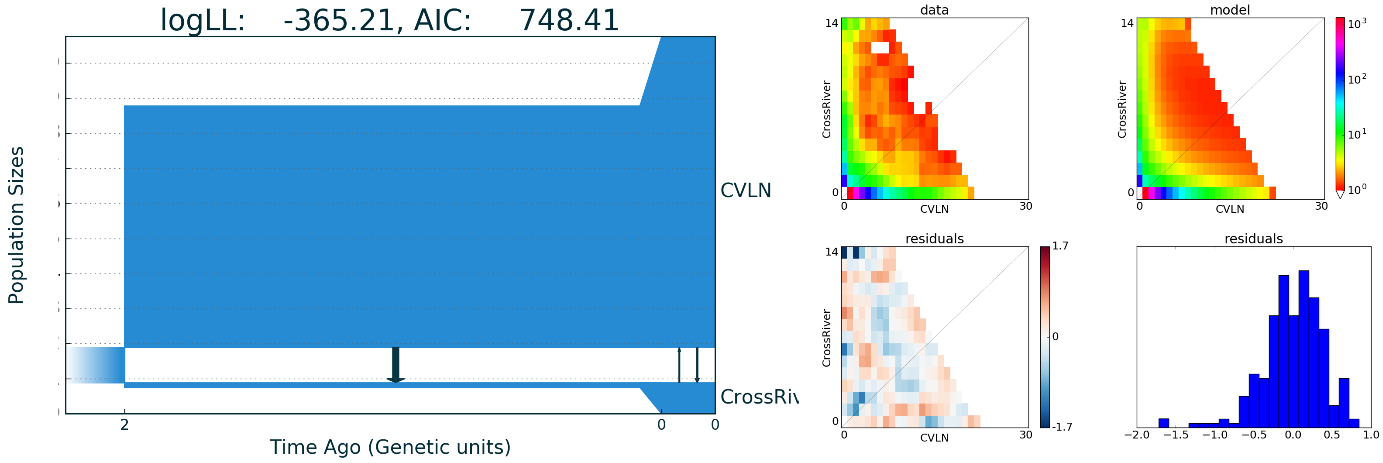
logLL: -365.21, AIC: 748.41
CVLN
CrossRiv
Population Sizes
Time Ago (Genetic units)
2
0
0
data
model
residuals
residuals
CrossRiver

(c)

Personal Cover Click here to access/download;Personal Cover;cover_letter.pdf

Ekaterina Noskova
International Laboratory "Computer Technologies"
ITMO University
49, Kronverksky prospect
197101, Saint Petersburg
Russia
ekaterina.e.noskova@gmail.com

Dr. Laurie Goodman
Editor in Chief
GigaScience

27 April 2019

Dear Dr. Goodman,

We are pleased to submit our manuscript entitled, “GADMA: Genetic algorithm for inferring demographic history of multiple populations from allele frequency spectrum data”, to be considered for publication as a Technical Note in *GigaScience*. Our manuscript presents a novel computational tool based on the genetic algorithm for automatic unsupervised search of demographic models based on an allele frequency spectrum (AFS). In the manuscript, we give a description of the implemented algorithm and demonstrate its application in three different example systems.

To date, the reconstruction of demographic history with AFS data remains a challenging problem due to the need for many runs of local optimization procedures for models pre-specified by the user, thus limiting the overall search space of possible models. The presented software removes this bias and limitation during demographic model search and proposes a genetic algorithm for the global search of optimal demographic models given an observed AFS. We compared the efficiency of GADMA with several examples based on human data and data from two non-model species (Gillette’s checkerspot butterfly and Gaboon forest frog) and were able to find similar or better solutions in all tested examples.

Following the open data policy, both the GADMA source code and installation package are publicly available. Also, GADMA can be easily installed from the project GitHub page - https://github.com/ctlab/GADMA. The files used for demonstrating GADMA are enclosed in the paper as Supplementary Materials.

We certify that this manuscript describes the original work of the authors and has not previously been published nor submitted to any other journal at this time. A preprint version of this paper was published earlier this year on bioRxiv. All authors have approved the manuscript and agreed to this submission. We declare that none of the authors has any conflict of interest in regard to this manuscript.

We respectfully recommend the following scientists as potential reviewers for our manuscript:

1. Dr. Ryan Gutenkunst
   Department of Molecular and Cellular Biology
   The University of Arizona
   1007 East Lowell Street, LSS 325

P.O. Box 210106
Tucson, Arizona 85721
rgutenk@email.arizona.edu

2. Dr. Kirk Lohmueller
Department of Ecology and Evolutionary Biology
University of California
621 Charles E. Young Drive South
Los Angeles, California 90095
klohmueller@ucla.edu

3. Dr. Simon Gravel
Department of Human Genetics and
Genome Quebec Innovation Centre
McGill University
740 Dr. Penfield Avenue
Montreal, QC H3A 0G1
Canada
simon.gravel@mcGill.ca

4. Dr. Lounes Chikhi
Instituto Gulbenkian de Ciência
Rua da Quinta Grande, 6
P-2780-156, Oeiras
Portugal
and
Centre National de la Recherche Scientifique
Université Paul Sabatier
École Nationale de Formation Agronomique
UMR 5174 Laboratoire Évolution et Diversité Biologique
F-31062 Toulouse
France
chikhi@igc.gulbenkian.pt

5. Prof. Mike Bruford
School of Biosciences
Cardiff University
Sir Martin Evans Building, Museum Avenue,
Cardiff, CF10 3AX
United Kingdom
brufordmw@cardiff.ac.uk

Thank you for receiving our manuscript and considering it for review. We appreciate your time and look forward to your response.

On behalf of all of the co-authors,

Yours sincerely,

Ekaterina Noskova
