# Supplementary material for: GADMA: Genetic algorithm for inferring demographic history of multiple populations from allele frequency spectrum data
Source: Gigascience. 2020 Feb 29;9(3):giaa005. doi: 10.1093/gigascience/giaa005 (PMC7049072; doi:10.1093/gigascience/giaa005)
Supplement: giaa005_Supplemental_File [file giaa005_supplemental_file.pdf]

Supplementary materials for

GADMA: Genetic algorithm for inferring demographic history of multiple populations from allele frequency spectrum data

Ekaterina Noskova[1, *], Vladimir Ulyantsev[1], Klaus-Peter Koepfli[1, 2], Stephen J. O'Brien[1, 3], and Pavel Dobrynin[1, 2]

[1]Computer Technologies Laboratory, ITMO University, St. Petersburg, Russian Federation
[2]Smithsonian Conservation Biology Institute, Center for Species Survival, National Zoological Park, Washington, D.C., USA
[3]Guy Harvey Oceanographic Center, Nova Southeastern University Ft. Lauderdale, Florida, USA

[*]Corresponding author: noskova.e.ekaterina@gmail.com.

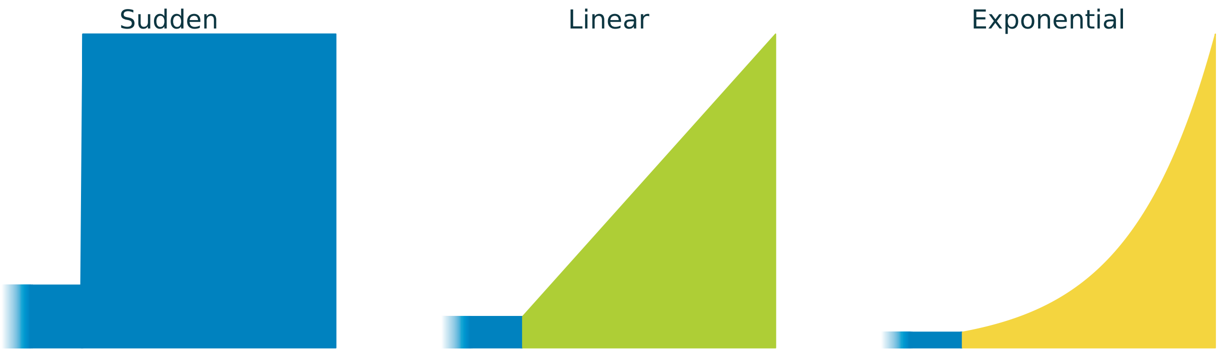

Figure S1: Diagrams of three primary demographic dynamics of population size change. Sudden population growth is very popular for applications in demographic models, as well as exponential population growth. Linear population growth is a tradeoff between sudden size change and exponential change and it is also more realistic that sudden population growth. Different colors are used to highlight different types of population growth.

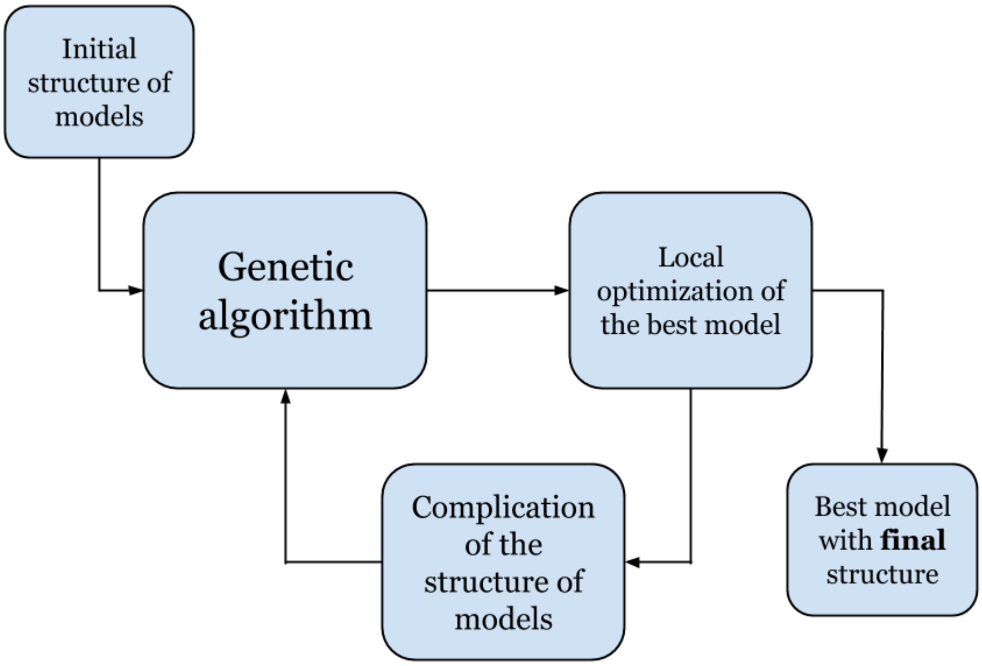

Figure S2: Diagram showing the general algorithm used in GADMA and the relationship between the genetic algorithm that performs the global optimization search followed by the local optimization search.

Table S1: Demographic models for the allele frequency spectrum using synonymous SNVs only of the Gillette's checkerspot butterfly populations (WY — Wyoming, CO — Colorado). 95% confidence intervals are indicated in brackets.

	A [1]	B1 [1]	B2 [1]	A	B2 (1)	B2 (2)	B2 (3)
Number of parameters:	3	4	5	4	5	5	5
$\log LL$	−211.63	−210.83	−210.80	−211.09	**−205.94**	−210.54	−210.76
$CLAIC$ ($eps = 10^{-4}$)	−690.53	158.07	418.11	**−1206.83**	385.23	420.30	414.57
Parameters:							
η_{WY0}	NA	NA	NA	0.790<br>[0.0 − 0.334]	0.002<br>[0.929 − 1.057]	0.999<br>[0.888 − 1.076]	(= η_{WY})
η_{WY}	0.922<br>[0.643 − 1.128]	0.884<br>[0.643 − 1.093]	0.893<br>[0.647 − 1.085]	0.960<br>[0.693 − 1.186]	0.856<br>[0.682 − 1.045]	0.742[1]<br>[0.411 − 1.071]	0.873<br>[0.645 − 1.090]
η_{CO}	0.104<br>[0.073 − 0.137]	0.119<br>[0.082 − 0.159]	0.121<br>[0.081 − 0.158]	0.064[1]<br>[0.035 − 0.092]	0.048[1]<br>[0.017 − 0.077]	0.120<br>[0.084 − 0.153]	0.121<br>[0.081 − 0.158]
m_{WY-CO}	NA	0.887<br>[0.0 − 1.601]	0.906<br>[0.0 − 1.565]	NA	2.857<br>[1.417 − 3.816]	0.952<br>[0.0 − 1.630]	0.923<br>[−0.127 − 1.589]
m_{CO-WY}	NA	NA	0.002<br>[0.0 − 0.449]	NA	0.176<br>[0.0 − 1.096]	0.0<br>[0.0 − 0.433]	0.0<br>[0.0 − 0.459]
τ_{SPLIT}	0.066<br>[0.046 − 0.084]	0.080<br>[0.050 − 0.106]	0.081<br>[0.050 − 0.106]	0.075<br>[0.052 − 0.098]	0.241<br>[0.128 − 0.306]	0.079<br>[0.053 − 0.101]	0.080<br>[0.050 − 0.106]

[1] Linear growth

Descriptions of model names in tables for Gaboon forest frog.

anc_asym_mig	Split, Ancient Asymmetrical Migration.
anc_asym_mig_size	Split, Ancient Asymmetrical Migration, Size Change.
anc_sym_mig	Split, Ancient Symmetrical Migration.
anc_sym_mig_size	Split, Ancient Symmetrical Migration, Size Change.
asym_mig	Asymmetrical Gene Flow.
no_mig	Split with No Migration.
no_mig_size	Split with No Migration, Size Change.
sec_contact_asym_mig	Split, Secondary Contact, Asymmetrical Migration.
sec_contact_asym_mig_size	Split, Secondary Contact, Asymmetrical Migration, Size Change.
sec_contact_sym_mig	Split, Secondary Contact, Symmetrical Migration.
sec_contact_sym_mig_size	Split, Secondary Contact, Symmetrical Migration, Size Change.
Structure 1,2	Split, Two Time Intervals with Asymmetrical Migrations.
sym_mig	Split, Symmetrical Gene Flow.
unidir_asym_mig_size	Split, Unidirectional Gene Flow followed by Asymmetrical Bidirectional Migrations and Size Change.
unidir_sym_mig_size	Split, Unidirectional Gene Flow followed by Symmetrical Bidirectional Migrations and Size Change.

Table S2: Demographic models for the Northern and Southern populations of Gaboon forest frog. The value of likelihood was estimated by using of $\partial a \partial i$ with $G = [50, 60, 70]$ grid size.

	N	Prev. $\log LL$	Prev. AIC	$\log LL$	AIC	ΔAIC	ω_i	θ	ν_1^a	ν_2^a	ν_1^b	ν_2^b	m_{12}^a	m_{21}^a	m_{12}^b	m_{21}^b	T_a	T_b
unidir_asym_mig_size	9	–	–	−402.00	821.99	0.00	0.89	107.0	2.515	3.339	17.139	7.057	0.033	–	0.006	0.014	5.779	2.692
Structure 1,2	11	–	–	−402.20	826.40	4.41	0.10	134.7	1.894	2.634	13.544	5.584	0.046	0.000	0.008	0.017	4.335	2.162
sec_contact_asym_mig_size	8	−445.7	907.4	**−407.21**	830.42	8.43	0.01	262.6	1.128	1.246	6.972	2.906	–	–	0.019	0.033	1.621	1.091
unidir_sym_mig_size	8	–	–	−410.26	836.52	14.53	0.00	254.3	0.987	1.304	7.045	3.043	0.055	–	0.021	m_{12}^b	1.721	1.181
sec_contact_sym_mig_size	7	−439.9	893.8	**−411.62**	837.24	15.25	0.00	279.5	1.024	1.190	6.471	2.783	–	–	0.025	m_{12}^b	1.445	1.032
anc_asym_mig_size*	8	−522.5	1061.0	**−499.19**	1014.38	192.39	0.00	90.2	3.885	5.161	24.025	8.599	0.048	0.020	–	–	8.579	2.170
anc_sym_mig_size	7	−509.8	1033.6	**−501.56**	1017.12	–	–	80.2	5.182	5.228	27.808	10.110	0.028	m_{12}^a	–	–	10.000	2.218
anc_asym_mig_size	8	−522.5	1061.0	**−500.62**	1017.24	195.25	0.00	178.2	1.873	2.543	12	4.338	0.100	0.039	–	–	3.780	1.145
anc_sym_mig_size	7	−509.8	1033.6	**−503.66**	1021.32	199.33	0.00	181.2	2.145	2.220	12	4.450	0.068	m_{12}^a	–	–	3.806	1.051
no_mig_size	5	−570.3	1150.6	**−569.89**	1149.78	327.79	0.00	570.1	0.115	0.139	3.178	1.340	–	–	–	–	0.085	0.588
sec_contact_asym_mig	6	−674.6	1361.2	**−643.79**	1299.58	477.59	0.00	209.4	6.061	2.631	ν_1^a	ν_2^a	–	–	0.036	0.063	3.441	0.218
sec_contact_sym_mig	5	−647.9	1305.8	**−647.16**	1304.32	482.33	0.00	203.4	6.197	2.745	ν_1^a	ν_2^a	–	–	0.037	m_{12}^b	3.514	0.286
sym_mig	4	−669.9	1347.8	−669.95	1347.90	525.91	0.00	156.2	8.036	3.509	ν_1^a	ν_2^a	0.012	m_{12}^a	–	–	5.306	–
asym_mig	5	−677.0	1364.0	**−669.27**	1348.54	526.55	0.00	150.9	8.321	3.609	ν_1^a	ν_2^a	0.011	0.014	–	–	5.543	–
anc_sym_mig	5	−671.7	1353.4	**−670.04**	1350.08	528.09	0.00	137.1	9.111	4.000	ν_1^a	ν_2^a	0.011	m_{12}^a	–	–	6.202	0.000
no_mig	3	−788.5	1583.0	**−788.04**	1582.08	760.09	0.00	345.1	3.911	1.607	ν_1^a	ν_2^a	–	–	–	–	1.716	–
anc_asym_mig	6	−788.5	1589.0	**−788.07**	1588.14	766.15	0.00	340.5	3.968	1.635	ν_1^a	ν_2^a	0.000	0.000	–	–	0.052	1.692

Previous values of log-likelihood and AIC scores are from [3].
N — number of parameters.
$\log LL$ — log-likelihood.
* Inferred with *moments* with higher upper bounds of parameters.

Table S3: Demographic models for the CVLN and CVLS populations of Gaboon forest frog. The value of likelihood was estimated by using of ∂a∂i with $G = [40, 50, 60]$ grid size.

	N	Prev. $\log LL$	Prev. AIC	$\log LL$	AIC	ΔAIC	ω_i	θ	ν_1^a	ν_2^a	ν_1^b	ν_2^b	m_{12}^a	m_{21}^a	m_{12}^b	m_{21}^b	T_a	T_b
unidir_asym_mig_size	9	–	–	−453.65	925.30	0.00	0.58	145.5	1.893	0.880	12.266	3.349	–	0.399	0.046	0.395	4.148	1.808
sec_contact_asym_mig_size	8	−463.3	942.6	**−455.17**	926.34	1.04	0.34	240.8	1.072	0.679	7.383	1.948	–	–	0.071	0.722	1.207	1.139
Structure 1,2	11	–	–	−453.67	929.34	4.04	0.08	134.2	2.003	0.845	13.218	3.621	0.000	0.400	0.042	0.365	5.000	1.974
unidir_sym_mig_size	8	–	–	−489.23	994.46	69.16	0.00	156.3	6.810	1.885	20.309	5.786	–	0.592	0.112	m_{12}^b	3.129	0.313
anc_asym_mig_size*	8	−519.6	1055.2	**−499.16**	1014.32	89.02	0.00	116.7	9.579	2.696	100	16.868	0.051	0.404	–	–	4.673	0.222
anc_asym_mig_size	8	−519.6	1055.2	**−500.47**	1016.94	–	–	237.6	5.822	1.368	12	12	0.091	0.769	–	–	1.660	0.090
sec_contact_asym_mig	6	−515.5	1043.0	**−505.61**	1023.22	97.92	0.00	255.2	6.209	1.738	ν_1^a	ν_2^a	0.106	0.707	0.950	0.530		
asym_mig	5	−513.5	1037.0	**−512.96**	1035.92	110.62	0.00	248.0	6.320	1.763	ν_1^a	ν_2^a	0.086	0.553	–	–	1.574	–
anc_asym_mig	6	−520.8	1053.6	**−512.98**	1037.96	112.66	0.00	247.9	6.333	1.758	ν_1^a	ν_2^a	0.085	0.557	–	–	1.574	0.000
sec_contact_sym_mig_size*	7	−537.9	1089.8	**−513.12**	1040.24	114.94	0.00	328.3	0.509	100	4.914	1.781	–	–	0.287	m_{12}^b	0.301	0.777
sec_contact_sym_mig_size	7	−537.9	1089.8	**−514.07**	1042.14	–	–	320.9	0.543	12	5.038	1.841	–	–	0.283	m_{12}^b	0.319	0.799
sec_contact_sym_mig	5	−553.8	1117.6	**−551.78**	1113.56	188.26	0.00	288.5	5.201	2.086	ν_1^a	ν_2^a	–	–	0.294	m_{12}^b	0.767	0.398
anc_sym_mig_size	7	−600.8	1215.6	**−550.34**	1114.68	189.38	0.00	254.8	4.440	2.179	12	2.810	0.323	m_{12}^a	–	–	1.447	0.112
sym_mig	4	−556.1	1120.2	**−555.37**	1118.74	193.44	0.00	268.9	5.459	2.165	ν_1^a	ν_2^a	0.228	m_{12}^a	–	–	1.342	–
anc_sym_mig_size*	7	−600.8	1215.6	**−553.00**	1120.00	–	–	72.7	10.319	7.245	54.547	9.098	0.143	m_{12}^a	–	–	8.012	0.738
anc_sym_mig	5	−558.3	1126.6	**−555.38**	1120.76	195.46	0.00	265.6	5.521	2.192	ν_1^a	ν_2^a	0.227	m_{12}^a	–	–	1.368	0.000
no_mig_size*	5	−704.6	1419.2	**−691.47**	1392.94	467.64	0.00	464.3	1.797	100	4.510	1.179	–	–	–	–	0.137	0.292
no_mig_size	5	−704.6	1419.2	**−692.26**	1394.52	469.22	0.00	465.6	1.805	12	4.530	1.192	–	–	–	–	0.137	0.288
no_mig	3	−704.4	1414.8	−704.35	1414.70	489.40	0.00	463.7	4.050	1.407	ν_1^a	ν_2^a	–	–	–	–	0.411	–

Previous values of log-likelihood and AIC scores are from [3].

N — number of parameters.

$\log LL$ — log-likelihood.

* Inferred with *moments* with higher upper bounds of parameters.

Table S4: Demographic models for the CrossRiver and CVLN populations of Gaboon forest frog. The value of likelihood was estimated by using of ∂a∂i with $G = [50, 60, 70]$ grid size.

	N	Prev. $\log LL$	Prev. AIC	$\log LL$	AIC	ΔAIC	ω_i	θ	ν_1^a	ν_2^a	ν_1^b	ν_2^b	m_{12}^a	m_{21}^a	m_{12}^b	m_{21}^b	T_a	T_b
unidir_asym_mig_size	9	–	–	−365.29	748.58	0.00	0.44	251.9	0.139	6.899	0.889	8.873	2.639	–	0.556	0.312	1.089	0.109
unidir_sym_mig_size	8	–	–	−365.31	748.62	0.04	0.43	249.1	0.177	6.876	1.164	10.172	2.117	–	0.424	m_{12}^b	1.135	0.085
anc_asym_mig_size	8	−379.8	775.6	**−368.22**	752.44	3.86	0.06	248.3	0.224	7.034	12	12	1.849	0.158	–	–	1.184	0.044
anc_asym_mig_size*	8	−379.8	775.6	**−368.23**	752.46	–	–	240.1	0.271	6.826	100	54.277	1.487	0.154	–	–	1.263	0.039
Structure 1,2	11	–	–	−365.26	752.52	3.94	0.06	250.6	0.149	7.034	0.974	8.707	2.510	0.001	0.507	0.328	1.110	0.101
sec_contact_asym_mig_size	8	−379.4	774.8	**−369.84**	755.68	7.10	0.01	259.7	0.010	6.522	0.436	7.497	–	–	1.206	0.169	0.729	0.383
sec_contact_asym_mig	6	−378.0	768.0	**−374.56**	761.12	12.54	0.00	264.7	0.369	7.076	ν_1^a	ν_2^a	–	–	1.325	0.226	0.752	0.305
asym_mig	5	−379.1	768.2	**−377.82**	765.64	17.06	0.00	256.0	0.390	7.172	ν_1^a	ν_2^a	1.014	0.168	–	–	1.145	–
anc_asym_mig	6	−379.8	771.6	**−377.83**	767.66	19.08	0.00	259.1	0.382	7.110	ν_1^a	ν_2^a	1.038	0.168	–	–	1.121	0.000
sec_contact_sym_mig_size*	7	−412.4	838.8	**−399.99**	813.98	65.40	0.00	260.3	100	5.267	0.543	6.949	–	–	0.364	m_{12}^b	0.508	0.597
sec_contact_sym_mig_size	7	−412.4	838.8	**−400.10**	814.20	–	–	261.7	12	5.539	0.531	6.936	–	–	0.372	m_{12}^b	0.525	0.565
sec_contact_sym_mig	5	−406.4	822.8	**−405.35**	820.70	72.12	0.00	305.2	0.576	6.313	ν_1^a	ν_2^a	–	–	0.739	m_{12}^b	0.645	0.122
sym_mig	4	−410.5	829.0	−410.45	828.90	80.32	0.00	266.2	0.638	6.672	ν_1^a	ν_2^a	0.355	m_{12}^a	–	–	1.071	–
anc_sym_mig	5	−411.6	833.2	**−410.44**	830.88	82.30	0.00	267.0	0.635	6.659	ν_1^a	ν_2^a	0.356	m_{12}^a	–	–	1.065	0.000
anc_sym_mig_size	7	−411.3	836.6	**−410.44**	834.88	86.30	0.00	268.2	0.621	6.593	12	12	0.365	m_{12}^a	–	–	1.061	0.002
anc_sym_mig_size*	7	**−411.3**	836.6	−443.84	901.68	–	–	67.8	4.208	18.898	1.723	35.187	1.346	m_{12}^a	–	–	7.217	0.995
no_mig_size*	5	−533.9	1077.8	**−531.74**	1073.48	324.90	0.00	411.0	100	100	0.312	4.568	–	–	–	–	0.165	0.194
no_mig_size	5	**−533.9**	1077.8	−535.43	1080.86	–	–	414.7	12	12	0.313	5.061	–	–	–	–	0.164	0.191
no_mig	3	−549.1	1104.2	−549.12	1104.24	355.66	0.00	439.3	0.432	5.904	ν_1^a	ν_2^a	–	–	–	–	0.292	–

Previous values of log-likelihood and AIC scores are from [3].

N — number of parameters.

$\log LL$ — log-likelihood.

* Inferred with *moments* with higher upper bounds of parameters.

Table S5: *CLAIC* values, calculated with different *eps* values of step size.

Model	logLL	$eps = 10^{-2}$	$eps = 10^{-3}$	$eps = 10^{-4}$	$eps = 10^{-5}$	$eps = 10^{-6}$	$eps = 10^{-7}$	$eps = 10^{-8}$
				Expected AFS was simulated with ∂a∂i				
YRI_CEU ∂a∂i	−1066.35	142701.20	33509.07	33542.39	35059.26	−71683.69	2591.71	2135.14
YRI_CEU (1)	−1066.28	28768.47	30859.18	33496.49	33702.64	9149.44	3772.52	2150.34
YRI_CEU (2)	−1065.87	169095.91	40863.03	29081.16	28389.58	71905.58	3749.39	2135.20
YRI_CEU (3)	−1065.15	NA*	50701.16	96596.45	53101.50	−20687.29	3152.25	1151.09
YRI_CEU_CHB ∂a∂i	−6316.89	45518.69	67156.30	43865.22	50036.26	40549.96	6573.50	13081.62
YRI_CEU_CHB (1)	−6314.41	−20254.09	63574.23	45182.80	40285.51	41625.18	13076.09	12616.70
YRI_CEU_CHB (2)	−6315.85	45528.82	9945.05	46161.50	55597.12	52590.14	13589.61	12561.56
YRI_CEU_CHB (3)	−6288.90	42590.00	42799.62	42761.68	22702.74	104324.24	19052.70	−7500.99
				Expected AFS was simulated with *moments*				
YRI_CEU ∂a∂i	−1066.82	46214.05	46211.02	46211.70	46261.53	47383.61	9454.61	680.55
YRI_CEU (1)	−1066.65	46203.96	46200.95	46200.83	46150.78	47724.78	6605.69	2057.33
YRI_CEU (2)	−1067.15	53110.78	53107.65	53107.60	53037.09	52954.46	−19076.40	454.91
YRI_CEU (3)	−1066.35	50564.91	52729.74	52737.56	52662.46	56683.27	19571.01	5117.64
YRI_CEU_CHB ∂a∂i	−6316.58	172826.53	172863.16	172877.27	170918.12	749938.13	17485.76	11341.11
YRI_CEU_CHB (1)	−6315.60	35299.20	35480.68	35482.63	35496.75	39297.26	25061.12	15402.95
YRI_CEU_CHB (2)	−6316.50	210828.67	210895.13	210853.81	203959.88	395581.64	24185.76	11617.53
YRI_CEU_CHB (3)	−6288.90	42590.00	42799.62	42761.68	22531.36	−24921.12	25659.62	10414.80
syn_A McCoy et al.	−211.63	−693.91	−690.69	−690.53	−665.99	−838.64	984.62	437.21
syn_A (model 24)	−211.09	−1234.67	−1207.16	−1206.83	−1161.88	−1229.47	844.73	432.36
syn_A (model 28)	−211.43	−317.04	−314.99	−314.97	−303.53	−333.70	435.30	434.68
syn_A (model 3)	−211.60	−556.38	−553.72	−553.75	−535.46	−620.42	673.23	513.07
syn_A (model 5)	−211.82	3208.40	3868.78	3877.32	4288.25	2837.31	552.32	426.85
syn_B1 McCoy et al.	−210.85	157.64	158.07	158.07	159.76	158.80	778.18	479.26
syn_B2 McCoy et al.	−210.80	417.99	416.84	418.11	418.02	406.78	−145.01	433.80
syn_B (model 37)	−205.92	385.05	385.23	385.23	418.82	366.01	−4541.26	424.53
syn_B (model 49)	−210.54	425.68	420.82	420.30	420.38	564.95	393.81	416.98
syn_B (model 35)	−210.76	420.40	415.14	414.57	414.23	360.69	279.37	438.99
syn_B (model 23)	−212.15	443.83	426.35	426.03	426.75	419.33	372.42	416.72
all_A McCoy et al.	−283.60	1621.03	1620.86	1620.85	1619.13	1610.11	496.93	584.66
all_A (model 20)	−277.82	1618.87	1619.01	1619.03	1620.04	1640.58	1133.75	593.70
all_A (model 23)	−282.88	1239.62	1239.69	1239.70	1240.31	1259.52	821.94	579.03
all_A (model 5)	−283.53	1638.54	1638.38	1638.37	1637.97	1625.49	−65.81	456.35
all_A (model 12)	−284.81	1731.63	1731.67	1731.68	1733.36	1772.97	871.78	725.01
all_B (model 2)	−267.49	−354.57	−353.70	−354.00	−333.05	40.35	−63.50	NA
all_B (model 26)	−282.67	−184.54	−183.60	−185.48	−187.93	−178.71	823.15	620.20
all_B (model 19)	−278.45	55.25	55.63	51.85	59.97	34.25	895.73	350.68

$\log LL$ — log-likelihood.

* — numerical issue during the evaluation: singular estimation of Hessian matrix.

1 Simulations

We tested GADMA using simulated data. To simulate the allele frequency spectrum, we used *moments*, and Powell's method was used for local optimization. We tested one demographic model for one, two and three populations (see below for details). All simulated spectra were folded, with a size 20 chromosomes per population.

Three different pipelines were compared: 1) a local search from several different starting points, 2) the $\partial a \partial i$ *pipeline* with several rounds of local optimization, and 3) GADMA. All pipelines took into account the known demographic model and were launched multiple times (50 times for one and two populations and 10 times for three populations). For all inferences, we recorded the mean time for launch, mean number of iterations, mean and standard deviation of the log-likelihood and the estimated best log-likelihood. In addition, two extra pipelines of GADMA without knowledge of the model were tested: with and without fixed change in sudden population size. Therefore, a total of five different optimizations were compared and the results are provided in Tables S6-S8.

We note that the values such as mean run time ("Mean time") are presented only for informational purposes and that the mean and standard deviation of the log-likelihood should be compared only for the first three base optimizations. Iteration = one evaluation of fitness function, thus evaluation of likelihood for one demographic model. Time per iteration will vary as it differs for different values for the parameters of the demographic model. The number of initial parameters for the local optimization pipeline and the number of replicates in the $\partial a \partial i$ *pipeline* were selected so that the mean number of fitness function evaluations were similar to that using GADMA.

1.1 One Population

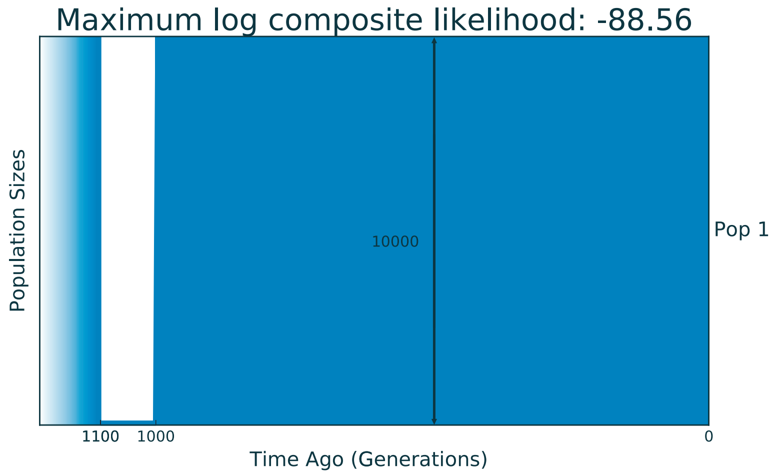

Figure S3: Demographic model for one population that was used in order to test GADMA on simulated data.

Table S6: Results of 50 runs of optimizations on the simulated data for one population.

	True values	Local search	$\partial a \partial i$ *pipeline*	GADMA custom model	GADMA sudden dynamic	GADMA full model (1)	GADMA full model (2)
Mean time:	–	06^m40^s	05^m22^s	07^m25^s	06^m17^s	12^m54^s	
Mean iter:	–	7860	7951	7515	16977	13688	
Mean logLL:	–	−293.4815	−338.1879	−132.9586	−139.682	−97.9999	
Std logLL:	–	136.2765	116.9429	99.9209	96.2198	43.5008	
Best logLL:	−88.5603	−88.6202	−88.5780	−88.5832	−88.5647	**−88.5616**	−88.5711
N_A	10000	9999	10028	10038	10010	10034	10018
N_B	100	219	203	201	146	178^e	180
N_F	10000	10180	10159	10094	9972	10050	10011
T_B	100	112	103	205	147	791	182
T	1000	971	975	978	994	985	986

For one population, we chose to simulate a demographic history that included a bottleneck: the effective size of the ancestral population was 10,000 individuals, followed by a bottleneck 1,100 generations ago that reduced the population size to 100 individuals for 100 generations, following which the population size expanded to 10,000 individuals again (Figure S3).

Each local optimization was launched from 40 initial points. The inference with the $\partial a \partial i$ *pipeline* had five rounds of 10, 10, 10, 10, and 20 replicates.

Although the $\partial a \partial i$ *pipeline* showed the best maximum-likelihood model among the three base optimizations, GADMA with a customized demographic model produced the best mean and standard deviation of the log-likelihood for the final models and is better on average. As for the simulations with GADMA that included no prior knowledge about the demographic model, these showed a better mean and standard deviation of the log-likelihood and were closer to the optimum value for the best model. However, the number of iterations and run times were double the values compared to the simulations that included optimizations with prior knowledge of the demographic model. Runs without a fixation of population size change

dynamics provided the best mean and standard deviation of the log-likelihood as well as two alternative models with similar likelihood values. One of these included a model with an exponential decrease in population size following sudden growth and the other included the same population bottleneck model as described above. Indeed, similar results were presented in [2], as the example of two demographic models that have the same expected AFS.

1.2 Two Populations

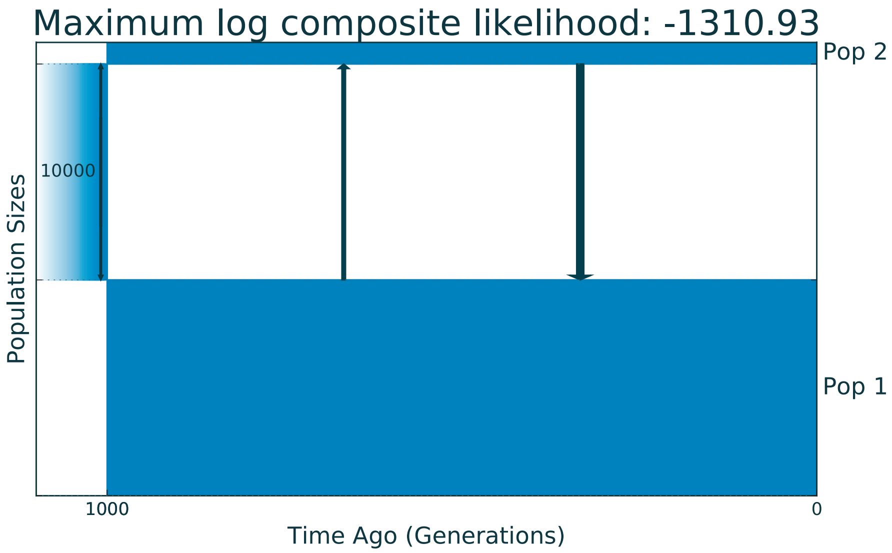

Figure S4: Demographic model for two populations that was used in order to test GADMA on simulated data.

For two populations, we choose the following demographic history: the effective size of the ancestral population was 10,000, which then divided into two populations 1,000 generations ago, with a size of 10,000 individuals (Pop 1) and 1,000 individuals (Pop 2) (Figure S4). Migration between the populations was equal to: 2.5×10^{-4} individuals from Pop 2 to Pop 1 per generation and 1.25×10^{-4} individuals from Pop 1 to Pop 2 per generation. The number of initial points for local optimization was equal to 6 and the *∂a∂i pipeline* was launched for four rounds with 10, 10, 10 and 20 replicates.

The pipeline using local optimization from different starting points and GADMA with a customized demographic model found models with likelihood scores very similar to those of the optimum demographic model. However, the *∂a∂i pipeline* was better than GADMA on average. GADMA without prior demographic model knowledge returned models with parameters and likelihood scores similar to the optimum. The result for the model for which no prior demographic history was known and population size changes were not fixed showed the sudden changes in population sizes as they were originally used in the simulation of the data.

Table S7: Results of 50 runs of optimizations on the simulated data for two populations.

	True values	Local search	*∂a∂i pipeline*	GADMA custom model	GADMA sudden changes	GADMA full model
Mean time:	–	$0^h30^m39^s$	$0^h55^m12^s$	$0^h30^m34^s$	$0^h31^m15^s$	$3^h43^m24^s$
Mean number of iter.:	–	7626	8928	7437	9947	17136
Mean likelihood:	–	−1310.948	−1311.016	−1311.269	−1311.052	−1321.195
Std likelihood:	–	0.027	0.241	0.451	0.180	13.861
Best likelihood:	−1310.931	**1310.931**	−1310.932	**−1310.931**	−1310.962	−1310.983
N_A	10000	10000	10001	10000	10001	10001
N_1	10000	10000	9992	10003	9986	10007
N_2	1000	1000	1000	1000	1004	997
$M_{12}(\times 10^{-4})$	2.50	2.50	2.50	2.50	2.51	2.50
$M_{21}(\times 10^{-4})$	1.25	1.25	1.25	1.25	1.25	1.26
T	1000	1000	1000	1000	1004	996

1.3 Three Populations

For three populations, we choose the following demographic history. The effective size of the ancestral population was 10,000, followed by the division into two subpopulations 1100 generations ago of size 15,000 (Pop 1) and 5,000 (Pop 2 + Pop 3). Then the second subpopulation split into two additional subpopulations of size 5000 (Pop 2) and 10,000 (Pop 3) (Figure S5). Migration occurred only 1,000 generations ago right after the second population split and are symmetrical: 0.25×10^{-4} individuals per generation between Pop 1 and Pop 2, 0.5×10^{-4} between Pop 1 and Pop 3 and 1.5×10^{-4} between Pop 2 and Pop 3.

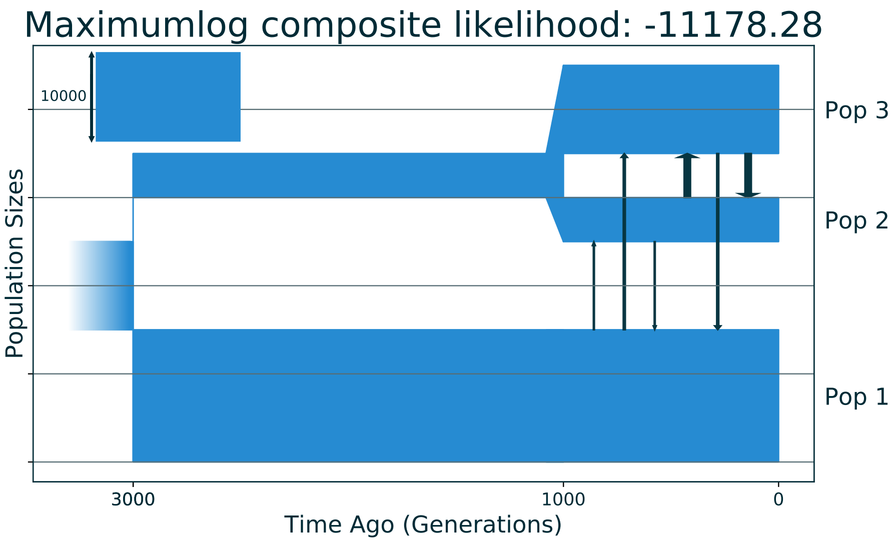

Figure S5: Demographic model for three populations that was used in order to test GADMA on simulated data.

Table S8: Results of 10 runs of optimizations on the simulated data for three populations.

	True values	Local search	*∂a∂i pipeline*	GADMA custom model	GADMA sudden changes	GADMA full model
Mean time:	–	$28^h28^m13^s$	$45^h09^m30^s$	$27^h16^m08^s$	$44^h21^m35^s$	$45^h05^m00^s$
Mean number of iter.:	–	22475	19452	21651	53623.6	71534
Mean likelihood:	–	−11178.62	−11179.82	−11178.45	−11228.47*	−11250.09
Std likelihood:	–	0.40	0.72	0.15	91.36*	123.72
Best likelihood:	−11178.28	−11178.31	−11178.59	−11178.29	−11179.55	−11180.28
N_A	10000	10003	9988	9996	10033	9997
N_1^a	$= N_1^b$ (15000)	NA	NA	NA	14715	14612
N_{2+3}^a	$= N_2^b$ (5000)	NA	NA	NA	4806	4801
$M_{1(2+3)}^a (\times 10^{-4})$	NA (0)	NA	NA	NA	0	0.14
$M_{(2+3)1}^a (\times 10^{-4})$	NA (0)	NA	NA	NA	0	0.27
N_1^b	15000	15018	15029	15004	15250	15567^e
N_2^b	5000	4992	5011	5008	4956	5032
N_3^b	10000	9984	9850	10026	9848	10050
$M_1^b 2 (\times 10^{-4})$	0.25	0.25	0.24	0.25	0.24	0.23
$M_1^b 3 (\times 10^{-4})$	0.50	0.50	0.50	0.50	0.46	0.47
$M_2^b 1 (\times 10^{-4})$	$= M_1^b 2$ (0.25)	NA	NA	NA	0.24	0.21
$M_2^b 3 (\times 10^{-4})$	1.50	1.51	1.55	1.50	1.78	1.54
$M_3^b 1 (\times 10^{-4})$	$= M_1^b 3$ (0.50)	NA	NA	NA	0.49	0.44
$M_3^b 2 (\times 10^{-4})$	$= M_2^b 3$ (1.50)	NA	NA	NA	1.70	1.60
T_a	2000	1998	1996	1999	1906	2055
T_b	1000	1000	1009	1000	1058	1020

* 9/10 runs. The worst run had the best value of log-likelihood equal to −13801.48.

Each local optimization was run from 12 initial points and the *∂a∂i pipeline* had 6 rounds with 10, 10, 10, 10, 10, and 20 replicates.

In case of three populations, GADMA with the customized demographic model showed the best maximum, mean and variation of likelihood scores (Table S8). For the extra two optimizations without prior demographic model knowledge, all migrations were very similar to symmetrical, although they were inferred as asymmetrical. All additional population sizes that were inferred are similar to the their true values. Thus we can state that GADMA could restore the demographic model without knowledge of its structure. One exception exists however: migration after the first split is not equal to zero in the case of the GADMA optimization without fixation of the population size change. We argue that this could be because not enough runs were performed. Also, the population size change parameter varies more between different resulting models than other parameters.

References

[1] Rajiv C McCoy, Nandita R Garud, Joanna L Kelley, Carol L Boggs, and Dmitri A Petrov. Genomic inference accurately predicts the timing and severity of a recent bottleneck in a nonmodel insect population. *Molecular ecology*, 23(1):136–150, 2014.

[2] Simon Myers, Charles Fefferman, and Nick Patterson. Can one learn history from the allelic spectrum? *Theoretical Population Biology*, 73(3):342–348, 2008.

[3] Daniel M Portik, Adam D Leaché, Danielle Rivera, Michael F Barej, Marius Burger, Mareike Hirschfeld, Mark-Oliver Rödel, David C Blackburn, and Matthew K Fujita. Evaluating mechanisms of diversification in a guineo-congolian tropical forest frog using demographic model selection. *Molecular ecology*, 26(19):5245–5263, 2017.
