# Supplementary material for: GADMA: Genetic algorithm for inferring demographic history of multiple populations from allele frequency spectrum data
Source: Gigascience. 2020 Feb 29;9(3):giaa005. doi: 10.1093/gigascience/giaa005 (PMC7049072; doi:10.1093/gigascience/giaa005)
Supplement: giaa005_Response_to_Reviewer_Comments_Original_Submission [file giaa005_response_to_reviewer_comments_original_submission.pdf]

Author's Response To Reviewer Comments

Close

Dear Editor and Reviewers,

We are very grateful for the reviewers' comments and their helpful suggestions for improving our manuscript. Based on these suggestions, we have conducted additional experiments using simulated data and included additional text or revised earlier text as necessary. We believe these changes have improved the quality and value of our work thanks to reviews. A point-by-point response to the comments can be found below:

Reviewer #1:

"To avoid needless obfuscation, I am Ryan Gutenkunst, developer of dadi, and I previously reviewed this manuscript for another journal.

Overall, the authors' approach has value. The optimization and model selection step of using dadi and moments is certainly a major obstacle for many users. Applying a genetic algorithm is a sensible improvement to the existing local approaches, and I like the adaptive aspect of the genetic algorithm. In my previous review, I raised serious methodological concerns that the authors' had not addressed the issue of linkage in the inference. The authors did make changes to address my concerns, but critical details are lacking. Given those methodological concerns, I have not yet dug deeply into the applications sections."

We thank Professor Gutenkunst for his comments. We believe we have addressed the methodological concerns with respect to the issue of linkage (see below).

"1) The authors have now adopted a composite likelihood version of AIC, but implementation of such is non-trivial, and the authors omit crucial details. In particular, calculating the CLAIC involves an expectation over dL/dtheta. This is non-trivial to calculate, because it is zero at the maximum CL point. How are the authors calculating it? (In our own work on composite likelihood statistics, this required bootstrapping the data, which, as I note below, is complex.)"

In order to calculate the CLAIC, we have applied and used the approach presented by Coffman et al., 2016, on which Dr. Gutenkunst is a co-author. These authors propose a method that uses bootstrapped data to estimate Hessian and Godambe matrices, which are used in the CLAIC calculation. In general, such calculation of the CLAIC is very tricky: it depends on the value of the gradient step size, which is denoted as epsilon. We have performed several experiments using empirical data and discuss these in our revised manuscript (pages 4 and 9).

"2) The authors claim that the 95% confidence intervals in Tables 2,4, and 5 are calculated via bootstrap. But they provide no details, and they appear to be calculating them incorrectly.
I note that their uncertainties in Table 4 remain much smaller than in Gutenkunst (2009). This suggests that the authors are ignoring the effects of linkage on the inference. Note that one cannot perform a proper bootstrap given only the AFS derived from the data. To account for linkage (and in the case of Gutenkunst (2009), projection) one must go back to the original genomic data to generate the pseudo-replicates, by bootstrapping over regions of the genome.
Going back to the original genome data may be out-of-scope for the authors' approach. That's fine, the authors should just emphasize that their approach is good for finding the maximum CL parameters and guiding model selection, but further work is needed for estimating confidence intervals."

Thank you for this comment. Unfortunately, the bootstrapping was performed the wrong way. In order to get valid confidence intervals for linked data, bootstrapping should be done over different regions of the genome. In the first version of our manuscript, we did not consider the linkage. Although Professor Gutenkunst suggested that going back to the original data is out of the scope for the present work, we

have nonetheless tried to do it anyway. We considered this issue in the analyses of modern humans (in the cases of two populations and three populations) and the Gillette's checkerspot butterfly.

For two human populations (YRI, CEU), the bootstrapped data was found in the example folder of dadi. We are very thankful to Professor Gutenkunst for him sharing the bootstrapped data set for for three human populations (YRI, CEU, CHB). As for the checkerspot butterflies, unfortunately there is no information about the genome regions. However, McCoy et al. 2013 provided information about the assembly of the transcriptomes used in their paper. We used this data and performed bootstrapping across the contigs of the transcriptome assembly for which we assumed that sites are unlinked across different contigs. Although this is not the most ideal situation to mitigate the effects of linkage, it is the best we can do right now.

Reviewer #2:

"Comments on ms GIGA-D-19-00143 entitled "GADMA: Genetic algorithm for inferring demographic history of multiple populations from allele frequency spectrum data" by Noskova and colleagues.
In the present manuscript the authors present a new software/program that uses a genetic algorithm approach to explore a family of predefined models and identify the best model to explain genomic data obtained from several (up to three) populations and summarised with the allele frequency spectrum (AFS). The authors take a set of three published data sets and show that they manage to identify models with higher likelihoods than those identified by the original authors. In general I thought that the ms was very well written and clear. I also thought that it was well-balanced and potentially very useful to many users/readers. As the authors note the choice of a demographic model is sometimes arbitrary and their algorithm allows population geneticists to explore many models that would have been difficult to test by hand. I thus fully support the publication of this manuscript."

We would like to express our gratitude to the Reviewer for his support and comments.

"As a general comment I note that the ms may be a bit long. By trying to analyse three data sets the authors make the ms particularly lengthy. I let the editor and authors decide on this issue, but I would a priori think that it should be published pretty much as is in terms of length, as it allows users to fully understand what was done. Altogether GADMA will be very useful to many users."

As the Reviewer noted, the manuscript is indeed quite long. While we attempted to make it shorter several times, we found that this always affected the clarity of the concepts we are presenting. Therefore, we feel that the manuscript is appropriate in length in that it provides readers with the necessary details to fully understand the methods we have implemented in the GADMA tool.

"I also believe that the authors could be clearer regarding the fact that they are using existing methods (dadi and moments) as an engine for demographic inference. GADMA allows the users to explore many scenarios automatically. The authors state it explicitly but in some sections it seems as if they were proposing a completely new method. I am sure that this is not what they meant, but it might be misunderstood. These comments are not meant to minimize their work but rather to clarify what their work allows."

We are very sorry for this misleading description. We have revised some sentences accordingly to improve clarity. We thank the Reviewer for these important comments.

"Below I provide additional comments which will hopefully be useful to improve some parts of the ms.
Introduction
The Introduction is generally very clear and agreeable to read. I only have minor comments:
* The authors write that "fastsimcoal2 can handle any number of populations". It may be true that it can simulate a (perhaps arbitrarily) large number of populations, but I do not think that this is true for demographic inference. It can deal with more pops than dadi, but I would think that above five populations it will be unreliable. Can the authors check and confirm?"

In the original paper by Excoffier et al. 2013 reporting on the fastsimcoal2 program, the authors conducted simulations to infer the demographic histories for 10 different populations. The authors also

state that fastsimcoal2 “can successfully handle models including more than three populations.” Therefore, we have revised the text in the Introduction to reflect the statements in Excoeffier et al. 2013.

“* at some points the authors compare dadi, moments and momi2 but then momi2 seems to disappear from the picture.”

Momi2 is a new and very interesting software for inferring the demographic histories from models involving 8 or more populations using AFS data. Like fastsimcoal2, we did not incorporate momi2 into GADMA. This was mainly because the interface is different from dadi and momi2 is quite new. However, we are considering adding momi2 and fastsimcoal2 to GADMA in the future so as to make the tool even more versatile. For now, we clarify the point about not using momi2, we have added additional sentences in the manuscript.

“Materials and Methods
This section is also clear but rather technical for me. I mainly read it superficially. I took the liberty to provide the ms to a PhD student with a math background and I am adding here some the comments made.
- The authors seem to have designed a genetic algorithm from first principles (they only cite a single 1975 paper on the subject) specifically for the task of exploring the possible demographic models of two or three populations, which can split, and grow in one of three possible ways (instantaneous, linear and exponential). How difficult would it have been to have a wider scope?“

Conceivably, it would not be very difficult to scale up to more populations with a wider diversity of dynamics through time. However, since we implemented GADMA starting with dadi and moments, we were interested in showing how inferred models could be obtained with simple demographic scenarios using the genetic algorithm. In the future, we plan to incorporate other inference models such as fastsimcoal2 and momi2 (as discussed above) as well as expand the number of demographic scenarios in order to infer more realistic models. However, we note that AFS data only provides a rough estimation of population history in general.

“Why didn’t the authors use one of the many standard general purpose GA? there are several of them which are readily usable from Python, with robust implementations that build and expand upon the original ideas behind the GA methods. It would have been interesting to see a comparison between the method proposed by the authors and a standard, general purpose GA, like Differential Evolution (R. Storn and K. Price, 1997) for example.
Does it make sense? Can the authors comment on these questions?”

It is a very good comment. However, the main point is that we have chosen the specific version of the genetic algorithm “from first principles” and applied it to the problem of historical demographic inference. Unfortunately, the usual methods (not their extended versions), like differential evolution are not supposed to optimize discrete variables, which is the case for the dynamics of population size change. However, we have compared several methods like differential evolution and 1+(lambda, lambda) GAs for some demographic histories with continuous variables, but they were not significantly better. Moreover, we assumed the existence of more powerful optimization method but we are not focused on that in this work. We have added additional sentences at the end of the discussion.

“An issue which was also missing for both of us is the following: the authors have applied their method to three data sets but they have not really carried out any validation on simulated data sets. They mention that in the discussion as being beyond the scope of the ms. I understand that analysing the data sets was a lot of work but it would be important to do this validation at some stage.”

We have now performed primary simulation tests for three demographic models. We included these results in the supplementary materials and show the advantage of global optimizations using GADMA compared to local optimizations. See supplementary tables S6-S8.

“Results
This is a very long section due to the fact that the authors have analysed three data sets and to the fact that the human data set has been analysed very carefully. While some authors may find this too long one could also argue that this is a proof of the thoroughness of the authors’ work. Like the rest of the ms it is very well written and clear. So, I will not ask for a shortening of that section. My comments are limited again to relatively minor issues.

- the unit of the mutation is not given (page 6, right hand column)"

Fixed. The unit of mutation is given as per site per generation.

"- I did not understand why the "logarithms were used to calculate confidence intervals" What does that change? Shouldn't confidence intervals be independent of the scaling representation? Probably just me."

What we meant is that confidence intervals were calculated under the assumption that values are log-normally distributed. Such an assumption, for example, was considered in Gutenkunst et al., 2009. In order to estimate the confidence intervals, one should apply a logarithm to the values, calculate the bounds of the interval under the assumption that it is normally distributed and finally apply the reverse transformation (exponenta) to the bounds. This will produce wider confidence intervals, which will be biased to the right.

"- the result that continental populations separated 400,000 years is rather interesting but it is discarded by the authors, which then use a constraint that the oldest split must be less than 150,000 years ago. I realise that this is not the objective of the paper and I fully understand that the authors had to constrain their work to current beliefs and interpretations of human evolution (at least among some researchers), but this is a rather noticeable result that needs some discussion or some reference to existing literature (see my comments below). I realise and stress that the authors are proposing a software. This result is thus an issue about human evolution and not about GADMA, so the authors have no duty except to mention and discuss this point a little. See below."

See below.

"- the authors mention likelihood value of -6323.99 "which slightly differs from the optimal log-likelihood -6316.89". don't they differ by 7 orders of magnitude? Not exactly a slight difference, right?"

The Reviewer is correct, it is 7 orders of magnitude. We revised the sentence accordingly as follows: "...which slightly differs from the optimal log-likelihood –6316.89, which is much less than that from the comparison of two populations. "

- typo Gillitte → Gilette

Thank you. Fixed.

"Discussion
Generally well written, this is the section that may need some work to provide a slightly more balanced review of the situation.
- The beginning of the Discussion may be a bit misleading (as I noted above) when they start by writing that GADMA "allows the automatic inference of demographic history of up to three populations ...". As I understand it the authors have developed a general program that runs dadi or moments and then changes some aspects of the model without changing fundamentally the original structure. It might be better to start by identifying the limitations of dadi (one model must be assumed, and changing it would require a lot of work). Then, this would allow the authors to make it clear that GADMA solves that problem."

See above.

"- the authors could explain why they cannot do the same with Fastsimcoal2 which allows to do inference with a (slightly) larger number of populations. This could lead to a short paragraph on what could be expected of genetic algorithms for parameter space exploration."

As we noted above, there is no inherent problem with incorporating fastsimcoal2 into GADMA to infer demographic models for more populations, which we plan to do in the future. However, there are some problems with increasing the number of populations. Mainly, it is not obvious how to infer the tree of the divergence for more than three populations. It could be done in several ways. For example, by adjusting the crossover operation in the Genetic Algorithm, which requires further developments. We plan to explore this and other methods further to update GADMA in the future.

"- the discussion of the inferred 400,000 years is tricky. I do not think that it is the authors'

responsibility to discuss it in an article on a software. At the same time, this is a result popping out in their analyses and they may want to write something about it. The authors write that Gutenkunst et al "noted the high quality of this data set." I do not know if they were ironical but that is (very) fine with me (both ways). Now I would like to note that the authors could link this result with recent studies that suggest that patterns of genomic diversity could be explained by models in which humans lived in structured populations (metapopulations) in Africa. Papers of Mazet et al (2016), Chikhi et al (2018) or Rodriguez et al (2018) all in Heredity all address these issues with increasing detail and complexity by focusing on the IICR (inverse instantaneous coalescence rate). The review/comments by Scerri et al (2018) in TREE also discuss this point: what kind of structured model should we use to better represent human evolution. It is important to stress that none of the IICR papers use "population tree models". So, my point is the following: it could be that the authors' very old estimate for "continental split times" is simply due to the fact that tree models are not the appropriate way to analyse human data. These are old ideas and the authors may want to go back to older literature. But again, these are just ideas that the authors may decide to use, or not. There is no absolute need to cite the papers I mentioned, but I am convinced that they hold some potential answers to the questions raised by this very ancient divergence time. I may be biased of course. "

We agree with the Reviewer that detailed discussion of the divergence between African and other human populations is beyond the scope of our manuscript. However, the Reviewer raises an important point regarding the estimation of population divergence times under a bifurcating tree model when such a model may be inappropriate or not even realistic given the high likelihood of admixture, introgression and genetic structure among populations, which violate the fundamental assumptions of such a model. We have added text related to this point in the manuscript.

"The Discussion could also have addressed the general methodological difficulty of performing demographic inference using the AFS. More specifically (as noted by my PhD student), the conclusion of Rosen et. al. 2017 (Geometry of the sample frequency spectrum and the perils of demographic inference) is that the AFS may be much more limited than people think (see also Chikhi et al., 2018 on the Yang et al (2012) article). The authors do mention in the final discussion that "Because of such behavior, the structure of the demographic model should not be very complex. We suggest to use structures no more than (2,1) and (2,1,1)", but then after a few paragraphs they follow with "Another direction in the further development of our work is increasing the number of considered populations". The authors may want to clarify how complex a model should be and they could discuss this in relation to the Mazet and colleagues papers mentioned above, and the IICR as a complementary summary of genomic information as noted by Mazet and colleagues."

The limitation on how informative AFS could be is very restrictive. As the Reviewer noticed a lot of papers are devoted to this problem. Although, we propose the increasing of population number, we assume the simple structures of demographic models as in the case of two and three populations. However in order to get more reliable results we suggest to incorporate it together with some additional summary about data, for example, haplotype information or IICR as the Reviewer proposed. We have added sentences about it in the discussion and cited Rosen et al., 2018 and Terhorst and Song, 2015.

"In a few words, and to conclude, I feel that the manuscript and the GADMA software will be useful and used by many biologists who are sometimes lost in deciding which model they should use with dadi. GADMA will help reduce some arbitrariness in the exploration of models. I do not need to see a revised version of the ms but will be happy to if the editor feels that it is necessary. Congratulations to the authors for an interesting article, a useful software and a thorough analysis of demographic inference."

We are grateful to the Reviewer for their supportive words and enthusiasm for the GADMA software!

Close
