# Supplementary material for: GADMA: Genetic algorithm for inferring demographic history of multiple populations from allele frequency spectrum data
Source: Gigascience. 2020 Feb 29;9(3):giaa005. doi: 10.1093/gigascience/giaa005 (PMC7049072; doi:10.1093/gigascience/giaa005)
Supplement: giaa005_Response_to_Reviewer_Comments_Revision_1 [file giaa005_response_to_reviewer_comments_revision_1.pdf]

# Author's Response To Reviewer Comments

Close

Dear Editor and Reviewer,

We are very grateful for your comments and suggestions. In the revised version of the manuscript we included additional text and also omitted some figures in order to make it more clear and concise. Changes are highlighted. A point-by-point response to the comments can be found below:

"Reviewer #1: I am relieved that the authors have taken my concerns to heart and fixed the major issues related to linkage in the data. With that major methodological flaw corrected, I have further concerns regarding the manuscript, particularly the discussion of composite likelihood.

1) Page 3, Discussion of inputs to the method: If the authors are carrying out the CLAIC test correctly, then the AFS A and the constants C are not sufficient to fully specify the problem. The collection of bootstrapped data sets is also a necessary input. The authors should make this explicit."

In the formulation of the problem as it is stated in the manuscript, there is no need to include bootstrap data as input since the problem is formulated in terms of an optimization problem with the likelihood function (not CLAIC) as fitness. CLAIC is just an addition to the existing solution of this problem. If we were optimizing by CLAIC, then obviously bootstrapped data should be mentioned as a necessary input.

"2) More broadly, the authors should carefully discuss the need for block-bootstrapped data as input to the CLAIC calculation. This is a critical point that the authors themselves missed in the first two submissions of the manuscript. They should be very explicit about it in the present manuscript, so as not to mislead users, like they themselves were misled."

We agree with this comment and have added a wider discussion to make the importance of block-bootstrapped data more clear (line 304 and line 1185):

"3) In addition to discussing proper block bootstrapping in the manuscript, it must be described in the GADMA manual. As far as I can tell, the only mention of bootstraps in the documentation is in the example_params file, where many users may be confused or overlook it."

We updated the GADMA manual (pages 9, 23 and 24) according to these recommendations.

"4) For the human analysis, the authors should be explicit about how the bootstrapping was done for these data. I recognize that it is parroting Gutenkunst (2009), but explicit is better than implicit here."

We have added the following description (line 589):
For the human population data, we used the block bootstrapped data set from Gutenkunst et al. 2009, where it was done over 219 sequenced loci under the assumption that the loci are well separated and can be treated as independent.

"5) Page 4: I am confused by the number of parameters in the specification. For example, in a two population interval I count 6 parameters (2 migration rates, 2 final population sizes, and 2 growth modes). What is the 7th parameter? I ask the authors to be much more explicit about how they are parameterizing the models, perhaps using Figure 5 as an example."

We apologize for the confusion. In the first version of GADMA, there was one additional parameter - size of ancestral population, and the inference was not multinomial. We have changed the formula so as that to conform better to realistic conditions (line 250). We have not added the parametrization of the model to Figure 5 (Figure 3 in new version), but we extended the description of the figure in the figure caption.

"6) Page 5, Mutation of the demographic model: The described process only applies to continuous parameters. Is the mode of population size change (instant, growth, exponential) also allowed to

mutate? If so, how? (I raised this question in my initial review, which the authors ignored.)”

Yes, population size change dynamics are the parameters for mutation that can be mutated and we have clarified this in the corresponding sentences (line 367):
Among the parameters of the demographic model that can be mutated during estimation is the mode of population size change (sudden, growth, and exponential). If this parameter is chosen to be mutated, then the value (mode) will change to one of the other two population size change dynamics with equal probability.

“7) Page 6, Increasing model complexity: Is this feature used in any of the examples the authors present? From the Results section, it appears the authors have pre-specified the model structure for all analyses, rather than allowing the algorithm to do it. If this is the case, I suggest the authors remove this section. If they have indeed used this feature, they should be more clear when describing their results.”

The increase of model structure was used in our analysis of human data (two populations), where the initial structure of the model was 1,1 and the final model structure was 2,1. We have clarified this in the revised manuscript (line 465).

“8) To shorten the manuscript, I suggest that many extraneous figures be removed. Figures 1,2,4,6,7 add very little to the exposition.”

Figures 1 and 2 were incorporated into a single figure. Figures 4 and 6 were moved to the Supplementary materials. We believe it is important to keep Figure 7 (Figure 4 in new version) in the main text so that the unrelated reader could easily understand the concept of the genetic algorithm.

Close
