# Supplementary material for: GADMA: Genetic algorithm for inferring demographic history of multiple populations from allele frequency spectrum data
Source: Gigascience. 2020 Feb 29;9(3):giaa005. doi: 10.1093/gigascience/giaa005 (PMC7049072; doi:10.1093/gigascience/giaa005)
Supplement: giaa005_Response_to_Reviewer_Comments_Revision_2 [file giaa005_response_to_reviewer_comments_revision_2.pdf]

Author's Response To Reviewer Comments

Close

Dear Editor,

We are grateful for your comments and believe that our edits will improve the clarity of the manuscript. A point-by-point response can be found below:

"1) Supporting data / archival snapshot of code in GigaDB:

Our data curators will contact you shortly to prepare any supporting data (and/or an archival copy of the code) for release via our repository GigaDB. Once this is sorted, please include a citation to your upcoming GigaDB dataset (including the DOI link) to your reference list, and cite this dataset in the "data availability" section and elsewhere in the manuscript, where appropriate.

Please follow this example for the reference:

[xx] Author1 N, Author2 N, AuthorX N. Supporting data for "Title of your manuscript". GigaScience Database. 2019. http://dx.doi.orgxxxxxxxxxxxx

You will get the exact citation, including the DOI link, from our curators."

We have published the supplementary materials as GigaDB dataset and cited it in the manuscript.

"2) Citing your bitbucket repository.
I see you link to a bitbucket repository in the supplemental files. Please also cite this in in the "availability" section of the paper and include a reference to the bibliography, including a DOI if available. (i.e. please cite the bitbucket repo the same way as the GigaDB data, see above).

If data and code has been modified in the revision process please be sure to update the public versions (e.g. in github or bitbucket) of this too."

Bitbucket repository was added to the references and cited in the "Availability of supporting data and materials" section of the manuscript.

"3) We seem to have the tables and parameters in the supplemental files in PDF format only. I feel it would be better for re-use and reproducibility if these data and parameters are made available in re-usable, tabulated form (you may be able host them in GigaDB or bitbucket - our data curators can give advice if needed)."

Supplementary tables are presented in both formats (PDF and tabular format) in GigaDB dataset.

"4) Please register your new software application in the bio.tools and SciCrunch.org databases to receive RRID (Research Resource Identification Initiative ID) and biotoolsID identifiers, and include these in the "availability and requirements" section of your manuscript. This will facilitate tracking, reproducibility and re-use of your tool."

We have registered GADMA in this databases and included received ID's in the "Availability of source code and requirements" section of the manuscript.

"5) At this stage, please remove any yellow highlighting that was made for the purpose of peer review."

We removed highlighting.

Close
