# Supplementary material for: GADMA: Genetic algorithm for inferring demographic history of multiple populations from allele frequency spectrum data
Source: Gigascience. 2020 Feb 29;9(3):giaa005. doi: 10.1093/gigascience/giaa005 (PMC7049072; doi:10.1093/gigascience/giaa005)
Supplement: giaa005_Reviewer_1_Report_Original_Submission — Ryan Gutenkunst -- 5/15/2019 Reviewed [file giaa005_reviewer_1_report_original_submission.pdf]

Reviewer Report

Title: GADMA: Genetic algorithm for inferring demographic history of multiple populations from allele frequency spectrum data

Version: Original Submission **Date:** 5/15/2019

Reviewer name: Ryan Gutenkunst

Reviewer Comments to Author:

To avoid needless obfuscation, I am Ryan Gutenkunst, developer of dadi, and I previously reviewed this manuscript for another journal.

Overall, the authors' approach has value. The optimization and model selection step of using dadi and moments is certainly a major obstacle for many users. Applying a genetic algorithm is a sensible improvement to the existing local approaches, and I like the adaptive aspect of the genetic algorithm. In my previous review, I raised serious methodological concerns that the authors' had not addressed the issue of linkage in the inference. The authors did make changes to address my concerns, but critical details are lacking. Given those methodological concerns, I have not yet dug deeply into the applications sections.

1) The authors have now adopted a composite likelihood version of AIC, but implementation of such is non-trivial, and the authors omit crucial details. In particular, calculating the CLAIC involves an expectation over dL/dtheta. This is non-trivial to calculate, because it is zero at the maximum CL point. How are the authors calculating it? (In our own work on composite likelihood statistics, this required bootstrapping the data, which, as I note below, is complex.)

2) The authors claim that the 95% confidence intervals in Tables 2,4, and 5 are calculated via bootstrap. But they provide no details, and they appear to be calculating them incorrectly.

I note that their uncertainties in Table 4 remain much smaller than in Gutenkunst (2009). This suggests that the authors are ignoring the effects of linkage on the inference. Note that one cannot perform a proper bootstrap given only the AFS derived from the data. To account for linkage (and in the case of Gutenkunst (2009), projection) one must go back to the original genomic data to generate the pseudo-replicates, by bootstrapping over regions of the genome.

Going back to the original genome data may be out-of-scope for the authors' approach. That's fine, the authors should just emphasize that their approach is good for finding the maximum CL parameters and guiding model selection, but further work is needed for estimating confidence intervals.

3) It is unclear how modes of population size change are incorporated into the parameter genomes. Can parameter sets mutate between sudden, exponential, and linear growth in an interval?

Minor issues:

Page 3, bottom: The typical assumption is not one of sudden change of effective population size. Rather, the population is assumed to begin in equilibrium, in other words, to have been the same size for a long time.

Level of Interest

Please indicate how interesting you found the manuscript: Choose an item.

Quality of Written English

Please indicate the quality of language in the manuscript: Choose an item.

Declaration of Competing Interests

Please complete a declaration of competing interests, considering the following questions:

- Have you in the past five years received reimbursements, fees, funding, or salary from an organisation that may in any way gain or lose financially from the publication of this manuscript, either now or in the future?
- Do you hold any stocks or shares in an organisation that may in any way gain or lose financially from the publication of this manuscript, either now or in the future?
- Do you hold or are you currently applying for any patents relating to the content of the manuscript?
- Have you received reimbursements, fees, funding, or salary from an organization that holds or has applied for patents relating to the content of the manuscript?
- Do you have any other financial competing interests?
- Do you have any non-financial competing interests in relation to this paper?

If you can answer no to all of the above, write 'I declare that I have no competing interests' below. If your reply is yes to any, please give details below.

I declare that I have no competing interests

I agree to the open peer review policy of the journal. I understand that my name will be included on my report to the authors and, if the manuscript is accepted for publication, my named report including any attachments I upload will be posted on the website along with the authors' responses. I agree for my report to be made available under an Open Access Creative Commons CC-BY license (http://creativecommons.org/licenses/by/4.0/). I understand that any comments which I do not wish to be included in my named report can be included as confidential comments to the editors, which will not be published.

Choose an item.

To further support our reviewers, we have joined with Publons, where you can gain additional credit to further highlight your hard work (see: https://publons.com/journal/530/gigascience). On publication of this paper, your review will be automatically added to Publons, you can then choose whether or not to claim your Publons credit. I understand this statement.

Yes Choose an item.
