# Supplementary material for: GADMA: Genetic algorithm for inferring demographic history of multiple populations from allele frequency spectrum data
Source: Gigascience. 2020 Feb 29;9(3):giaa005. doi: 10.1093/gigascience/giaa005 (PMC7049072; doi:10.1093/gigascience/giaa005)
Supplement: giaa005_Reviewer_1_Report_Revision_1 — Ryan Gutenkunst -- 9/27/2019 Reviewed [file giaa005_reviewer_1_report_revision_1.pdf]

Reviewer Report

Title: GADMA: Genetic algorithm for inferring demographic history of multiple populations from allele frequency spectrum data

Version: Revision 1 **Date:** 9/27/2019

Reviewer name: Ryan Gutenkunst

Reviewer Comments to Author:

I am relieved that the authors have taken my concerns to heart and fixed the major issues related to linkage in the data. With that major methodological flaw corrected, I have futher concerns regarding the manuscript, particularly the discussion of composite likelihood.

1) Page 3, Discussion of inputs to the method: If the authors are carrying out the CLAIC test correctly, then the AFS A and the constants C are not sufficient to fully specify the problem. The collection of bootstrapped data sets is also a necessary input. The authors should make this explicit.

2) More broadly, the authors should carefully discuss the need for block-bootstrapped data as input to the CLAIC calculation. This is a critical point that the authors themselves missed in the first two submissions of the manuscript. They should be very explicit about it in the present manuscript, so as not to mislead users, like they themselves were misled.

3) In addition to discussing proper block bootstrapping in the manuscript, it must be described in the GADMA manual. As far as I can tell, the only mention of bootstraps in the documentation is in the example_params file, where many users may be confused or overlook it.

4) For the human analysis, the authors should be explicit about how the bootstrapping was done for these data. I recognize that it is parroting Gutenkunst (2009), but explicit is better than implicit here.

5) Page 4: I am confused by the number of parameters in the specification. For example, in a two population interval I count 6 parameters (2 migration rates, 2 final population sizes, and 2 growth modes). What is the 7th parameter? I ask the authors to be much more explicit about how they are parameterizing the models, perhaps using Figure 5 as an example.

6) Page 5, Mutation of the demographic model: The described process only applies to continuous parameters. Is the mode of population size change (instant, growth, exponential) also allowed to mutate? If so, how? (I raised this question in my initial review, which the authors ignored.)

7) Page 6, Increasing model complexity: Is this feature used in any of the examples the authors present? From the Results section, it appears the authors have pre-specified the model structure for all analyses, rather than allowing the algorithm to do it. If this is the case, I suggest the authors remove this section. If they have indeed used this feature, they should be more clear when describing their results.

8) To shorten the manuscript, I suggest that many extraneous figures be removed. Figures 1,2,4,6,7 add very little to the exposition.

Level of Interest

Please indicate how interesting you found the manuscript: Choose an item.

Quality of Written English

Please indicate the quality of language in the manuscript: Choose an item.

Declaration of Competing Interests

Please complete a declaration of competing interests, considering the following questions:

- Have you in the past five years received reimbursements, fees, funding, or salary from an organisation that may in any way gain or lose financially from the publication of this manuscript, either now or in the future?
- Do you hold any stocks or shares in an organisation that may in any way gain or lose financially from the publication of this manuscript, either now or in the future?
- Do you hold or are you currently applying for any patents relating to the content of the manuscript?
- Have you received reimbursements, fees, funding, or salary from an organization that holds or has applied for patents relating to the content of the manuscript?
- Do you have any other financial competing interests?
- Do you have any non-financial competing interests in relation to this paper?

If you can answer no to all of the above, write 'I declare that I have no competing interests' below. If your reply is yes to any, please give details below.

I declare that I have no competing interests

I agree to the open peer review policy of the journal. I understand that my name will be included on my report to the authors and, if the manuscript is accepted for publication, my named report including any attachments I upload will be posted on the website along with the authors' responses. I agree for my report to be made available under an Open Access Creative Commons CC-BY license (http://creativecommons.org/licenses/by/4.0/). I understand that any comments which I do not wish to be included in my named report can be included as confidential comments to the editors, which will not be published.

Choose an item.

To further support our reviewers, we have joined with Publons, where you can gain additional credit to further highlight your hard work (see: https://publons.com/journal/530/gigascience). On publication of this paper, your review will be automatically added to Publons, you can then choose whether or not to claim your Publons credit. I understand this statement.

Yes Choose an item.
